# Thermostable proteins bioprocesses: The activity of restriction endonuclease-methyltransferase from *Thermus thermophilus* (RM.TthHB27I) cloned in *Escherichia coli* is critically affected by the codon composition of the synthetic gene

**DOI:** 10.1371/journal.pone.0186633

**Published:** 2017-10-17

**Authors:** Daria Krefft, Aliaksei Papkov, Agnieszka Zylicz-Stachula, Piotr M. Skowron

**Affiliations:** Department of Molecular Biotechnology, Faculty of Chemistry, University of Gdansk, Wita Stwosza 63, Gdansk, Poland; Universität Stuttgart, GERMANY

## Abstract

Obtaining thermostable enzymes (thermozymes) is an important aspect of biotechnology. As thermophiles have adapted their genomes to high temperatures, their cloned genes’ expression in mesophiles is problematic. This is mainly due to their high GC content, which leads to the formation of unfavorable secondary mRNA structures and codon usage in *Escherichia coli* (*E*. *coli*). RM.TthHB27I is a member of a family of bifunctional thermozymes, containing a restriction endonuclease (REase) and a methyltransferase (MTase) in a single polypeptide. *Thermus thermophilus* HB27 (*T*. *thermophilus*) produces low amounts of RM.TthHB27I with a unique DNA cleavage specificity. We have previously cloned the wild type (wt) gene into *E*. *coli*, which increased the production of RM.TthHB27I over 100-fold. However, its enzymatic activities were extremely low for an ORF expressed under a T7 promoter. We have designed and cloned a fully synthetic *tthHB27IRM* gene, using a modified ‘codon randomization’ strategy. Codons with a high GC content and of low occurrence in *E*. *coli* were eliminated. We incorporated a stem-loop circuit, devised to negatively control the expression of this highly toxic gene by partially hiding the ribosome-binding site (RBS) and START codon in mRNA secondary structures. Despite having optimized 59% of codons, the amount of produced RM.TthHB27I protein was similar for both recombinant *tthHB27IRM* gene variants. Moreover, the recombinant wt RM.TthHB27I is very unstable, while the RM.TthHB27I resulting from the expression of the synthetic gene exhibited enzymatic activities and stability equal to the native thermozyme isolated from *T*. *thermophilus*. Thus, we have developed an efficient purification protocol using the synthetic *tthHB27IRM* gene variant only. This suggests the effect of co-translational folding kinetics, possibly affected by the frequency of translational errors. The availability of active RM.TthHB27I is of practical importance in molecular biotechnology, extending the palette of available REase specificities.

## Introduction

Protein biosynthesis based on cloned heterologous genes is often lower than expected due to a different cytoplasmic environment in the recombinant host. This problem especially concerns genes originating from thermophilic bacteria, which thrive at temperatures of 50–121°C. Temperature and DNA stability imposes evolutionary adaptation pressures on the genomes, transcriptomes and proteomes of thermophilic bacteria. One of the observed characteristics is the increased GC content of their genomes as a function of growth temperature.

As a result, the pattern of codon usage by thermophilic bacteria is very different to that of mesophilic ones, such as the most frequently used recombinant host—*E*. *coli* [1–6]. As the differences in codon usage between species can adversely affect recombinant gene expression levels, various gene optimization strategies are used.

However, the strategy using codons of the highest frequency in the genome, or from the highly expressed gene subset of the host, does not always lead to improved gene expression [7–8]. Pushing the expression system for maximum biosynthesis of a toxic protein may result in bacterial cell lysis and decreased stability of the recombinant genetic construct due to the accumulation of mutations and translational errors. Thus, a proper balance between protein expression system stability and high level of recombinant protein production should be established for each particular case. Two general strategies for codon optimization are known, typically aided by specialised software [9]. The 'one aa—one codon' method selects a single codon for every aa from the gene to be optimized. The selection is based on similar criteria, such as: (*i*) most abundant codon in the recombinant host genome or (*ii*) a set of selected genes, typically the highly expressed ones [10]. Once the codon-aa pair is selected, there is only one possible combination of an optimized gene nucleotide (nt) sequence. The second method, 'codon randomization', uses one or more codons for each aa. The codon selection is based on the same criteria as the previous method; however, each codon has an user-assigned proportion of incorporation into a designed gene [11–15]. As opposed to the first strategy, a vast number of coding sequences can be designed [10]. Inherently, variants will not yield the same expression levels, as other factors affecting codon context, mRNA and translation processes play a role as well. However, further nt sequence fine-tuning can be implemented, without altering the final aa sequence. This includes modifying secondary and tertiary mRNA structure by reducing internal base pairing, which may hide the start codon, RBS or stall ribosome translocations. Other factors, such as codon context can affect expression level as the apparent result of neighbouring tRNA-tRNA steric interactions within the ribosome [11, 16–18] or a stretch of the same codons may cause ribosome stalling or slowing by depletion of the specific aminoacylo-tRNA pool. Gene expression is also affected by the position of the optimized codon. It has been shown that optimization of the initial 15–25 codons of the ORF is sufficient for substantial expression boosts in a recombinant host. If rare codons are present in the proximity of the start codon, their inhibitory effect on translation rate is particularly strong in both *E*. *coli* and *Saccharomyces cerevisae* [8]. It has been shown that replacing the TTG initiation codon with an ATG codon resulted in high-level expression of a previously silent heterologous gene in *E*. *coli* [19]. To address the potential problems mentioned above selection of properly engineered recombinant host, silencing endogenous proteolytic enzymes or co-expression of cloned chaperons may highly stimulate recombinant protein production [20].

To accumulate possible problems to test within a single target, we selected a very toxic gene–coding for DNA cleaving REase-MTase RM.TthHB27I, which originates from a high GC content thermophile. RM.TthHB27I protein is a member of the *Thermus-*family of atypical, bifunctional thermozymes, which was characterized by our group [21–24]. The selected Type IIC/IIG/IIS REase-MTase recognizes asymmetric 5’-CAARCA-3’ DNA sequences and cleaves 11/9 nt downstream [24, 25]. Similarly, to other members of the *Thermus*-family, TthHB27I REase activity and DNA recognition specificity is affected by S-adenosyl-L-methionine (SAM) or its analogues [22–24]. In this paper we describe the successful optimization of this toxic for bacterial host gene, using a modified ‘codon randomization’ strategy. The implications and the usefulness of the strategy presented are beyond simple expression optimization experiments. The strategy affects the proper folding of the thermozymes to bioactive states.

## Materials and methods

### Bacterial strains, plasmids, media and reagents

*E*. *coli* TOP10 {F^-^ *mcr*A Δ(*mrr*-*hsd*RMS-*mcr*BC) φ80*lac*ZΔM15 Δ*lac*X74 *nup*G *rec*A1 *ara*D139 Δ(*ara-leu*)7697 *gal*E15 *gal*K16 *rps*L(Str^R^) *end*A1 λ^-^} (Invitrogen, Carlsbad, CA, USA) was used for gene cloning and DNA purification. Bacteria were grown in LB medium [26]. For protein expression the T7 promoter-based pET21d(+) vector (Novagen, Madison, WI, USA) and *E*. *coli* BL21(DE3) {F^−^*omp*T *hsd*SB(r_B_^–^, m_B_^–^) *gal dcm* (DE3)} were utilized (Life Technologies, Carlsbad, CA, USA). Recombinant bacteria were grown in Terrific Broth (TB) medium [26]. Components of the media used [26] were from Becton-Dickinson (Franklin Lakes, NJ, USA) and supplemented with ampicillin (100 μg/ml). Phosphocellulose P11 and DEAE-cellulose chromatographic media were from Whatman (Springfield Mill, UK). Affinity resin Heparin-agarose was from GE Healthcare (Uppsala, Sweden). Agarose was from FMC (Rockland, NY, USA). DNA isolation kits (DNA Cleanup Micro Kit, GeneJet Plasmid Miniprep Kit and GeneJET Gel Extraction), DNA size markers (100 bp Plus DNA Ladder, GeneRuler 1 kb DNA Ladder), protein molecular weight standards (PageRuler™ Unstained Broad Range Protein Ladder and Pierce™ Unstained Protein Molecular Weight Marker) were from Thermo Fisher Scientific/Fermentas (Vilnius, Lithuania). The proofreading Marathon DNA Polymerase was from A&A Biotechnology (Gdynia, Poland). SalI and BsaI REases were from New England Biolabs (Ipswich, MA, USA). T4 DNA Ligase was from Epicentre (Madison, WI, USA). Other reagents were from POCh S.A. (Gliwice, Poland), Sigma-Aldrich (St. Louis, MO, USA), AppliChem Inc. (St. Louis Missouri, MO, USA) or Fluka Chemie GmbH (Buchs, Switzerland). The deoxyoligonucleotides (oligo) were synthesized by and DNA sequencing services were conducted at Genomed S.A. (Warsaw, Poland). Amicon ultrafiltration devices and 30 kDa cut-off RC filters were from Millipore Corporation (Billerica, MA, USA).

### Expression of synthetic *tthHB27IRM* gene in *E*. *coli*

For analytical protein expression experiments 100 ml *E*. *coli* BL21(DE3) cultures, carrying either pET21d(+)-wt-*tthHB27IRM* or pET21d(+)-synthetic *tthHB27IRM*, were grown in TB medium [26], supplemented with 100 μg/ml ampicillin, at temperatures of 30°C, 37°C, 42°C, 46°C, with vigorous aeration. Cultures were induced by adding 1 mM IPTG, when the OD_600_ reached 0.6. The culture growth was continued for 6 hours (h) after induction. Samples from both the control (cells with the vector without insert), non-induced and induced cultures were subjected to SDS-PAGE electrophoresis. The gels were analysed for the appearance of the expected band size of ~117–128 kDa [24] (GenBank accession no. AE017221.1). The best conditions of cultivation were selected for a large-scale bacterial culture. For large-scale expression, 1 L TB/ampicillin medium was inoculated with bacteria washed out from a Petri dish. The culture was grown at 30°C with vigorous aeration, followed by IPTG induction (1 mM), when the OD_600_ reached 0.6 and continued for 3 h at 30°C, cooled down to 4°C and cells were recovered by centrifugation. The yield was 3.5 g from 1 L of bacterial culture with pET21d(+)-synthetic *tthHB27IRM*.

### Purification of synthetic RM.TthHB27I thermozyme

The purification scheme in this work varied from the scheme described previously for native wt RM.TthHB27I enzyme [24] in the following steps:

1. A heat denaturation step after the polyethyleneimine (PEI) treatment. It included the PEI supernatant incubation for 30 min at 65°C, divided into 30 ml portions in 250 ml glass Erlenmeyer flasks, which were gently rotated every minute in a water bath. The use of glass instead of plastic, container geometry and rotation was essential for adequate heat transfer and denaturation of thermolabile *E*. *coli* proteins, including non-specific nucleases. The denatured and aggregated proteins were removed by centrifugation.
2. The order of heparin-agarose chromatography and Phosphocellulose P11 chromatography was reversed.
3. The gel filtration step was omitted.

### REase and MTase enzymatic assays

The cleavage reactions were performed in 50 μl volumes of RM.TthHB27I REase buffer (10 mM Tris-HCl pH 7.0 at 65°C, 6 mM βME, 6 mM MgCl_2_, 40 mM NaCl, bovine serum albumin (BSA) 0.1 mg/ml), supplemented with 100 μM SAM and DNA substrates. SAM enhances the activity of this 'slow' thermozyme. One unit of activity of RM.TthHB27I REase is defined as the minimal amount of thermozyme needed to hydrolyse 1 μg of bacteriophage λ DNA in 1 h at 65°C in 50 μl of RM.TthHB27I REase buffer, supplemented with 100 μM SAM, resulting in a stable partial DNA digestion pattern. The REase assay was used to test of the effects of the cofactor and its analogues on synthetic RM.TthHB27I REase activity. The reactions were performed at 65°C in 50 μl of RM.TthHB27I REase buffer supplemented with either 50 μM SAM, sinefungin (SIN), S-adenosyl-L-cysteine (SAC), S-adenosylhomocysteine (SAH) or ATP. The 1789 bp PCR product [24] was used as a DNA substrate in amount of 0.5 μg. This substrate contains two convergently oriented RM.TthHB27I recognition sites, with relatively long flanking DNA. This design was made in order to generate an efficient substrate, allowing to observe fine differences in the thermozyme’s activity.

RM.TthHB27I methylation of DNA by recombinant thermozyme was assayed *in vitro* under various buffer conditions. The basic buffer contained 10 mM Tris-HCl (pH 7.0 at 65°C), 40 mM NaCl, 6 mM βME and 0.1 mg/ml BSA, supplemented immediately prior to reactions with 100 μM SAM and either 6 mM MgCl_2_ or 6 mM CaCl_2_ or 3 mM EDTA. The methylation reaction was conducted in a volume of 50 μl for 6 h at 65°C, then the reaction mixture was subjected to proteinase K digestion for 1 h at 55°C, and the DNA was phenol-chloroform extracted and ethanol precipitated. The purified DNA was digested for 1 h with 2 units of synthetic RM.TthHB27I described in the REase assay and analysed by agarose gel electrophoresis.

## Results

### Design and cloning of a synthetic *tthHB27IRM* gene

We have shown previously that decreasing the overall GC content, reduction of mRNA secondary structures, avoiding repetition of the same codons and other codon contexts obstacles lead to increased thermophile-derived RM.TaqII biosynthesis by approximately 10-fold [23]. The RM.TaqII enzyme is closely related to RM.TthHB27I. Moreover, we have recently solved a longstanding riddle (since 1984) of two RM.TaqII specificities, discovering the third, related RM system (RM.TaqIII) in *T*. *aquaticus* YT-1 [27]. For the purpose of the *taqIIRM* gene optimization, a modified ‘one aa—one codon’ strategy, biased toward sub-optimal, low GC codons was used [23]. In that approach the correction of GC content was performed by using codons preferred by *E*. *coli*, which generally have a lower GC% in comparison to genes originating from thermophiles, biasing for *E*. *coli* Ser codon UCU even though it has lower occurrence than Ser UCC.

In this paper, we explored another gene optimization approach. This time, a synthetic *tthHB27IRM* gene was constructed (S1 File) using an alternative ‘codon randomization’ strategy, also modified toward biasing for codons with lower GC content. In general, we observed very low amounts of the thermozymes belonging to the *Thermus-*family of REases-MTases in *Thermus* sp. bacteria. In addition, a substantially lower expression of the cloned gene variants was obtained in comparison to the typical recombinant gene expressed in highly tuned, commercial *E*. *coli* expression systems [28, 29]. Thus, for biotechnological purposes, and to allow for the planned biochemical studies, further expression improvements were needed. Due to the previously described problems with recombinant protein stability, RM.TthHB27I was selected as an interesting target for optimization method development. Our aim was to improve the strategy of the biosynthesis of the enzymatically active proteins from thermophile-derived genes. We reported previously the cloning and overexpression of native wt (not optimized) *tthHB27IRM* gene in *E*. *coli*. We obtained an approximately 100-fold increase in the biosynthesis of RM.TthHB27I compared to the native wt *T*. *thermophilus* bacteria [24]. However, the enzymatic activity was much lower than expected from the substantial amount of approximately 120 kDa polypeptide, corresponding to RM.TthHB27I detected by SDS-PAGE. Moreover, the biochemical properties of the recombinant thermozyme were different from those of native wt RM.TthHB27I, purified from *T*. *thermophilus*. The protein was rapidly inactivated during purification attempts and became heat sensitive. We believed that the GC rich sequence, established with the use of foreign to *E*. *coli* codon usage, adversely affected transcription which, as a consequence, impaired translation.

We supposed that conversion of the wt *tthHB27IRM* gene to an artificial gene with a different nt sequence not only would improve the obtained recombinant protein level but may also have a positive effect on its enzymatic properties.

Design of the synthetic *tthHB27IRM* gene involved substantial changes in the nt sequence, while maintaining the original translated aa sequence (S2 File). We did not add any purification tag because it could affect the properties of recombinant RM.TthHB27I. The synthetic 3366 bp *tthHB27IRM* gene was designed and constructed, which included changing 661 of 1121 codons (59%). The new gene sequence differed from the wt gene by 21%. The wt *tthHB27IRM* gene is characterized by a high (61.5%) GC content. The 62.8% of the original *tthHB27IRM* codons (704 out of the total 1121) are rarely found in highly expressed *E*. *coli* genes (S1 Table) [30, 31, 32, 33]. Moreover, there are two potential start codons with accompanying RBSs, the first GTG and the second ATG, which potentially could compete for translation of recombinant wt *tthHB27IRM* mRNA, and may yield a mixture of variants of recombinant wt RM.TthHB27I: 1121 or 1106 aa long [24]. It has not been previously determined, which start codon (or both) is utilized in *T*. *thermophilus* and whether the two alternate forms of RM.TthHB27I protein exhibit variations in enzymatic activity. Thus, the synthetic *tthHB27IRM* was designed as a full-length gene, with the original GTG start replaced by ATG. S2 File presents the designed synthetic *tthHB27IRM* nt and aa sequences and their comparison to native and recombinant wt *tthHB27IRM* gene. The previously determined functional domains and motifs are also indicated [24].

**Fig 1:**
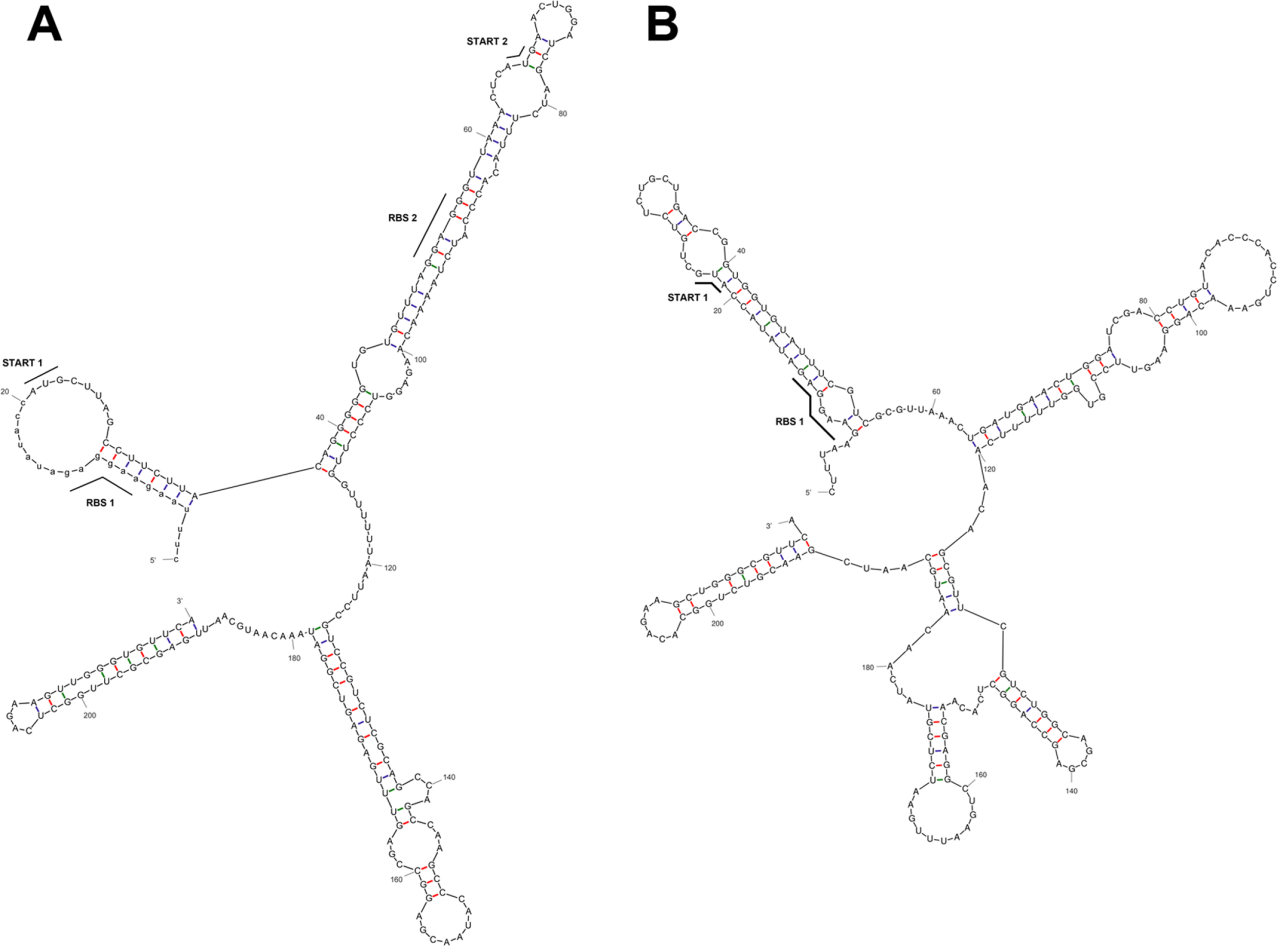
Predicted secondary structure of the first 200 nt of *tthHB27IRM* mRNA generated by Mfold Web Server [34, 35]. (A) Predicted structure of initial recombinant wt *tthHB27IRM* mRNA fragment before codon optimization (revised free energy: dG = -60.40 kcal/mol). (B) Structure of initial synthetic *tthHB27IRM* mRNA fragment after codon optimization (revised free energy: dG = -68.04 kcal/mol).

The optimization was conducted in two stages: (*i*) codon randomization and (*ii*) sequence scanning for mRNA secondary structures (Fig 1), codon clusters, local codon environment and further optimization of selected regions. S1 Table summarizes the selected codons for the synthetic *tthHB27IRM* construct along with the used moderate bias of codons for Ala, Asn, Asp, Cys, Gly, Ile, Phe, Pro, Ser, Thr, Val, toward lower GC content compared to the *E*. *coli* fraction of relative occurrence of the codon in its synonymous codon family. The codon table prior to AT-biasing was based on Wisconsin Package, Genetics Computer Group [30] as it performed very well in our experience with over one hundred cloned and expressed genes (Skowron et al., unpublished results). Other tables with codons for highly expressed *E*. *coli* genes are in use, most notably those described in a series of papers by Sharp et al.: from 1986 [31], based on 27 very highly expressed genes and 15 highly expressed genes; 1988 [32], based on 10% of genes with the highest expression in *E*. *coli* and 2010 [33], based on 40 highly expressed genes.

In addition, codons of relatively low occurrence in *E*. *coli* were eliminated from the synonymous codons sets used here, with the cut-off value of 0.13–0.16 occurrence (S1 Table). Besides codon optimization, the overall GC content was decreased by 6%, reaching 55.5% GC. Any further GC content decrease was limited by the aa sequence of the RM.TthHB27I protein, suggesting that certain aa are preferred by thermophilic microorganisms. Optimization of mRNA was aided by bioinformatic prediction of secondary structures (Mfold Web Server [34, 35]) and manual assessment. The first 200 nt of mRNA’s, coding for recombinant wt *tthHB27IRM* and synthetic *tthHB27IRM* genes are compared in Fig 1. Another departure from the strategy which we have described previously [23] for optimizing the cloned synthetic *taqIIRM* gene, was the inclusion of a negative translation regulation mRNA 5’-segment, which also constituted a part of the synthetic *tthHB27IRM* coding gene. We have anticipated that it would impose limits on the translation initiation rate of synthetic *tthHB27IRM* in order to prevent excessive synthetic RM.TthHB27I biosynthesis and toxic effects, such as those which we observed in the case of the recombinant wt *tthHB27IRM* [24]. In general, recombinant DNA-interacting proteins are toxic to recombinant host, if expressed at high level [36, 37]. In recombinant wt *tthHB27IRM* mRNA, the first RBS is split between a double stranded (ds) mRNA stem and ss mRNA loop, while the corresponding start codon is entirely located within the ss loop, thus is highly accessible. The second RBS is embedded within ds mRNA and from the corresponding start codon a single, initial base is located in another ss mRNA loop. Such a natural setup does not give a clear answer, which start codon is preferred (Fig 1) [24]. This setup was changed for synthetic *tthHB27IRM*: the first RBS remained partially exposed, while the corresponding start codon has two bases located within the ds RNA stem-loop. The second RBS was eliminated to quench the potential competition with the first one. Overall, the first 200 nt of recombinant wt *tthHB27IRM* mRNA exhibit a substantial stability of ds RNA helix (revised free energy: dG = -60.40 kcal/mol), while the lower GC content, synthetic *tthHB27IRM* mRNA ds structure has higher stability (revised free energy dG = -68.04 kcal/mol (Fig 1).

### Biosynthesis of biologically active recombinant RM.TthHB27I in mesophilic *E*. *coli*

#### Optimization of the synthetic *tthHB27IRM* gene expression

All the investigated REases-MTases belonging to the *Thermus*-family are encoded by genes whose expression levels are very low and their purification is further complicated by the presence of thermostable contaminating proteins, pigments and other cell components. Such contaminants cannot be easily removed. We have previously published the cloning and expression of recombinant wt *tthHB27IRM* gene [24]. The gene exhibited a strong toxic effect on *E*. *coli* during transformation–an over 1000-fold decrease of transformation efficiency, when transformed cells were incubated at 37–42°C compared to 30°C. The experiment was conducted under non-inducing conditions. It probably means that the residual transcription/translation generated small amounts of the recombinant wt RM.TthHB27I molecules, cleaving DNA *in vivo*. Even such a small amount of the active protein obviously exceeded the repair system’s capabilities. Thus, expression temperature and induction timing had to be optimized for synthetic *tthHB27IRM* expression.

**Fig 2:**
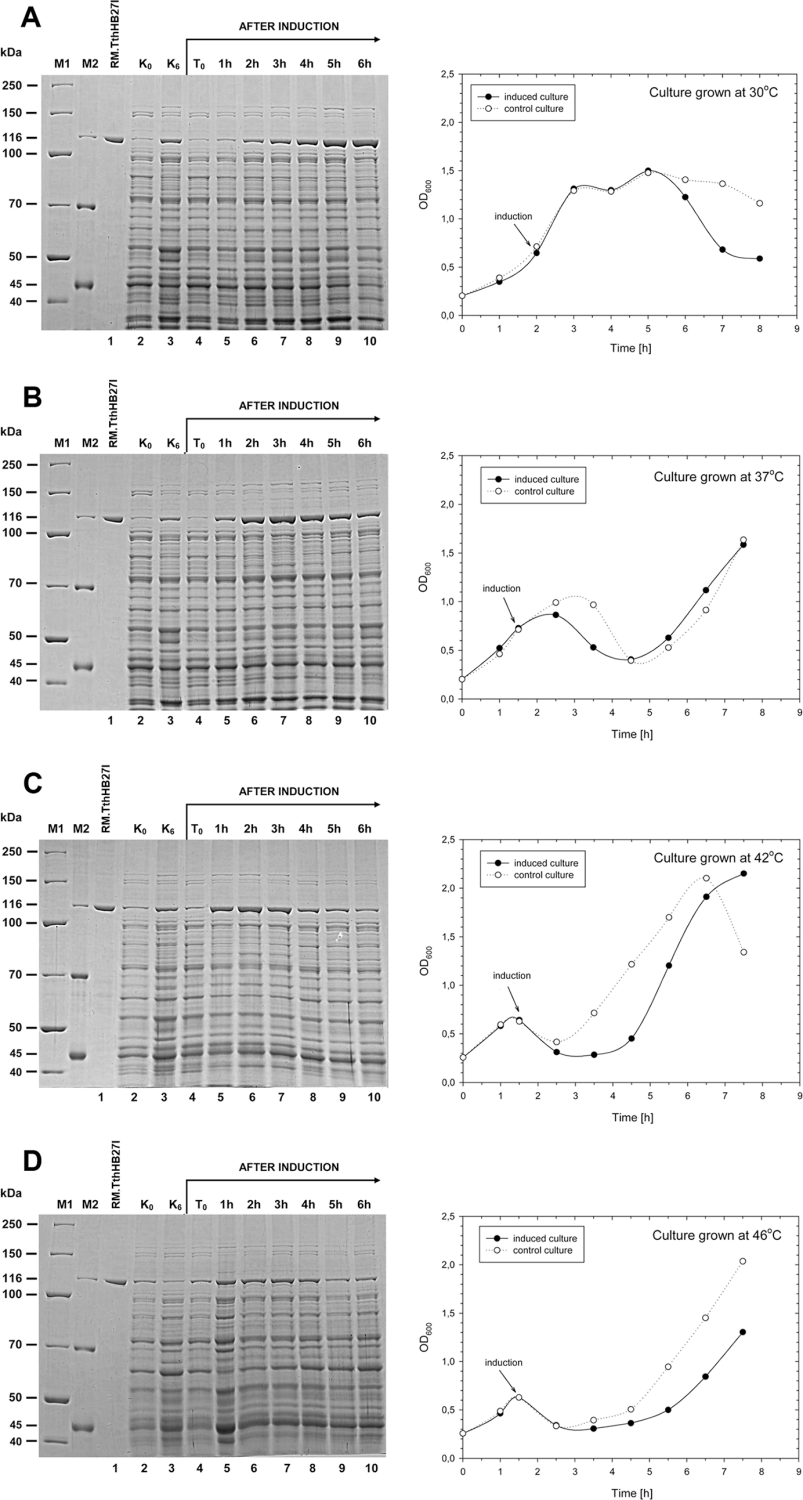
Expression of synthetic *tthHB27IRM* gene as a function of temperature. (A) Kinetics of *E*. *coli* [pET21d(+)-synthetic *tthHB27IRM*] bacterial cultures growth and synthetic RM.TthHB27I protein expression at 30°C. The recombinant *E*. *coli* BL21(DE3) cultures were cultivated in TB media at 30°C with vigorous aeration. After induction (OD_600_ = 0.6–0.7) with IPTG cultures were grown for 6 h. Culture samples were taken at 1 h intervals and subjected to spectrophotometric analysis and SDS-PAGE. Cells were lysed and lysates were analysed using 7.5% SDS-PAGE. Lane M1, PageRuler™ Unstained Broad Range Protein Ladder (Thermo Fisher Scientific/Fermentas); lane M2, Pierce™ Unstained Protein Molecular Weight Marker (Thermo Fisher Scientific/Fermentas); lane 1, synthetic RM.TthHB27I protein; lane 2, separate control culture *E*. *coli* [pET21d(+)-synthetic *tthHB27IRM*] cultivated at 30°C, before induction (OD_600_ = 0.6); lane 3, control culture—6 h after induction; lane 4, *E*. *coli* [pET21d(+)-synthetic *tthHB27IRM*] experimental culture, before induction (OD_600_ = 0.6); lane 5, 1 h after induction; lane 6, 2 h; lane 7, 3 h; lane 8, 4 h; lane 9, 5 h; lane 10, 6 h. (B) Experiment conducted as in (A), but at 37°C. (C) Experiment conducted as in (A), but at 42°C. (D) Experiment conducted as in (A), but at 46°C.

For this purpose, a series of cultures were prepared, grown at different temperatures: 30°C, 37°C, 42°C, 46°C and induced with IPTG when the OD_600_ reached 0.6, thus in early-medium logarithmic stage (Fig 2). Comparative SDS-PAGE analysis was conducted for every culture time point and the results are presented on the corresponding graphs with the amount of protein loaded in proportion to the same culture volume (Fig 2). Based on previous results, it was expected that: (*i*) elevated temperature prior to induction will further activate the synthetic RM.TthHB27I *in vivo* and cells would either grow very poorly or not at all, (*ii*) induction would cause rapid cells lysis compared to the experiment conducted at 30°C.

The culture grown at 30°C behaved as expected–the bacteria stopped growing 3 h after induction at OD_600_ = 1.4 and started to gradually lyse, dropping to OD_600_ = 0.5 after 8 h culturing (6 h after induction) (Fig 2A). The cultures grown at 37–46°C behaved somewhat surprisingly (Fig 2B, 2C and 2D). Once transformed, the bacteria managed to grow at 37–46°C (induced or non-induced). Interestingly, the bacteria continue to grow even after induction with IPTG, instead of lysing, as observed for the induced 30°C culture (Fig 2B, 2C and 2D).

A possible explanation of this observation is that the cells, which survived initial introduction *via* transformation of the plasmid carrying synthetic *tthHB27IRM* gene, managed to cope with the initial out-of-balance methylation versus restriction of the host’s unprotected genome by synthetic RM.TthHB27I, as the thermozyme is fused REase-MTase. The MTase component of the thermozyme would further partly stabilize the protected genome, once initial methylation has outcompeted the REase component. The stabilization apparently was maintained, as it allowed for further *E*. *coli* cultivations at elevated temperatures through many generations. In our opinion, this scenario would somewhat corroborate Kobayashi’s hypothesis of RM system acting as ‘minimal forms of life’ and ‘selfish genes’ [38], which kill the host if the RM existence within a cell would be jeopardised. In the case of the synthetic RM.TthHB27I, this fusion RM system kills the recombinant *E*. *coli* host, unless it manages to cope with the initial out-of-balance methylation/restriction and remains in the transformed cells.

The culture grown at 30°C showed a steady accumulation of the recombinant synthetic RM.TthHB27I, which becomes the dominant polypeptide in the bacterial lysate 3 h after induction (Fig 2A). The maximum amount of the recombinant protein was detected 6 h after induction. This precisely matched the timing of bacterial lysis. In contrast, in the culture grown at 37–46°C, the synthetic RM.TthHB27I band becomes dominant after the first hour (Fig 2B, 2C and 2D). The amount of the protein, however, gradually decreased, starting from 3–4 h after induction, and dropped several fold by the sixth hour.

A number of interpretations of the described phenomenon can be given, including:

1. Synthetic RM.TthHB27I methylation activity at 30°C does not keep up with the course of biosynthesis of the thermozyme and its restriction activity, while at 37–46°C the MTase is activated to the point where complete chromosome protection becomes possible.
2. The recombinant, thermostable synthetic RM.TthHB27I is not folded correctly at 30°C, being deficient in the MTase domain, while the REase domain is properly folded, thus active.
3. We hypothesize that at elevated temperatures, the host’s self-defence mechanisms or ‘minimal form of life’ self-regulatory mechanisms [38] are activated and cells manage to depress the biosynthesis of synthetic RM.TthHB27I protein to the point of cells’ survival ability at a given temperature. The mechanism of this phenomenon has not been determined;
4. The appearance and further domination of synthetic *tthHB27IRM* gene mutations cause inactivation of the REase activity. This hypothesis, however, seems rather remote, as the timing of the mutations’ appearance would be unpredictable, while we have observed the same culture curves in several independent experiments.

The above observations also have implications, important from the standpoint of biotechnology. All the experiments presented in Fig 2 were performed using a peptone-based medium. Using the tryptone-based medium, we saw much lower T7 promoter leakage and the synthetic RM.TthHB27I accumulation after induction took longer (not shown). Nevertheless, we decided to use soy-derived peptone medium for preparative purposes because of the higher final synthetic RM.TthHB27I yield. Regardless of the reasons for expression level and culture behaviour, variations at different temperatures and media, high expression of the synthetic *tthHB27IRM* gene was achieved.

#### Biochemical differences between RM.TthHB27I variants

**Fig 3:**
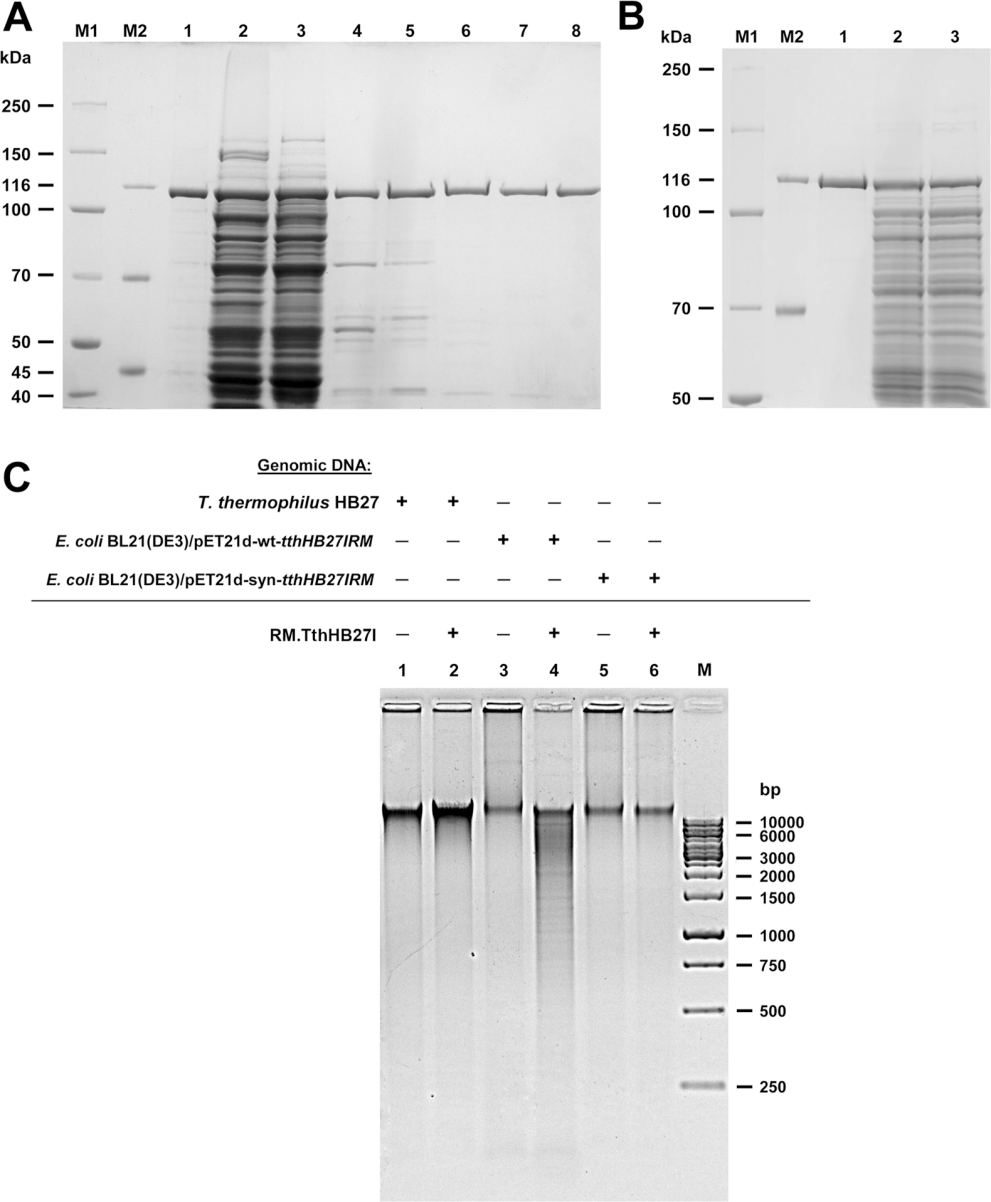
Biosynthesis and activity of synthetic RM.TthHB27I. (A) Isolation of synthetic RM.TthHB27I from *E*. *coli*. Lane M1, PageRuler™ Unstained Broad Range Protein Ladder; lane M2, Pierce™ Unstained Protein Molecular Weight Marker; lane 1, native wt RM.TthHB27I; lane 2, crude lysate from *E*. *coli* [pET21d(+)-synthetic *tthHB27IRM*], grown at 30°C; lane 3, supernatant after PEI treatment; lane 4, supernatant after incubation at 65°C; lane 5, 0–50% AmS fractionation cut; lane 6, DEAE-cellulose chromatography; lane 7, heparin-agarose chromatography; lane 8, Phosphocellulose P11 chromatography. (B) Yields of recombinant wt RM.TthHB27I and synthetic RM.TthHB27I biosynthesis. Recombinant *E*. *coli* BL21(DE3) strains were subjected to 3 h induction at OD_600_ = 0.6–0.7 and 30°C. Cells were lysed and protein lysates were analysed by 7.5% SDS-PAGE. Lanes M1, M2 as in (A); lane 1, synthetic RM.TthHB27I; lane 2, crude lysate from induced *E*. *coli* [pET21d(+)-wt-*tthHB27IRM*]; lane 3, crude lysate from induced *E*. *coli* [pET21d(+)-synthetic *tthHB27IRM*]. (C) Comparison of the activities of RM.TthHB27I MTase variants *in vivo*. 0.5 μg of total DNA from *T*. *thermophilus* or induced, recombinant *E*. *coli* BL21(DE3) strains were digested with 2 units of synthetic RM.TthHB27I in REase buffer+SAM for 1 h at 65°C. Lane 1, untreated *T*. *thermophilus* DNA; lane 2, *T*. *thermophilus* DNA digested with synthetic RM.TthHB27I; lane 3, untreated *E*. *coli* BL21(DE3) [pET21d(+)-wt-*tthHB27IRM*] DNA; lane 4, as in lane 3, but with synthetic RM.TthHB27I; lane 5, untreated *E*. *coli* BL21(DE3) [pET21d(+)-synthetic *tthHB27IRM*] DNA; lane 6, as in lane 5, but with synthetic RM.TthHB27I; lane M, GeneRuler 1 kb DNA Ladder.

To compare the amount of the produced recombinant RM.TthHB27I variants, quantification of the recombinant proteins was performed by densitometry of the corresponding protein bands on Coomassie blue stained SDS-PAGE gels. It appeared that both the investigated recombinant *E*. *coli* strains produced similar yields of the thermozyme variants (approx. 36–40 mg/L). However, bacterial culture with the recombinant wt RM.TthHB27I were slightly more abundant (Fig 3). Comparative SDS-PAGE of recombinant (wt and synthetic) RM.TthHB27I variants from the entire cells and soluble fractions indicated that both thermozyme variants were fully soluble (not shown). We hypothesize that the full biosynthesis potential of the synthetic RM.TthHB27I polypeptide, which was expected after codon optimization, was supressed by the sub-optimal design of the nt environment around the translation initiation signals, which are partially hidden within the ds mRNA 5’ end stem-loop region. Thus, fine-tuning of the ds mRNA structure at the 5’ end may be considered as an adequate method for controlling even an optimized synthetic gene. Nevertheless, the expression potential of synthetic RM.TthHB27I is a complicated issue, due to the protein’s high toxicity, since the host could exercise various mechanisms to decrease its expression, thus preventing from obtaining the anticipated biosynthesis level in the absence of a regulatory circuit.

Regardless of the expression levels, there were radical biochemical differences between recombinant wt RM.TthHB27I compared to native wt *T*. *thermophilus*-isolated thermozyme or synthetic RM.TthHB27I. All purification steps, used successfully for the native wt thermozyme [24], such as: polyethyleneimine (PEI), ammonium sulphate (AmS), DEAE-cellulose chromatography, phosphocellulose chromatography, heparin-agarose chromatography and size-exclusion chromatography resulted in the inactivation or precipitation of the recombinant wt RM.TthHB27I thermozyme, regardless of the purification step order. In spite of the substantial expression of the recombinant wt RM.TthHB27I polypeptide (40.1 mg/L), its activity was detectable in crude cells lysates only and at a low level, which was unexpected, considering the substantial polypeptide biosynthesis [24]. Poor restriction activity *in vitro* was matched by defective RM.TthHB27I methylation *in vivo*, as the genome of *E*. *coli*, carrying the recombinant wt *tthHB27IRM* gene, was protected only partially, as opposed to the genomic DNA of *T*. *thermophilus* and *E*. *coli*, carrying the synthetic *tthHB27IRM* gene (Fig 3C). Essentially, the same purification protocol was applied for synthetic RM.TthHB27I as for the native wt thermozyme [24], with a minor modification that a heat treatment step at 65°C was added prior to chromatography. This additional step enabled the denaturation and removal of endogenous *E*. *coli* proteins (Fig 3A). Attempts to use heat treatment for *E*. *coli* cells carrying recombinant wt *tthHB27IRM* gene resulted in a complete loss of the thermozyme activity. Thus, the thermozyme variant apparently lost its thermostability. We suppose that the misfolded protein variant precipitated and/or adsorbed to the precipitating *E*. *coli* proteins. This indicates fundamental structural and functional differences between the recombinant thermozyme variants. We hypothesize that this may be due to incorrect folding of recombinant wt RM.TthHB27I, possibly exposing hydrophobic regions, thus the protein becomes prone to precipitation/adsorption. For aa sequences validation purposes, both native, wt recombinant RM.TthHB27I and synthetic variants were subjected to Mass Spectroscopy analysis (S3–S5 Files). Results obtained were with high coverage of 59%, 66% and 73%, respectively. In both recombinant RM.TthHB27I variants, the N-termini and overall sequence patterns were the same. This clearly confirms, that translation for both recombinant RM.TthHB27I variants starts at the start codon no 1, not 16 (S4 and S5 Files). Although native wt RM.TthHB27I variant analysis has not revealed the presence of N-terminal 15 aa segment, it cannot be completely excluded, that both translation variants co-exist.

#### Comparison of the native and recombinant RM.TthHB27I activities

**Fig 4:**
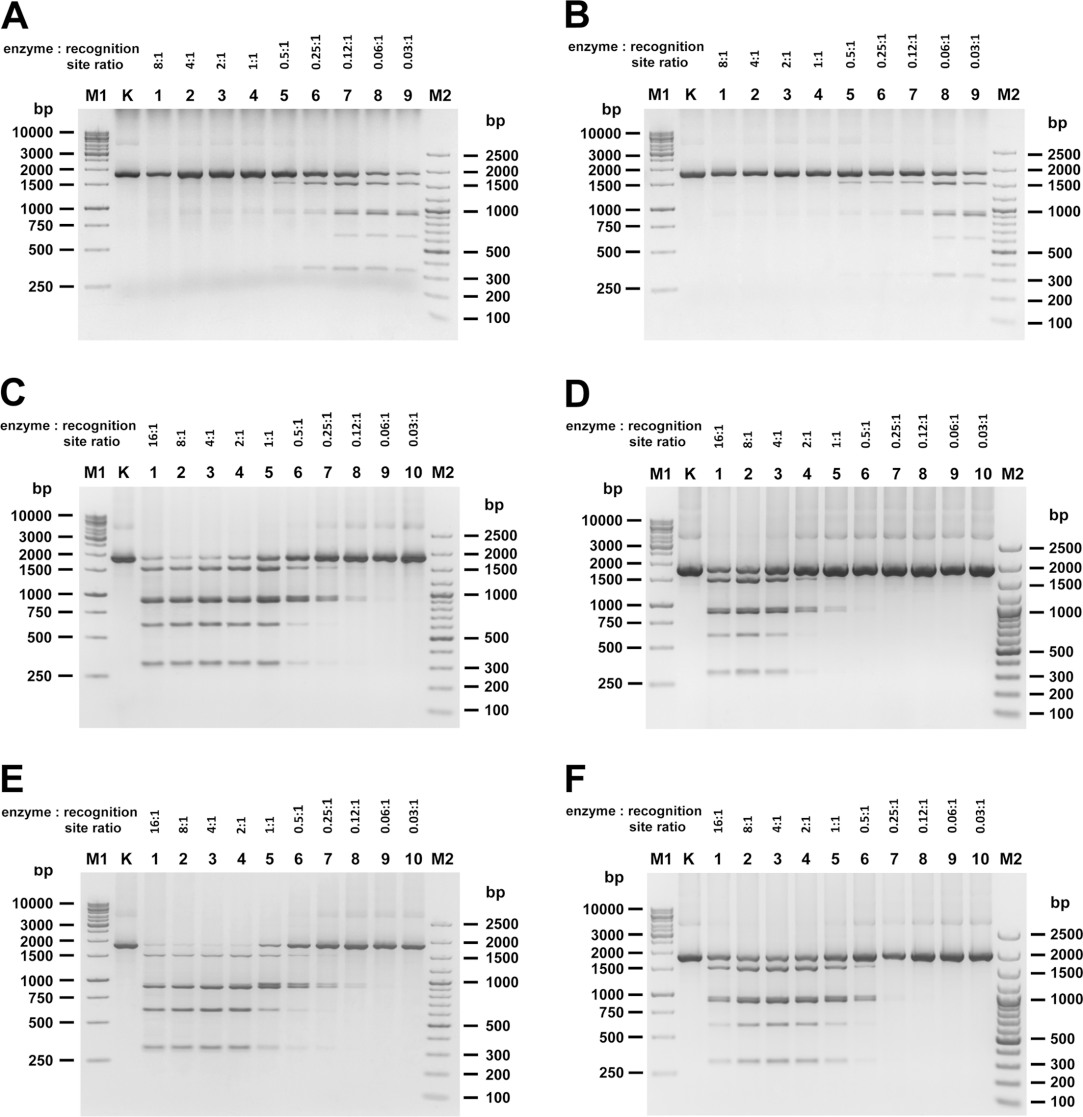
Comparison of the enzymatic activities of native wt RM.TthHB27I and recombinant synthetic RM.TthHB27I thermozymes. Lanes M1, GeneRuler 1 kb DNA Ladder; lanes M2, 100 bp Plus DNA Ladder; lanes K, untreated PCR fragment. (A) Titration of native wt RM.TthHB27I MTase activity. 0.5 μg of 1789 bp PCR DNA substrate was incubated for 6 h with decreasing amounts of native wt RM.TthHB27I in the MTase reaction buffer supplemented with 100 μM SAM and 6 mM Ca^2+^ at 65°C. Lanes 1–9, PCR fragment methylated with 2-fold serial dilutions of native wt RM.TthHB27I, starting from the thermozyme: recognition site molar ratio of 8: 1. (B) As in (A), but performed with synthetic RM.TthHB27I. (C) Titration of native wt RM.TthHB27I REase activity without cofactors/analogues. 0.5 μg of 1789 bp PCR DNA substrate was incubated for 1 h with decreasing amount of native wt RM.TthHB27I in REase buffer at 65°C. Lanes 1–10, PCR fragment digested with native wt RM.TthHB27I at the thermozyme: recognition site molar ratios shown above the lanes. (D) As in (C), except that performed with synthetic RM.TthHB27I. (E) Titration of native wt RM.TthHB27I REase activity in the presence of SAM. 0.5 μg of 1789 bp PCR DNA substrate was incubated for 1 h with decreasing amount of native wt RM.TthHB27I in REase buffer at 65°C. Lanes 1–10, PCR fragment digested with native wt RM.TthHB27I at the thermozyme: recognition site molar ratio shown above the lanes. (F) As in (E), except that performed with synthetic RM.TthHB27I.

To compare enzymatic activity, the units of the investigated RM.TthHB27I variants were titrated in the presence or absence of SAM. For that purpose, multiple series of 2- fold serial dilutions of the enzymes were prepared, keeping the DNA concentration constant. The titration reactions were performed for both the REase and MTase activity. The REase specific activity was estimated for all investigated protein preparations (Table 1). Finally, the optimal enzyme to substrate ratio was determined for both RM.TthHB27I activities (Fig 4 and Table 1).

**Table 1:** Comparison of RM.TthHB27I thermozyme production in native wt host *T*. *thermophilus* and in *E*. *coli* expressing both *tthHB27IRM* gene variants.

Expressed gene	Codon optimization	Amount of produced RM.TthHB27I (mg/L) ^1^	REase specific activity of purified RM.TthHB27I (units/mg) ^2^	Molar ratio thermozyme: recognition site for defined 1 u REase activity	MTase specific activity of purified RM.TthHB27I (units/mg) ^2^	Molar ratio thermozyme: recognition site for defined 1 u MTase activity
native wt *tthHB27IRM* (*T*. *thermophilus*)	n/a	<0.4	approx. 2600	2: 1	approx. 870	1: 1
recombinant wt *tthHB27IRM* (*E*. *coli*)	no	40.1	0 ^3^	n/a	n/a	n/a
synthetic *tthHB27IRM* (*E*. *coli*)	yes	36.9	approx. 2600	2: 1	approx. 870	1: 1

^1^ As determined in crude cell lysates.

^2^ Assays conducted in the optimal reaction buffer in the presence of SAM. Specific activities were estimated based on multiple series of the enzyme units titration.

^3^ The recombinant wt RM.TthHB27I exhibited very low activity in crude cell lysates. Any attempts to purify the thermozyme and estimate specific activity resulted in complete loss of activity.

Analysing the DNA cleavage and methylation reactions parameters of synthetic RM.TthHB27I, it is evident that they match those of the native wt thermozyme isolated from *T*. *thermophilus* (Fig 4 and Table 1). The data suggest that activities of wt and synthetic RM.TthHB27I are closely within 2-fold of each other with an estimated error of approximately 10%. However, there is one exception, apparently not due to the thermozymes itself—approximately 8-fold higher activity of native wt RM.TthHB27I REase in the absence of added exogenous cofactor SAM (Fig 4C). This difference almost disappears, when SAM is supplemented to the cleavage reaction (Fig 4E and 4F). Such results indicate a higher carry over of endogenous SAM from *T*. *thermophilus* cells than from *E*. *coli* cells. Besides this difference, both thermozymes do not cleave DNA to completion. Such behaviour was described for several Type IIS/IIG/IIC enzymes, including those belonging to the *Thermus*-family [39, 40]. These enzymes often require more than one recognition site on the substrate to cleave DNA optimally. Their need to bind to more than one site in order to cleave can make single-site substrates difficult to cut *in vitro*. For that reason, the second recognition site within the 1789 bp substrate may be cleaved inefficiently by RM.TthHB27I variants (Fig 4). Interestingly, the ‘stable partial digestion patterns’ observed for the investigated variants are not identical (Fig 4E and 4F). In case of the synthetic recombinant enzyme version, the distribution of the cleavage products points for slightly slower cleavage of the second recognition site (Fig 4D and 4F), when compared to the native enzyme. This may be caused by slightly higher specific activity of the MTase of the synthetic RM.TthHB27I variant (Fig 4B), changing the balance between both activities.

Despite the noticed difference in the enzymes kinetics, a high proportion of the protein to recognition sites in DNA is needed for both investigated protein variants. Maximum digestion (although still incomplete) requires, *in vitro*, a molar ratio of at least 2: 1 (!), thus such an amount we have used as the unit definition (Table 1). On the other hand, methylation can proceed to completion (thus we define the activity unit as complete methylation, while for the REase unit we use stable partial digestion definition), requiring, *in vitro*, a molar ratio of 1: 1. Both thermozyme variants exhibit very similar specific activities, which confirm their functional identities (Fig 4 and Table 1). It has been previously proposed for Mme-like enzymes, that they bind to their recognition sequences as monomers, but cleave DNA only after assembling into homodimers or homotetramers [41–43]. The *Thermus*-family enzymes have domain organization resembling MmeI-like enzymes [28, 42, 43]. It is highly probable that their mechanism of DNA cleavage is also similar. This hypothesis could explain the required high molar ratio of the protein to recognition sites. Overall, these results also shed some light on RM.TthHB27I (and other *Thermus*-family enzymes) as not quite behaving as ‘true’ catalysts. Besides confirming that SAM addition is required for efficient DNA cleavage by synthetic RM.TthHB27I, we have also shown that its analogue, SIN, is an efficient stimulator, indicating the allosteric nature of activation by this non-hydrolysable compound (S6 File). Other SAM analogues such as SAC or SAH, as well as ATP, have no effect on DNA cleavage (S6 File). The MTase activity is mildly stimulated by Ca^2+^ ions, in contrast to REase, which requires Mg^2+^ ions (S6 File). We believe that the latter may also activate MTase, but this hypothesis cannot be verified as the competing REase activity is turned on as well.

## Discussion

According to the literature, the ‘codon randomization’ strategy offers several advantages, potentially having the edge over the 'one aa—one codon' method. In the ‘codon randomization’ strategy, codons are assigned randomly according to a codon table and the employed synonymous codon proportions sets [10]. Using this approach, a large number of variants can be generated, which gives the opportunity to fine-tune the optimized nt sequence, for example by removing mRNA secondary structures [10]. A weighted mixture of the most frequent codons ensures the absence of possible exhaustion of the aminoacyl-tRNA pools, which could lead to aa misincorporation, slowing down or even terminating translation [8, 10]. Not only do these problems decrease the level of expression but also they may lead to the biosynthesis of a protein with reduced specific activity. This may cause a situation where correctly translated molecules are diluted by misfolded or partially folded molecules. Such defective protein molecules are not biologically active and may be formed due to altered translation kinetics. Mutations within an expressed gene, vector, or host genome, selected by a toxic effect during expression culture growth or accumulated misfolded protein (created by altered translation process), may be responsible for a significant decrease or complete loss of protein activity.

Additionally, overproduction of heterologous proteins can also lead to cellular damage, which has to be repaired. As a consequence, the recombinant host suffers metabolic stress, which leads to a decreased growth rate, cell fragility or lysis. We have observed such difficulties during the cloning/expression of the genes coding for the *Thermus*-family thermozymes or their engineered variants [24, 42, 43].

We hypothesize that the approach of combining codon optimization and partial hiding of ATG and RBS in the stem-loop structure may decrease the expression of potentially toxic gene to a moderate level, enabling the recombinant cells to cope with its product toxicity. However, in case of the RM.TthHB27I we have only tested a single sequence variant. Evaluation of this synthetic biology approach requires much precise data and a series of experiments with various variants/controls of the stem-loop structures, which is beyond the scope of this paper. Thus, even though the final synthetic RM.TthHB27I expression output was as high as assumed, the obtained result provides some preliminary support for the proposed hypothesis. Summarising, the general approach applicable to other genes expression problems includes the following steps:

1. Selection of an expression host and listing codon usage for highly expressed genes;
2. Construction of a custom codon usage table with eliminated codons of less than 13–16% relative occurrences and with increased percentage of synonymous AT-rich codons within a remaining pool of up to 50% for each coded aa;
3. Using a software with a back-translation feature, which allows for random distribution of synonymous codons within the initially designed synthetic gene. For the purpose of this work we used our custom software GeneOptimizer 1.1.2 (available upon request);
4. Manual or software-aided scanning of the designed gene sequence for the presence of stretches of the same consecutive codons. All identical codons located next to each other should be replaced, while maintaining synonymous codons proportion from the custom codon table;
5. Software-aided scanning for the presence of mRNA secondary structures and weakening any appearing structure by including AT-rich synonymous codons, while maintaining synonymous codons proportion from the custom codon table;
6. Scanning for the presence of codon context obstacles using dedicated software, such as [17], followed by the replacement of synonymous codons, while maintaining synonymous codons proportion from the custom codon table.

In this paper, we show a radical activity difference in the same protein expressed from different codon environments. This phenomenon could be explained by a few hypotheses. One can envision that the expressed recombinant wt RM.TthHB27I may be a mixture of two protein forms, where one is activity deficient. Such diversity of forms may be a result of translation from two alternative RBS and start codons. Another explanation could be a transient depletion of the available aminoacylo-tRNA, leading to aa misincorporation and production of an inactive protein. Such a depletion may be a result of differences in codon composition between *T*. *thermophilus* and *E*. *coli*. Additionally, translation kinetics of the wt *tthHB27IRM* gene may be slower in comparison to the synthetic version of the gene, thus affecting co-translational folding and generating an incorrect 3-dimensional structure of the translation product. Moreover, high GC content of mRNA and formation of secondary structures, stable at the mesophile growth temperature, may slow down transcription and, in turn, translation, thus causing the same effect.

Taken together, the *tthHB27IRM* and *taqIIRM* expression results from our previous publications [23, 24] and from the current work, form part of the ongoing discussion of the applicability of ‘codon randomization’ and 'one aa—one codon' methods. This is especially important, when targeting problematic genes. Aiming at maximum protein production, based just on the amount of the overexpressed polypeptide biosynthesis, may not always be the best strategy. Numerous subtle effects need to be considered, as was the case with the interesting RM.TthHB27I—an example of a large, multi-domain, highly toxic protein with an apparently flexible polypeptide structure/function, prone to conversion to non-active form. In this particular case, the mentioned effects turned out to be far more important for the final desired outcome–high production of protein. In our previous attempt, the recombinant wt RM.TthHB27I was of very poor biological activity [24]. This also indicates that various expression tasks will pose different challenges and can be solved in a number of alternative ways. For those reasons, the ‘codon randomization’ strategy gives a higher success chance, offering possibility for generation of large number of optimized sequence variants. This approach enables the balancing of other critical factors, as was probably the case for synthetic RM.TthHB27I thermozyme.

## Conclusions

1. A synthetic gene coding for the thermostable, bifunctional REase-MTase TthHB27I was cloned and overexpressed in *E*.*coli* under the control of a T7 promoter. This enzyme, with a previously unavailable DNA recognition specificity, has been purified and proved fully active.
2. The synthetic gene has optimized codons, codons context and mRNA secondary structures. The gene was designed using a modified ‘codon randomization’ approach. This approach included moderate biasing towards sub-optimal, low GC content codons in *E*. *coli*, eliminating codons of less than 13–16% relative occurrences in *E*. *coli* and the regulatory stem-loop circuit, partially submerging the RBS and start codon in the 5’-end ds mRNA segment to control DNA-destructive *in vivo* action of the synthetic RM.TthHB27I.
3. Our previously reported cloning and overexpression of the wt *tthHB27IRM* gene [24] yielded a high production of the RM.TthHB27I polypeptide, but with a very low enzymatic activity, which was inactivated rapidly. We prove that the use of a modified ‘codon randomization’ method for constructing the synthetic *tthHB27IRM* gene, resulted in restoring the activity and stability of the synthetic RM.TthHB27I to the level of the native wt RM.TthHB27I from *T*. *thermophilus*. It is extremely unusual that soluble recombinant protein variants, encoded by different gene variants and produced at similar levels, exhibit such a dramatic difference in activity. We hypothesize that this may be due to differences in co-translation folding kinetics.
4. The modified ‘codon randomization’ strategy, that we used for the toxic and problematic REase-MTase-coding gene, could also be suited for solving problems with other thermostable enzymes production.

## Supporting information

S1 File Methods. (DOCX)

S2 File *tthHB27IRM* genes sequences and protein domains. The DNA sequence of the recombinant wt-*tthHB27IRM* gene is shown in blue. The DNA sequence of the synthetic *tthHB27IRM* gene is indicated in black bold letters and the changed bases are marked in red. The predicted aa sequence of the 127.7 kDa recombinant wt and synthetic RM.TthHB27I protein is shown in capital letters. The crucial aas of the catalytic centres are dark red, bold and underlined. The functional RM.TthHB27I domains are indicated as follows: REase domain in blue, helical domain in light green, MTase domain in dark green and the potential TRD region in brown. Numbering of nt of *tthHB27IRM* gene variants and polypeptide aa starts as '1' with the beginning (ATG) of the synthetic *tthHB27IRM* ORF, which corresponds to the first ATG start codon of the recombinant wt *tthHB27IRM*.
(PDF)

S3 File Mascot Search Results 366 wt RM.TthHB27I. (PDF)

S4 File Mascot Search Results 366 rec RM.TthHB27I. (PDF)

S5 File Mascot Search Results 366 syn RM.TthHB27I. (PDF)

S6 File SAM, SAM analogues and divalent cations effects. (A) Effect of cofactor or its analogues on synthetic RM.TthHB27I REase activity. 0.5 **μ**g of 1789 bp PCR DNA substrate was digested with 2 units of synthetic RM.TthHB27I in REase buffer supplemented with 50 **μ**M of the selected effector at 65°C. Lane M1, 100 bp Plus DNA Ladder; lane M2, GeneRuler 1 kb DNA Ladder, lane K, untreated PCR fragment; lane 1, PCR fragment digested with synthetic RM.TthHB27I, no allosteric effector; lane 2, as in lane 1, supplemented with SAM; lane 3, supplemented with SIN; lane 4, supplemented with SAC; lane 5, supplemented with SAH; lane 6, supplemented with ATP. (B) MTase activity assay. Lane M, GeneRuler 1 kb DNA Ladder (Thermo Fisher Scientific/Fermentas); lane K, untreated 1789 bp PCR DNA substrate; lane 1, incubation with synthetic RM.TthHB27I in the MTase base buffer supplemented with 100 **μ**M SAM and 6 mM Mg^2+^; lane 2, as in lane 1, supplemented with 6 mM Ca^2+^ instead of Mg^2+^ ions; lane 3, supplemented with 3 mM EDTA instead of Mg^2+^ ions. The complete digestion pattern includes restriction fragments of 872, 602 and 311 bp (bold). Fragments indicated in italics (1476 and 915 bp) are a result of incomplete digestion.
(TIF)

S1 Table Codon distribution within the *tthHB27IRM* genes. (DOCX)

S2 Table Codon usage data. (DOCX)
