# Supplementary material for: Thermostable proteins bioprocesses: The activity of restriction endonuclease-methyltransferase from Thermus thermophilus (RM.TthHB27I) cloned in Escherichia coli is critically affected by the codon composition of the synthetic gene
Source: PLoS One. 2017 Oct 17;12(10):e0186633. doi: 10.1371/journal.pone.0186633 (PMC5645126; doi:10.1371/journal.pone.0186633)
Supplement: S1 File — (DOCX) [file pone.0186633.s001.docx]

Supporting information

**PCR amplification of the synthetic *tthHB27IRM* gene**

The synthetic *tthHB27IRM* gene was amplified from the synthetic DNA using the PCR primer pair: FsynTth 5’-CC**GGTCTC**ACatgCTGTCTCTGC-3’ (beginning of the gene underlined) and RsynTth 5’-CC**GTCGA**ctaCCACGCAGCTC-3’ (3’ terminal portion of the gene underlined). The forward primer introduced the BsaI REase site and instead of GTG codon, the ATG start was used, as in our previous report, describing wt recombinant RM.TthHB27I [24]. The reverse primer introduced a SalI REase site directly after the TAG stop codon. Both REase sites are bold. The start and the complement of the stop codons are in small letters. The PCR reactions were performed in 100 μl volumes containing: 1× Marathon PCR Buffer, 0.4 mM of each dNTP, 0.5 μM of each primer, 100 ng of synthetic *tthHB27IRM* gene DNA, 3% DMSO and 1 unit of Marathon DNA Polymerase. The PCR cycling profile was as follows: 97^o^C for 4 min, 89^o^C for 20 sec (addition of DNA polymerase), 95^o^C for 30 sec, 62^o^C for 30 sec, 68^o^C for 4 min (30 cycles) and final extension at 68^o^C for 5 min. The amplification product was purified and digested with BsaI and SalI. Plasmid pET21d(+) was cleaved with NcoI and SalI. The digested DNAs were purified, ligated and transformed into *E. coli* TOP10.

The resulting clones carrying the pET21d(+)-synthetic *tthHB27IRM* plasmid were analysed by SalI and ScaI digestion as well as multiple sequencing runs of selected clones. The cloning of wt *tthHB27IRM* gene we have described previously [24].

**Gel electrophoresis and protein concentration determination**

For DNA analysis, 1.3% agarose gels were prepared in TBE buffer [26]. The gels were visualized after staining with ethidium bromide using a 312 nm UV transilluminator and photographed with a photographic filter. For protein analysis, SDS-PAGE electrophoresis was conducted in 7.5% polyacrylamide gels [26]. The gels were visualized after staining with Coomassie Brilliant Blue R-250. For quantifications of selected protein bands series of photographs were taken at various exposure conditions and the software UN-SCAN IT GEL for Windows 6.1 data software (v. 6.1, Gel Analysing and Graph Digitizing Software, Silk Scientific Corporation, Orem, Utah, USA) was used, relating the obtained values to calibration gels, containing various amounts of BSA.

**Mass spectrometry analysis of RM.TthHB27I variants**

The purified, native wt RM.TthHB27I, recombinant wt RM.TthHB27I and synthetic RM.TthHB27I (the protein expressed from the synthetic *tthHB27IRM* gene is not a synthetic macromolecule, but for simplicity we further designate it as ‘synthetic’) were subjected to Mass Spectrometry using Thermo Orbitrap Elite equipment linked with Thermo EASY-nLC 1000. This machine offers high resolution and sensitivity. Protein samples were treated with semiTrypsin, which cuts at the C-terminal side of Lys-Arg, unless the next residue is Pro. Cleavage is semi-specific, as a peptide can be non-specific at one terminus only. Fixed modifications used: Carbamidomethyl (C), variable modifications: Oxidation (M).
