# Supplementary material for: Thermostable proteins bioprocesses: The activity of restriction endonuclease-methyltransferase from Thermus thermophilus (RM.TthHB27I) cloned in Escherichia coli is critically affected by the codon composition of the synthetic gene
Source: PLoS One. 2017 Oct 17;12(10):e0186633. doi: 10.1371/journal.pone.0186633 (PMC5645126; doi:10.1371/journal.pone.0186633)
Supplement: S2 File — The DNA sequence of the recombinant wt-tthHB27IRM gene is shown in blue. The DNA sequence of the synthetic tthHB27IRM gene is indicated in black bold letters and the changed bases are marked in red. The predicted aa sequence of the 127.7 kDa recombinant wt and synthetic RM.TthHB27I protein is shown in capital letters. The crucial aas of the catalytic centres are dark red, bold and underlined. The functional RM.TthHB27I domains are indicated as follows: REase domain in blue, helical domain in light green, MTase domain in dark green and the potential TRD region in brown. Numbering of nt of tthHB27IRM gene variants and polypeptide aa starts as '1' with the beginning (ATG) of the synthetic tthHB27IRM ORF, which corresponds to the first ATG start codon of the recombinant wt tthHB27IRM. (PDF) [file pone.0186633.s002.pdf]

PD-(D/E)XK domain

ATG CTG TCT CTG CTG ACC GGT GGT GTA TTT CGT CGC GTT AAA CTG ATG AAC TGG ATC GAC CTG TAC ACC CAC CTG AAA CAG GAA GTT CCG TGG TTT TTC AAC AGC GTT CGT CTG GCA GCG 120 syn-*tthHB27IRM*
ATG CTT AGC CTT CTT ACA GGG GGT GTG TTT AGG AGG GTT AAA CTC ATG AAC TGG ATC GAT CTT TAC ACC CAT CTA AAA CAA GAG GTC CCT TGG TTT TTT AAT TCC GTC CGT CTC GCA GCC 120 wt-*tthHB27IRM*
M L S L L T G G V F R R V K L M N W I D L Y T H L K Q E V P W F F N S V R L A A 40 RM.TthHB27I

AGC CAG GCT CAC AAC GAG GCT GAA TTT GAA TCT CGT ATC AAC AAT GCA ATC GAA CGT CTG GCA CAG AAG CTG GGC GTT CAG CTG CTG TTC CGT GAA CAG TAC ACC CTG GCA ACC GGT CGT 240 syn-*tthHB27IRM*
AGC CAA GCC CAT AAC GAG GCC GAG TTT GAG AGT CGG ATA AAC AAT GCA ATT GAG CGC TTG GCT CAG AAG TTG GGT GTT CAG CTG CTT TTC CGG GAA CAA TAT ACG CTG GCC ACT GGC CGC 240 wt-*tthHB27IRM*
S Q A H N E A E F E S R I N N A I E R L A Q K L G V Q L L F R E Q Y T L A T G R 80 RM.TthHB27I

PD-(D/E)XK: catalytic center of REase

GCA GAC GCG GTA TAC AAC CGT CTG GTA ATC GAA TAT GAA CCA CCG GGT TCC CTG CGT CCG AAC CTG AAA CAC TCT CAC ACT CAG CAC GCA GTT CGT CAG GTA ATG AAC TAC ATC GAG GAA 360 syn-*tthHB27IRM*
GCT GAT GCT GTG TAC AAC CGT CTG GTG ATA GAA TAC GAG CCA CCC GGT TCT TTG CGG CCA AAT TTG AAA CAC AGC CAC ACT CAG CAT GCG GTG CGG CAG GTC ATG AAC TAC ATT GAG GAG 360 wt-*tthHB27IRM*
A **D** A V Y N R L V I **E** **Y** **E** P P G S L R P N L K H S H T Q H A V R Q V M N Y I E E 120 RM.TthHB27I

CTG TCT CGT GCT GAG CGT CAT GAC CGT GAT CGT CTG CTC GGC GTA GTT TTC GAT GGT CAC TAC TTC ATC TTC GTA CGC TAC CAC GAA GGT CAC TGG ATC GTT GAA GAG CCG CTC GAA GTA 480 syn-*tthHB27IRM*
TTA TCC AGA GCG GAA AGG CAT GAC CGC GAC CGC CTG CTG GGG GTC GTC TTC GAC GGC CAC TAC TTC ATC TTT GTC CGC TAC CAT GAG GGG CAC TGG ATC GTA GAA GAG CCC CTG GAG GTG 480 wt-*tthHB27IRM*
L S R A E R H D R D R L L G V V F D G H Y F I F V R Y H E G H W I V E E P L E V 160 RM.TthHB27I

helical domain

AAC CCG GCA TCT TGT GAA CGT TTC CTG CGT TCT CTG TTC TCC CTG TCT TCT GGT CGC GCG CTG ATC CCG GAA AAC CTC GTT GAA GAC TTT GGC TCC CAA AAT GAT CTG TCC CGT CAG GCA 600 syn-*tthHB27IRM*
AAT CCG GCG TCG TGT GAG CGC TTC CTG CGT TCT CTC TTC TCC CTT TCT TCG GGC CGG GCG CTG ATT CCC GAG AAC CTG GTG GAG GAC TTC GGG AGC CAG AAC GAC CTC AGC CGC CAG GCC 600 wt-*tthHB27IRM*
N P A S C E R F L R S L F S L S S G R A L I P E N L V E D F G S Q N D L S R Q A 200 RM.TthHB27I

ACT CGC GCT CTC TAT CAC GCT CTG CAG GGT CAC ACT TCC GAC CTG ACC GCT CGT CTG TTC GTT CAG TGG CAA ATC TTC TTT GGT GAA ACT GCA GGT GCT GAT GCG GCT GGC GGT GAA CTG 720 syn-*tthHB27IRM*
ACC CGT GCC CTC TAC CAC GCG CTG CAG GGT CAT ACC AGT GAT CTG ACC GCC CGC CTC TTT GTC CAG TGG CAA ATC TTC TTC GGC GAG ACG GCC GGT GCC GAT GCG GCT GGA GGC GAA CTA 720 wt-*tthHB27IRM*
T R A L Y H A L Q G H T S D L T A R L F V Q W Q I F F G E T A G A D A A G G E L 240 RM.TthHB27I

AAA CAC AAG AGC GAG CTG CTG GCT TTT GCG CGT GGC ATG GGC CTG CGC GGC TCT CGT ATC GAC ATG CCG CGT TTC CTG TTT GCA CTG CAT ACC TAC TTC AGC TTC CTG GTT AAA AAC ATT 840 syn-*tthHB27IRM*
AAG CAC AAG AGT GAA CTG CTT GCC TTT GCC CGC GGC ATG GGG CTG CGG GGC AGC CGG ATA GAC ATG CCC CGC TTC CTC TTT GCC CTG CAC ACG TAC TTC TCC TTC CTG GTC AAA AAC ATC 840 wt-*tthHB27IRM*
K H K S E L L A F A R G M G L R G S R I D M P R F L F A L H T Y F S F L V K N I 280 RM.TthHB27I

GCT CGT CTG GTT CTG CAG GCG TAC GCA GGT GGC GGT CTG GGT ACT ACC CCG CTG ACC ACC ATC GCG AAC CTG GAA GGT GAA GCT CTG CGT CGC GAA CTG CAG AAC CTG GAG TCT GGT GGC 960 syn-*tthHB27IRM*
GCC CGC CTG GTG CTC CAG GCC TAT GCG GGT GGC GGG CTG GGC ACC ACG CCC CTG ACC ACC ATC GCC AAC CTG GAA GGC GAG GCC CTG CGC CGG GAA CTG CAA AAC CTG GAA AGC GGC GGA 960 wt-*tthHB27IRM*
A R L V L Q A Y A G G G L G T T P L T T I A N L E G E A L R R E L Q N L E S G G 320 RM.TthHB27I

CTC TTC CGT ACT CTG GGC CTG AAA AAC CTG CTG GAA GGC GAC TTC TTC GCG TGG TAT CTG GAC GCG TGG AAC CCG GAA GTT GAG GAA GCT CTG CGT CAG GTT CTC GCA CGT CTC GCA GAG 1080 syn-*tthHB27IRM*
CTT TTC CGT ACC CTG GGC CTA AAG AAC CTG CTG GAG GGT GAC TTC TTC GCC TGG TAC CTG GAC GCC TGG AAC CCG GAA GTG GAA GAA GCC CTG CGC CAG GTG CTG GCC CGC CTG GCC GAG 1080 wt-*tthHB27IRM*
L F R T L G L K N L L E G D F F A W Y L D A W N P E V E E A L R Q V L A R L A E 360 RM.TthHB27I

RFM domain

TAC AAC CCG GCT ACT GTT CAG GAC GAT CCA CAC TCT GCA CGT GAT CTG CTG AAA AAG CTG TAC CAC TAC CTG CTG CCA CGC GAC ATC CGC CAC GAT CTG GGC GAA TTC TAC ACC CCG GAC 1200 syn-*tthHB27IRM*
TAC AAC CCG GCC ACC GTG CAG GAC GAC CCC CAC AGC GCC CGC GAC CTG CTG AAA AAG CTC TAC CAC TAC CTC CTG CCG CGG GAC ATC CGC CAC GAC CTG GGC GAG TTC TAC ACC CCC GAC 1200 wt-*tthHB27IRM*
Y N P A T V Q D D P H S A R D L L K K L Y H Y L L P R D I R H D L G E F Y T P D 400 RM.TthHB27I

Motif I: S-adenosylmethionine-binding site

TGG CTG GCG GAA CGC CTG CTG AAT CAG CTG GGT GAA CCG TGG TTC ATC ATG CCG CCA GGC AAC CAC CCG CCA CGT GGC CTG CCG GAT AAG CGT CTC CTG GAC CCG GCT TGC GGT TCC GGT 1320 syn-*tthHB27IRM*

TGG CTG GCC GAG CGT CTG CTC AAC CAG CTG GGT GAA CCC TGG TTC ATC ATG CCC CCG GGG AAC CAC CCG CCC AGG GGC TTG CCC GAC AAG CGC CTG CTG GAC CCG GCC TGC GGC TCC GGC 1320 wt-*tthHB27IRM*

W L A E R L L N Q L G E P W F I M P P G N H P P R G L P D K R L L D P A C G S G 440 RM.TthHB27I

ACC TTC CTG GTT CTG GCA ATC CGT GCA CTG AAG GTT AAC TGC TTT CTG GCG GGT TTC TCT GAA GCG GAC ACC CTG GAA GTT ATC CTG AAC TCC GTT GTT GGC ATC GAC CTG AAC CCG CTC 1140 syn-*tthHB27IRM*

ACC TTC CTG GTG CTG GCC ATC CGC GCC CTC AAG GTC AAC TGC TTC CTG GCT GGC TTC TCC GAG GCT GAC ACC CTG GAG GTT ATC CTG AAC AGC GTG GTG GGC ATT GAC CTC AAC CCC TTG 1140 wt-*tthHB27IRM*

T F L V L A I R A L K V N C F L A G F S E A D T L E V I L N S V V G I D L N P L 480 RM.TthHB27I

GCG GTA ACT GCA GCG CGC GTT AAC TAC CTC CTG GCG ATT GCT GAT CTC CTG CCG TAT CGT CGC CGT GAA GTA GAA ATC CCG GTT TAC CTG GCT GAC TCC ATC CTG ACC CCG GCA CGT GGT 1560 syn-*tthHB27IRM*

GCT GTG ACC GCA GCC CGG GTC AAC TAC CTG CTG GCC ATC GCC GAC CTG CTC CCT TAC CGC CGC CGG GAG GTG GAA ATT CCG GTC TAT CTC GCC GAC AGC ATA CTT ACG CCG GCC CGC GGG 1560 wt-*tthHB27IRM*

A V T A A R V N Y L L A I A D L L P Y R R R E V E I P V Y L A D S I L T P A R G 520 RM.TthHB27I

GAA GGT CTG TTT GCG CAG AAC CGC CGT ATC CTG GAG ACT GCT GTT GGC CCG CTG CCG GTT CCG GAG GTA ATC AAC AGC CGT GCA AAA ATG GAA CGT CTG ACT GAC CTG CTG GAG GAA TAC 1680 syn-*tthHB27IRM*

GAA GGG CTC TTC GCC CAG AAC CGC CGC ATC CTG GAG ACC GCG GTC GGC CCC CTG CCC GTG CCC GAG GTG ATT AAC AGC CGC GCT AAG ATG GAA CGG CTC ACC GAC CTG CTT GAA GAG TAC 1680 wt-*tthHB27IRM*

E G L F A Q N R R I L E T A V G P L P V P E V I N S R A K M E R L T D L L E E Y 560 RM.TthHB27I

GTT CGT GGC GAC TTC AGC ACC GAG GCG TTC CTG GCT CGT GCA AAA AAG GAA ATT CCA GAC CTG GCA GAC GCT CTC CAC GCA GAT GAA GTT CTG ACC GAA CTG TAC GAA CGT CTG CGT GAC 1800 syn-*tthHB27IRM*

GTC CGC GGG GAT TTC TCC ACC GAG GCC TTC CTC GCC CGG GCC AAA AAG GAA ATC CCC GAC CTG GCC GAT GCC CTC CAT GCC GAC GAA GTG CTC ACC GAA CTC TAC GAG AGG CTG CGC GAC 1800 wt-*tthHB27IRM*

V R G D F S T E A F L A R A K K E I P D L A D A L H A D E V L T E L Y E R L R D 600 RM.TthHB27I

Motif IV: catalytic center of MTase

CTG CAC CGT CAG GGT CTG GAC GGC ATC TGG GCG CGC GTT CTG AAA AAT GCG TTC ATG CCA CTG TTC CTG GAG CCG TTC GAC TAC GTT GTT GGC AAC CCA CCG TGG ATC AAC TGG GAA TCC 1920 syn-*tthHB27IRM*

CTC CAC CGC CAG GGG CTA GAT GGC ATC TGG GCC CGG GTG CTC AAG AAC GCT TTC ATG CCC CTC TTC CTG GAA CCC TTT GAC TAC GTG GTG GGC AAT CCG CCC TGG ATC AAC TGG GAA AGC 1920 wt-*tthHB27IRM*

L H R Q G L D G I W A R V L K N A F M P L F L E P F D Y V V G N P P W I N W E S 640 RM.TthHB27I

CTG CCG CAG GCT TAC CGT GAA CAG ACC GCT GAA CTG TGG ACC TGC TAC GGT CTG TTT GTT CAC TCC GGT ATG GAC ACC ATT CTG GGT AAA GGT AAG AAA GAT GCT TCT ACT CTC ATG ACC 2040 syn-*tthHB27IRM*

TTG CCC CAG GCC TAC CGG GAG CAA ACG GCC GAG TTA TGG ACA TGC TAC GGC CTC TTC GTC CAT TCC GGC ATG GAT ACC ATC CTG GGC AAG GGC AAA AAG GAC GCC TCC ACC CTG ATG ACC 2040 wt-*tthHB27IRM*

L P Q A Y R E Q T A E L W T C Y G L F V H S G M D T I L G K G K K D A S T L M T 680 RM.TthHB27I

TAT GCG GTT GCA GAC CGT TTC CTG AAA GAA GGC GGT AAA CTG GGT TTC CTG ATC ACC CAG TCT GTA TGG AAA ACT GGC GCT GGT CAG GGT TTT CGC CGT TTT CGC ATC GGC GAA AAC GGT 2160 syn-*tthHB27IRM*

TAC GCC GTG GCC GAC CGC TTC TTG AAA GAG GGC GGC AAA CTG GGC TTC CTC ATC ACC CAG AGC GTC TGG AAA ACT GGG GCT GGG CAG GGC TTC CGC CGT TTC CGT ATC GGA GAA AAC GGC 2160 wt-*tthHB27IRM*

Y A V A D R F L K E G G K L G F L I T Q S V W K T G A G Q G F R R F R I G E N G 720 RM.TthHB27I

TRD

CCG CAT CTG CGT GTT CTG CAT GTT GAC GAT CTG TCT TCC CTG CAG GTT TTC GAA GGT GCT TCC ACC CGC ACC TCC GCA TTC GTT CTG CAG AAA GGT CGT CCG ACC CGT TAC CCA GTA CCG 2280 syn-*tthHB27IRM*

CCC CAT TTG CGC GTG CTA CAC GTG GAC GAC CTC TCC AGC CTG CAA GTC TTT GAA GGA GCC AGC ACA CGC ACC AGC GCC TTC GTC CTG CAG AAG GGC CGG CCC ACC CGC TAC CCG GTG CCC 2280 wt-*tthHB27IRM*

P H L R V L H V D D L S S L Q V F E G A S T R T S A F V L Q K G R P T R Y P V P 760 RM.TthHB27I

TAC ACT TAC TGG AAA AAG ACC ACC AAA GGT GAG GGT CTG GAT TAC GAC TCT ACT CTG GGC GAA GTT ATG GAA CAG ACC AAA CGT CTC CGC TTC CAC GCA GTA CCG GTT GAC CCG GAC GAT 2400 syn-*tthHB27IRM*

TAC ACT TAC TGG AAG AAG ACG ACC AAA GGC GAG GGG CTG GAC TAC GAC AGC ACC CTG GGC GAG GTG ATG GAA CAG ACC AAA CGT CTT CGG TTC CAC GCC GTG CCG GTG GAC CCG GAC GAC 2400 wt-*tthHB27IRM*

Y T Y W K K T T K G E G L D Y D S T L G E V M E Q T K R L R F H A V P V D P D D 800 RM.TthHB27I

CTG ACC TCT CCG TGG CTG ACT GCA CGC CGT CGC GCA CTG TAC GCT GTA CGT AAA GTT CTG GGC ACT TCC GAA TAT CGC GCG TAT GAA GGC GCG AAC TCC GGT GGC GCG AAT GGT ATC TAC 2520 syn-*tthHB27IRM*

CTC ACC AGC CCC TGG CTC ACC GCC CGC CGC AGG GCC CTG TAC GCC GTG CGC AAG GTG CTG GGG ACG TCG GAG TAC CGG GCG TAC GAA GGA GCC AAC AGT GGA GGA GCC AAC GGC ATC TAC 2520 wt-*tthHB27IRM*

L T S P W L T A R R R A L Y A V R K V L G T S E Y R A Y E G A N S G G A N G I Y 840 RM.TthHB27I

TGG CTG GAG ATC CTG GCT GAA CGT CCG GAC GGC CTG GTT GTA GTT CGT AAC GTA ACC GAA GGC GCT AAG CGC GAA GTT GAA GGC ATT ACT ACT GAA CTG GAA CCG GAC CTG CTG TAC CCG 2640 syn-*tthHB27IRM*

TGG CTG GAA ATC CTG GCC GAG CGA CCG GAC GGG CTG GTG GTG GTG CGC AAT GTG ACT GAG GGG GCT AAA CGG GAG GTG GAG GGC ATT ACC ACC GAA CTG GAG CCC GAC CTG CTC TAC CCC 2640 wt-*tthHB27IRM*

W L E I L A E R P D G L V V V R N V T E G A K R E V E G I T T E L E P D L L Y P 880 RM.TthHB27I

CTG CTG CGC GGT CGT GAT GTA CGC CGT TGG TAC GCT CAA CCG TCC CTG CAC ATC CTG ATG GTA CAG GAT CCG AAG ACC CGT CGT GGT ATC GAT GAA CAG GTT CTC CAA AAA CGT TAT CCA 2760 syn-*tthHB27IRM*

CTG CTG CGC GGC CGG GAT GTG CGC CGC TGG TAT GCA CAA CCA TCT TTG CAC ATC CTC ATG GTG CAG GAC CCC AAG ACG CGG CGG GGC ATA GAC GAG CAG GTG CTC CAG AAG CGC TAC CCC 2760 wt-*tthHB27IRM*

L L R G R D V R R W Y A Q P S L H I L M V Q D P K T R R G I D E Q V L Q K R Y P 920 RM.TthHB27I

AAA ACT TGG GCA TAC CTG AAA CGT TTC GAG GCT GTT CTG CGC GAG CGT TCT GGT TTC CGT CGC TAC TTC ACC CGC AAA GAC CGT AAC GGT CGC ATG GTT GAA ACT GGT CCG TTT TAC TCT 2880 syn-*tthHB27IRM*

AAG ACC TGG GCC TAC CTC AAG CGC TTT GAG GCG GTG CTG CGG GAG CGT TCC GGC TTC AGG CGC TAC TTT ACC CGC AAG GAC AGG AAC GGC CGC ATG GTG GAA ACC GGC CCC TTC TAC TCT 2880 wt-*tthHB27IRM*

K T W A Y L K R F E A V L R E R S G F R R Y F T R K D R N G R M V E T G P F Y S 960 RM.TthHB27I

ATG TTC AAC GTA GGT GAC TAT ACC TTC GCA CCG TGG AAA GTA GTT TGG CGT TAT GTA GCT TCC GAC TTC ATC GTT GCT GTT GTA GGT CCG GCG TCC GAT GAA AAG CCG GTT GTA CCG AAC 3000 syn-*tthHB27IRM*

ATG TTT AAC GTC GGC GAC TAC ACC TTC GCG CCG TGG AAG GTG GTG TGG CGA TAC GTG GCT TCG GAT TTT ATT GTT GCT GTA GTA GGT CCT GCT TCA GAT GAG AAG CCC GTT GTT CCT AAC 3000 wt-*tthHB27IRM*

M F N V G D Y T F A P W K V V W R Y V A S D F I V A V V G P A S D E K P V V P N 1000 RM.TthHB27I

GAA AAA CTG ATG CTG GTA CCG GTT GAA GAC GAT AAC GAA GCA TTC TAC CTG TGT GGC GTT CTG AAC TCT TCC CCG ATC CGT TTC GCA GTT CAG TCC TTC TTT GTT CAG ACC CAG ATC GCT 3120 syn-*tthHB27IRM*

GAA AAG CTT ATG TTA GTG CCT GTT GAA GAC GAT AAT GAG GCT TTC TAC TTG TGT GGG GTT CTG AAC TCT TCT CCA ATC CGT TTT GCG GTC CAA AGT TTC TTT GTC CAA ACA CAA ATT GCC 3120 wt-*tthHB27IRM*

E K L M L V P V E D D N E A F Y L C G V L N S S P I R F A V Q S F F V Q T Q I A 1040 RM.TthHB27I

CCG CAC GTA CTG CAG AAA CTG TGT ATT CCA CGC TAC GAA CCG AAC ACC GAT CAC CAA AAC CGT ATC GCA CAC CTG TCT CGC CGT GCT CAC GAA CTG GCA CCA GCA GCT TAC AAC GGT GAC 3240 syn-*tthHB27IRM*

CCT CAC GTG CTT CAA AAA CTT TGC ATT CCC AGA TAT GAA CCG AAC ACT GAC CAT CAA AAT CGC ATC GCC CAC CTC TCC CGC CGC GCC CAC GAG CTG GCC CCG GCG GCC TAC AAT GGG GAC 3240 wt-*tthHB27IRM*

P H V L Q K L C I P R Y E P N T D H Q N R I A H L S R R A H E L A P A A Y N G D 1080 RM.TthHB27I

AAA GCG GCA CGT GCG GAA CTG CGT CGT GTT GAA GAG GAA ATC GAC CGT GCG GCA GCT CAG CTG TGG GGT CTG ACT GAG GAA GAG CTG GCA GAA ATC CGC CGT TCC CTG GAA GAG CTG CGT 3318 syn-*tthHB27IRM*

AAA GCG GCC CGG GCC GAA CTG CGG CGG GTG GAA GAG GAG ATT GAC CGG GCC GCG GCC CAA CTC TGG GGC CTG ACG GAG GAG GAA CTG GCC GAG ATT CGG CGG AGT TTG GAG GAG TTG CGG 3318 wt-*tthHB27IRM*

K A A R A E L R R V E E E I D R A A A Q L W G L T E E E L A E I R R S L E E L R 1105 RM.TthHB27I

GGT TAG 3321 syn-*tthHB27IRM*

GGG TAG 3321 wt-*tthHB27IRM*

G * 1106 RM.TthHB27I
