# Supplementary material for: Thermostable proteins bioprocesses: The activity of restriction endonuclease-methyltransferase from Thermus thermophilus (RM.TthHB27I) cloned in Escherichia coli is critically affected by the codon composition of the synthetic gene
Source: PLoS One. 2017 Oct 17;12(10):e0186633. doi: 10.1371/journal.pone.0186633 (PMC5645126; doi:10.1371/journal.pone.0186633)
Supplement: S3 File — (PDF) [file pone.0186633.s003.pdf]

MASCOT Search Results

Protein View: 366

RM.TthHB27I [agafab 20160615]

**Database:**	small-www
**Score:**	8308
**Nominal mass (M_r):**	127974
**Calculated pI:**	6.77

Sequence similarity is available as **an NCBI BLAST search of 366 against nr**.

Search parameters

**MS data file:**	\\212.87.29.243\Dane\USERS\Agata\tmp\60614969kref_wt_TthHB27I.mgf
**Enzyme:**	semiTrypsin: cuts C-term side of KR unless next residue is P.<br>Cleavage is semi-specific. (Peptide can be non-specific at one terminus only.)
**Fixed modifications:**	**Carbamidomethyl (C)**
**Variable modifications:**	**Oxidation (M)**

Protein sequence coverage: 59%

Matched peptides shown in ***bold red***.

1 MLSLLTGGVF RRVKL**MNWID LYTHLKQEVP WFFNSVRLAA SQAHNEAEFE**
51 **SRINNAIER**L AQK**LGVQLLF REQYTLATGR ADAVYNRLVI EYEPPGSLRP**
101 **NLK**HSHTQHA VR**QVMNYIEE LSR**AERHDRD RLLGVVFDGH YFIFVRYHEG
151 HWIVEEPLEV NPASCERFLR **SLFSLSSGRA LIPENLVEDF GSQNDLSR**QA
201 TR**ALYHALQG HTSDLTAR**LF VQWQ**IFFGET AGADAAGGEL KHKSELLAFA**
251 **R**GMGLRGSRI DMPRFLFALH TYFSFLVKNI ARLVLQAYAG GGLGTTPL**TT**
301 **IANLEGEALR** R**ELQNLESGG LFR**TLGLKNL LEGDFFAWYL DAWNPEVEEA
351 LRQVLAR**LAE YNPATVQDDP HSAR**DLLKK**L YHYLLPR**DIR **HDLGEFYTPD**
401 **WLAER**LLNQL GEPWFIMPPG NHPPRGLPDK R**LLDPACGSG TFLVLAIR**AL
451 KVNCFLAGFS EADTLEVILN **SVVGIDLNPL AVTAAR**VNYL LA**IADLLPYR**
501 R**REVEIPVYL ADSILTPARG EGLFAQNRRI LETAVGPLPV PEVINSR**AKM
551 ER**LTDLLEEY VRGDFSTEAF LAR**AKKEIPD LADALHADEV LTELYERLRD
601 LHR**QGLDGIW AR**VLKNAFMP LFLEPFDYVV GNPPWINW**ES LPQAYR**EQTA
651 ELWTCYGLFV H**SGMDTILGK** GK**KDASTLMT YAVADR**FLKE GGK**LGFLITQ**
701 **SVWKTGAGQG FR**RFR**IGENG PHLRVLHVDD LSSLQVFEGA STRTSAFVLQ**
751 **KG**RPTR**YPVP YTYWK**K**TTKG EGLDYDSTLG EVMEQTKR**LR **FHAVPVDPDD**
801 **LTSPWLTAR**R **RALYAVRKVL GTSEYR**AYEG ANSGGANGIY WLEILAERPD
851 GLVVVR**NVTE GAK**REVEGIT TELEPDLLYP LLRGRDVRR**W YAQPSLHILM**
901 **VQDPK**TR**RGI DEQVLQKR**YP K**TWAYLKRFE AVLR**ERSGFR RYFTRKDRNG
951 R**MVETGPFYS MFNVGDYTFA PWK**VVWR**YVA SDFIVAVVGP ASDEKPVVPN**
1001 **EK**LMLVPVED DNEAFYLCGV LNSSPIR**FAV QSFFVQTQIA PHVLQK**LCIP
1051 R**YEPNTDHQN** RIAHLSR**RAH ELAPAAYNGD K**AARAELR**RV EEEIDRAAAQ**
1101 **LWGLTEEELA EIRRSLEELR G**

Unformatted sequence string: **1121 residues** (for pasting into other applications).

Sort peptides by ◉ Residue Number ○ Increasing Mass ○ Decreasing Mass

Query	Start – End	Observed	Mr(expt)	Mr(calc)	ppm	M	Score	Expect	Rank	U	Peptide
1605	16 – 26	725.3620	1448.7094	1448.7122	-1.92	0	49	2.5e-05	1	U	L.MNWIDLYTHLK.Q + Oxidation (M)
1606	16 – 26	483.9110	1448.7111	1448.7122	-0.76	0	67	4.8e-07	1	U	L.MNWIDLYTHLK.Q + Oxidation (M)
1504	27 – 37	704.8541	1407.6936	1407.6935	0.016	0	79	1.5e-08	1	U	K.QEVPWFFNSVR.L
72	32 – 37	385.2033	768.3921	768.3919	0.24	0	32	0.00069	1	U	W.FFNSVR.L
575	38 – 47	506.2469	1010.4792	1010.4781	1.08	0	34	0.00039	1	U	R.LAASQAHNEA.E
1769	38 – 52	553.9274	1658.7605	1658.7648	-2.61	0	88	1.5e-09	1	U	R.LAASQAHNEAEFESR.I
1770	38 – 52	553.9275	1658.7607	1658.7648	-2.48	0	64	4.3e-07	1	U	R.LAASQAHNEAEFESR.I
1771	38 – 52	830.3886	1658.7626	1658.7648	-1.33	0	133	5e-14	1	U	R.LAASQAHNEAEFESR.I
1775	38 – 52	553.9296	1658.7670	1658.7648	1.32	0	78	1.8e-08	1	U	R.LAASQAHNEAEFESR.I
511	52 – 59	493.2817	984.5489	984.5464	2.51	1	44	3.7e-05	1	U	S.RINNAIER.L
163	53 – 59	415.2285	828.4424	828.4453	-3.52	0	54	4e-06	1	U	R.INNAIER.L
164	53 – 59	415.2303	828.4461	828.4453	0.88	0	62	5.9e-07	1	U	R.INNAIER.L
380	64 – 71	473.2958	944.5770	944.5807	-3.91	0	77	2e-08	1	U	K.LGVQLLFR.E
381	64 – 71	473.2963	944.5780	944.5807	-2.87	0	59	1.4e-06	1	U	K.LGVQLLFR.E
382	64 – 71	473.2972	944.5798	944.5807	-0.97	0	82	6.2e-09	1	U	K.LGVQLLFR.E
383	64 – 71	473.2974	944.5803	944.5807	-0.44	0	83	5.6e-09	1	U	K.LGVQLLFR.E
384	64 – 71	473.2977	944.5809	944.5807	0.18	0	67	2e-07	1	U	K.LGVQLLFR.E
385	64 – 71	473.2979	944.5813	944.5807	0.62	0	65	3e-07	1	U	K.LGVQLLFR.E
386	64 – 71	473.2980	944.5814	944.5807	0.71	0	75	3.5e-08	1	U	K.LGVQLLFR.E
387	64 – 71	473.2983	944.5820	944.5807	1.36	0	49	1.3e-05	1	U	K.LGVQLLFR.E

Query	Start – End	Observed	Mr(expt)	Mr(calc)	ppm	M	Score	Expect	Rank	U	Peptide
388	64 – 71	473.2989	944.5833	944.5807	2.68	0	44	3.8e-05	1	U	K.LGVQLLFR.E
389	64 – 71	473.2992	944.5838	944.5807	3.27	0	36	0.00025	1	U	K.LGVQLLFR.E
665	72 – 80	519.7623	1037.5100	1037.5142	-4.02	0	60	9.1e-07	1	U	R.EQYTLATGR.A
127	81 – 87	404.7001	807.3857	807.3875	-2.17	0	54	4.2e-06	1	U	R.ADAVYNR.L
128	81 – 87	404.7012	807.3879	807.3875	0.45	0	47	1.9e-05	1	U	R.ADAVYNR.L
129	81 – 87	404.7013	807.3880	807.3875	0.58	0	32	0.00061	1	U	R.ADAVYNR.L
1410	88 – 99	686.8738	1371.7331	1371.7398	-4.87	0	55	3.2e-06	1	U	R.LVIEYEPPGSLR.P
1738	88 – 101	792.4241	1582.8336	1582.8355	-1.21	0	83	5e-09	1	U	R.LVIEYEPPGSLRPN.L
1889	88 – 103	913.0123	1824.0101	1824.0145	-2.41	0	64	4.2e-07	1	U	R.LVIEYEPPGSLRPNLK.H
1890	88 – 103	609.0113	1824.0121	1824.0145	-1.30	0	87	2.2e-09	1	U	R.LVIEYEPPGSLRPNLK.H
1431	113 – 123	691.3423	1380.6701	1380.6707	-0.44	0	87	2.5e-09	1	U	R.QVMNYIEELSR.A
1469	113 – 123	699.3378	1396.6611	1396.6656	-3.22	0	64	5e-07	1	U	R.QVMNYIEELSR.A + Oxidation (M)
1470	113 – 123	699.3385	1396.6625	1396.6656	-2.28	0	61	8.5e-07	1	U	R.QVMNYIEELSR.A + Oxidation (M)
1471	113 – 123	699.3391	1396.6637	1396.6656	-1.42	0	45	3.2e-05	1	U	R.QVMNYIEELSR.A + Oxidation (M)
321	117 – 123	455.2373	908.4601	908.4603	-0.22	0	42	5.6e-05	1	U	N.YIEELSR.A
408	171 – 179	477.2540	952.4934	952.4978	-4.61	0	72	6.9e-08	1	U	R.SLFSLSSGR.A
409	171 – 179	477.2561	952.4977	952.4978	-0.11	0	44	4.6e-05	1	U	R.SLFSLSSGR.A
410	171 – 179	477.2563	952.4980	952.4978	0.24	0	58	1.7e-06	1	U	R.SLFSLSSGR.A
412	171 – 179	477.2568	952.4991	952.4978	1.38	0	32	0.00065	1	U	R.SLFSLSSGR.A
413	171 – 179	477.2569	952.4992	952.4978	1.46	0	52	7e-06	1	U	R.SLFSLSSGR.A
414	171 – 179	477.2575	952.5004	952.4978	2.70	0	42	6.1e-05	1	U	R.SLFSLSSGR.A
48	173 – 179	377.1987	752.3828	752.3817	1.44	0	48	1.5e-05	1	U	L.FSLSSGR.A
2102	180 – 198	1059.0240	2116.0334	2116.0436	-4.85	0	49	1.1e-05	1	U	R.ALIPENLVEDFGSQNDLSR.Q
2104	180 – 198	1059.0268	2116.0390	2116.0436	-2.20	0	111	7.1e-12	1	U	R.ALIPENLVEDFGSQNDLSR.Q
2105	180 – 198	1059.0272	2116.0398	2116.0436	-1.83	0	111	7.9e-12	1	U	R.ALIPENLVEDFGSQNDLSR.Q
2106	180 – 198	706.3540	2116.0403	2116.0436	-1.57	0	71	8.2e-08	1	U	R.ALIPENLVEDFGSQNDLSR.Q
2107	180 – 198	706.3543	2116.0409	2116.0436	-1.27	0	123	4.5e-13	1	U	R.ALIPENLVEDFGSQNDLSR.Q
2109	180 – 198	706.3560	2116.0462	2116.0436	1.21	0	73	5.6e-08	1	U	R.ALIPENLVEDFGSQNDLSR.Q
1656	186 – 198	740.3559	1478.6973	1478.7001	-1.93	0	43	5.5e-05	1	U	N.LVEDFGSQNDLSR.Q
571	203 – 211	337.1774	1008.5104	1008.5141	-3.64	0	55	4e-06	1	U	R.ALYHALQGH.T
572	203 – 211	505.2648	1008.5150	1008.5141	0.89	0	64	3.9e-07	1	U	R.ALYHALQGH.T
1844	203 – 218	439.2300	1752.8909	1752.8907	0.12	0	54	3.6e-06	1	U	R.ALYHALQGHTSDLTAR.L
1845	203 – 218	877.4528	1752.8911	1752.8907	0.21	0	73	5.1e-08	1	U	R.ALYHALQGHTSDLTAR.L
1846	203 – 218	585.3050	1752.8933	1752.8907	1.45	0	95	3.1e-10	1	U	R.ALYHALQGHTSDLTAR.L
1145	207 – 218	635.3308	1268.6470	1268.6473	-0.28	0	70	1.2e-07	1	U	H.ALQGHTSDLTAR.L
1146	207 – 218	423.8901	1268.6485	1268.6473	0.96	0	65	3.6e-07	1	U	H.ALQGHTSDLTAR.L
777	209 – 218	362.5160	1084.5261	1084.5261	-0.046	0	51	8e-06	1	U	L.QGHTSDLTAR.L
1766	225 – 241	827.4103	1652.8060	1652.8046	0.85	0	87	2.1e-09	1	U	Q.IFFGETAGADAAGGELK.H
1455	227 – 241	697.3323	1392.6501	1392.6521	-1.45	0	40	9.1e-05	1	U	F.FGETAGADAAGGELK.H
377	242 – 249	472.7623	943.5100	943.5127	-2.83	1	40	0.00018	1	U	K.HKSELLAF.A
313	244 – 251	453.7560	905.4975	905.4970	0.52	0	43	4.7e-05	1	U	K.SELLAFAR.G
315	244 – 251	453.7566	905.4986	905.4970	1.74	0	38	0.00017	1	U	K.SELLAFAR.G
1180	299 – 310	644.3480	1286.6815	1286.6830	-1.13	0	89	1.2e-09	1	U	L.TTIANLEGEALR.R
779	301 – 310	543.3004	1084.5863	1084.5876	-1.18	0	79	1.2e-08	1	U	T.IANLEGEALR.R
1377	312 – 323	681.8516	1361.6887	1361.6939	-3.86	0	43	7e-05	1	U	R.ELQNLESGGLFR.T
1378	312 – 323	681.8519	1361.6892	1361.6939	-3.49	0	51	1.2e-05	1	U	R.ELQNLESGGLFR.T
1379	312 – 323	681.8528	1361.6910	1361.6939	-2.17	0	38	0.00016	1	U	R.ELQNLESGGLFR.T
1381	312 – 323	681.8542	1361.6938	1361.6939	-0.12	0	66	2.6e-07	1	U	R.ELQNLESGGLFR.T
1382	312 – 323	681.8546	1361.6946	1361.6939	0.53	0	52	9.1e-06	1	U	R.ELQNLESGGLFR.T
1383	312 – 323	681.8548	1361.6951	1361.6939	0.88	0	69	1.9e-07	1	U	R.ELQNLESGGLFR.T
1384	312 – 323	681.8550	1361.6954	1361.6939	1.10	0	48	2.3e-05	1	U	R.ELQNLESGGLFR.T
1387	312 – 323	681.8561	1361.6977	1361.6939	2.76	0	33	0.00063	1	U	R.ELQNLESGGLFR.T
1731	358 – 371	785.3616	1568.7086	1568.7107	-1.32	0	31	0.00086	1	U	R.LAEYNPATVQDDPH.S
1923	358 – 374	628.6323	1882.8751	1882.8809	-3.09	0	99	1.1e-10	1	U	R.LAEYNPATVQDDPHSAR.D
1924	358 – 374	942.4473	1882.8800	1882.8809	-0.49	0	93	4.6e-10	1	U	R.LAEYNPATVQDDPHSAR.D
1925	358 – 374	628.6342	1882.8807	1882.8809	-0.11	0	90	9e-10	1	U	R.LAEYNPATVQDDPHSAR.D
1926	358 – 374	628.6343	1882.8810	1882.8809	0.015	0	39	0.00012	1	U	R.LAEYNPATVQDDPHSAR.D
743	380 – 387	358.8739	1073.6000	1073.6022	-2.00	0	61	9.4e-07	1	U	K.LYHYLLPR.D
434	381 – 387	481.2669	960.5192	960.5181	1.17	0	30	0.00091	1	U	L.YHYLLPR.D
1899	391 – 405	924.9312	1847.8479	1847.8479	0.034	0	84	3.6e-09	1	U	R.HDLGEFYTPDWLAER.L
1900	391 – 405	616.9569	1847.8490	1847.8479	0.60	0	84	3.9e-09	1	U	R.HDLGEFYTPDWLAER.L
1876	432 – 448	901.9954	1801.9763	1801.9760	0.16	0	140	9.5e-15	1	U	R.LLDPACGSGTFLVLAIR.A
1877	432 – 448	601.6665	1801.9777	1801.9760	0.91	0	56	2.7e-06	1	U	R.LLDPACGSGTFLVLAIR.A
1194	437 – 448	647.3499	1292.6852	1292.6911	-4.57	0	69	1.4e-07	1	U	A.CGSGTFLVLAIR.A
905	438 – 448	567.3378	1132.6610	1132.6604	0.45	0	46	2.4e-05	1	U	C.GSGTFLVLAIR.A
1747	471 – 486	798.4598	1594.9050	1594.9043	0.47	0	113	5.2e-12	1	U	N.SVVGIDLNPLAVTAAR.V
431	493 – 500	480.7795	959.5444	959.5440	0.47	0	55	3.6e-06	1	U	A.IADLLPYR.R
2069	502 – 519	681.3815	2041.1225	2041.1208	0.87	1	52	6.9e-06	1	U	R.REVEIPVYLADSILTPAR.G
928	503 – 512	574.2982	1146.5818	1146.5808	0.81	0	44	4.2e-05	1	U	R.EVEIPVYLAD.S
1928	503 – 519	629.3471	1885.0195	1885.0197	-0.064	0	71	8.1e-08	1	U	R.EVEIPVYLADSILTPAR.G
1929	503 – 519	943.5181	1885.0216	1885.0197	1.03	0	75	3.5e-08	1	U	R.EVEIPVYLADSILTPAR.G
1930	503 – 519	943.5182	1885.0218	1885.0197	1.13	0	113	5e-12	1	U	R.EVEIPVYLADSILTPAR.G
1931	503 – 519	629.3488	1885.0245	1885.0197	2.58	0	91	8.5e-10	1	U	R.EVEIPVYLADSILTPAR.G
54	513 – 519	379.2319	756.4493	756.4494	-0.062	0	35	0.0004	1	U	D.SILTPAR.G
527	520 – 528	496.2519	990.4892	990.4883	0.90	0	62	8.2e-07	1	U	R.GEGLFAQNR.R
528	520 – 528	496.2532	990.4919	990.4883	3.63	0	47	2.9e-05	1	U	R.GEGLFAQNR.R
2078	529 – 547	687.4002	2059.1788	2059.1790	-0.064	1	41	7.1e-05	1	U	R.RILETAVGPLPVPEVINSR.A
1944	530 – 547	952.5435	1903.0725	1903.0778	-2.79	0	146	2.6e-15	1	U	R.ILETAVGPLPVPEVINSR.A
1945	530 – 547	635.3659	1903.0760	1903.0778	-0.97	0	112	6.1e-12	1	U	R.ILETAVGPLPVPEVINSR.A

Query	Start – End	Observed	Mr(expt)	Mr(calc)	ppm	M	Score	Expect	Rank	U	Peptide
1104	553 – 562	625.8329	1249.6512	1249.6554	-3.40	0	70	1.4e-07	1	U	R.LTDLLEEYVR.G
1105	553 – 562	625.8336	1249.6527	1249.6554	-2.17	0	57	2.2e-06	1	U	R.LTDLLEEYVR.G
1106	553 – 562	625.8336	1249.6527	1249.6554	-2.13	0	70	1.3e-07	1	U	R.LTDLLEEYVR.G
1107	553 – 562	625.8338	1249.6531	1249.6554	-1.81	0	75	3.5e-08	1	U	R.LTDLLEEYVR.G
1108	553 – 562	625.8347	1249.6548	1249.6554	-0.47	0	82	8e-09	1	U	R.LTDLLEEYVR.G
1109	553 – 562	625.8350	1249.6554	1249.6554	0.011	0	77	2.1e-08	1	U	R.LTDLLEEYVR.G
1110	553 – 562	625.8351	1249.6556	1249.6554	0.12	0	56	2.4e-06	1	U	R.LTDLLEEYVR.G
1111	553 – 562	625.8352	1249.6558	1249.6554	0.30	0	65	3e-07	1	U	R.LTDLLEEYVR.G
1112	553 – 562	625.8352	1249.6558	1249.6554	0.33	0	70	1.1e-07	1	U	R.LTDLLEEYVR.G
1113	553 – 562	625.8352	1249.6559	1249.6554	0.38	0	70	9.9e-08	1	U	R.LTDLLEEYVR.G
1114	553 – 562	625.8352	1249.6559	1249.6554	0.38	0	77	2.2e-08	1	U	R.LTDLLEEYVR.G
1115	553 – 562	625.8352	1249.6559	1249.6554	0.41	0	52	6.2e-06	1	U	R.LTDLLEEYVR.G
1116	553 – 562	625.8353	1249.6561	1249.6554	0.52	0	40	0.0001	1	U	R.LTDLLEEYVR.G
1118	553 – 562	625.8363	1249.6581	1249.6554	2.14	0	52	5.8e-06	1	U	R.LTDLLEEYVR.G
1119	553 – 562	625.8374	1249.6602	1249.6554	3.85	0	69	1.2e-07	1	U	R.LTDLLEEYVR.G
1030	563 – 573	607.2947	1212.5748	1212.5775	-2.18	0	48	2.3e-05	1	U	R.GDFSTEAFLAR.A
1031	563 – 573	607.2955	1212.5765	1212.5775	-0.87	0	94	4.1e-10	1	U	R.GDFSTEAFLAR.A
1033	563 – 573	607.2968	1212.5791	1212.5775	1.31	0	85	3e-09	1	U	R.GDFSTEAFLAR.A
1034	563 – 573	607.2973	1212.5801	1212.5775	2.12	0	70	9.6e-08	1	U	R.GDFSTEAFLAR.A
1035	563 – 573	607.2974	1212.5803	1212.5775	2.33	0	42	6.1e-05	1	U	R.GDFSTEAFLAR.A
590	604 – 612	508.2673	1014.5201	1014.5247	-4.53	0	72	5.7e-08	1	U	R.QGLDGIWAR.V
591	604 – 612	508.2697	1014.5248	1014.5247	0.11	0	72	7.4e-08	1	U	R.QGLDGIWAR.V
592	604 – 612	508.2700	1014.5255	1014.5247	0.82	0	72	6.6e-08	1	U	R.QGLDGIWAR.V
593	604 – 612	508.2706	1014.5267	1014.5247	2.00	0	67	2.1e-07	1	U	R.QGLDGIWAR.V
439	639 – 646	482.2500	962.4855	962.4821	3.48	0	36	0.00034	1	U	W.ESLPQAYR.E
367	662 – 670	469.2367	936.4588	936.4586	0.16	0	73	5.4e-08	1	U	H.SGMDTILGK.G + Oxidation (M)
1718	673 – 686	514.5925	1540.7558	1540.7555	0.18	1	64	3.7e-07	1	U	K.KDASTLMTYAVADR.F
1725	673 – 686	779.3821	1556.7496	1556.7504	-0.56	1	126	2.2e-13	1	U	K.KDASTLMTYAVADR.F + Oxidation (M)
1518	674 – 686	707.3358	1412.6570	1412.6606	-2.56	0	32	0.00066	1	U	K.DASTLMTYAVADR.F
1519	674 – 686	707.3360	1412.6574	1412.6606	-2.22	0	96	2.4e-10	1	U	K.DASTLMTYAVADR.F
1521	674 – 686	707.3380	1412.6614	1412.6606	0.58	0	41	9.1e-05	1	U	K.DASTLMTYAVADR.F
1560	674 – 686	715.3320	1428.6495	1428.6555	-4.19	0	66	2.3e-07	1	U	K.DASTLMTYAVADR.F + Oxidation (M)
1561	674 – 686	715.3338	1428.6530	1428.6555	-1.77	0	98	1.6e-10	1	U	K.DASTLMTYAVADR.F + Oxidation (M)
1562	674 – 686	715.3358	1428.6570	1428.6555	1.07	0	71	1.1e-07	1	U	K.DASTLMTYAVADR.F + Oxidation (M)
1563	674 – 686	715.3360	1428.6575	1428.6555	1.43	0	79	1.7e-08	1	U	K.DASTLMTYAVADR.F + Oxidation (M)
1564	674 – 686	715.3368	1428.6591	1428.6555	2.54	0	79	1.7e-08	1	U	K.DASTLMTYAVADR.F + Oxidation (M)
1089	676 – 686	622.3023	1242.5900	1242.5914	-1.13	0	32	0.00065	1	U	A.STLMTYAVADR.F + Oxidation (M)
1191	694 – 704	646.3738	1290.7330	1290.7336	-0.47	0	74	4.1e-08	1	U	K.LGFLITQSVWK.T
477	697 – 704	487.7874	973.5602	973.5597	0.60	0	65	5.8e-07	1	U	F.LITQSVWK.T
99	705 – 712	397.2008	792.3870	792.3879	-1.09	0	69	1.4e-07	1	U	K.TGAGQGFR.R
532	716 – 724	331.5135	991.5187	991.5199	-1.18	0	46	2.7e-05	1	U	R.IGENGPHLR.V
533	716 – 724	331.5139	991.5198	991.5199	-0.15	0	41	8.3e-05	1	U	R.IGENGPHLR.V
534	716 – 724	496.7675	991.5205	991.5199	0.60	0	78	1.5e-08	1	U	R.IGENGPHLR.V
536	716 – 724	331.5142	991.5207	991.5199	0.76	0	42	6.6e-05	1	U	R.IGENGPHLR.V
264	717 – 724	440.2248	878.4351	878.4359	-0.86	0	57	2.7e-06	1	U	I.GENGPHLR.V
2082	725 – 743	1037.0331	2072.0517	2072.0538	-1.03	0	123	5.3e-13	1	U	R.VLHVDDLSSLQVFEGASTR.T
2083	725 – 743	691.6913	2072.0520	2072.0538	-0.87	0	69	1.3e-07	1	U	R.VLHVDDLSSLQVFEGASTR.T
2084	725 – 743	691.6916	2072.0529	2072.0538	-0.48	0	96	2.8e-10	1	U	R.VLHVDDLSSLQVFEGASTR.T
282	744 – 751	447.2561	892.4976	892.5018	-4.77	0	63	5.6e-07	1	U	R.TSAFVLQK.G
399	744 – 752	475.7682	949.5218	949.5233	-1.53	1	65	3.2e-07	1	U	R.TSAFVLQKG.R
1040	757 – 765	608.8045	1215.5944	1215.5964	-1.65	0	68	1.9e-07	1	U	R.YPVPYTYWK.K
2177	767 – 787	773.3608	2317.0607	2317.0631	-1.05	1	100	1.2e-10	1	U	K.TTKGEGLDYDSTLGEVMEQTK.R + Oxidation (M)
2014	770 – 787	986.4457	1970.8769	1970.8779	-0.52	0	112	6.3e-12	1	U	K.GEGLDYDSTLGEVMEQTK.R
2029	770 – 787	994.4423	1986.8701	1986.8728	-1.36	0	43	5e-05	1	U	K.GEGLDYDSTLGEVMEQTK.R + Oxidation (M)
2030	770 – 787	994.4430	1986.8714	1986.8728	-0.71	0	56	2.6e-06	1	U	K.GEGLDYDSTLGEVMEQTK.R + Oxidation (M)
2031	770 – 787	994.4430	1986.8715	1986.8728	-0.68	0	134	4.2e-14	1	U	K.GEGLDYDSTLGEVMEQTK.R + Oxidation (M)
2032	770 – 787	663.2990	1986.8752	1986.8728	1.21	0	91	8.6e-10	1	U	K.GEGLDYDSTLGEVMEQTK.R + Oxidation (M)
2033	770 – 787	663.2991	1986.8756	1986.8728	1.40	0	85	3.1e-09	1	U	K.GEGLDYDSTLGEVMEQTK.R + Oxidation (M)
2116	770 – 788	709.9986	2126.9739	2126.9790	-2.40	1	88	1.4e-09	1	U	K.GEGLDYDSTLGEVMEQTKR.L
2131	770 – 788	1072.4934	2142.9722	2142.9739	-0.82	1	86	2.3e-09	1	U	K.GEGLDYDSTLGEVMEQTKR.L + Oxidation (M)
2132	770 – 788	715.3316	2142.9729	2142.9739	-0.48	1	116	2.6e-12	1	U	K.GEGLDYDSTLGEVMEQTKR.L + Oxidation (M)
2134	770 – 788	715.3331	2142.9773	2142.9739	1.59	1	47	2.2e-05	1	U	K.GEGLDYDSTLGEVMEQTKR.L + Oxidation (M)
84	791 – 797	392.7036	783.3926	783.3916	1.37	0	31	0.0008	1	U	R.FHAVPVD.P
1269	791 – 802	663.3226	1324.6307	1324.6300	0.57	0	37	0.00024	1	U	R.FHAVPVDPDDLT.S
2124	791 – 809	713.0286	2136.0641	2136.0640	0.013	0	98	2.2e-10	1	U	R.FHAVPVDPDDLTSPWLTAR.R
1905	793 – 809	926.9720	1851.9294	1851.9367	-3.93	0	54	5.4e-06	1	U	H.AVPVDPDDLTSPWLTAR.R

Query	Start – End	Observed	Mr(expt)	Mr(calc)	ppm	M	Score	Expect	Rank	U	Peptide
1403	798 – 809	686.3486	1370.6827	1370.6830	-0.23	0	81	7.8e-09	1	U	D.PDDLTSPWLTAR.R
210	811 – 817	424.7588	847.5030	847.5028	0.19	1	53	5e-06	1	U	R.RALYAVR.K
701	818 – 826	351.5292	1051.5657	1051.5662	-0.49	1	35	0.00034	1	U	R.KVLGTSEYR.A
702	818 – 826	526.7903	1051.5661	1051.5662	-0.056	1	80	1e-08	1	U	R.KVLGTSEYR.A
703	818 – 826	526.7906	1051.5667	1051.5662	0.51	1	39	0.00013	1	U	R.KVLGTSEYR.A
344	819 – 826	462.7424	923.4702	923.4712	-1.15	0	70	1.1e-07	1	U	K.VLGTSEYR.A
12	857 – 863	359.6908	717.3670	717.3657	1.75	0	37	0.00052	1	U	R.NVTEGAK.R
559	890 – 897	501.2457	1000.4769	1000.4767	0.26	0	48	1.8e-05	1	U	R.WYAQPSLH.I
1971	890 – 905	642.6679	1924.9817	1924.9869	-2.70	0	43	4.9e-05	1	U	R.WYAQPSLHILMVQDPK.T
1992	890 – 905	648.0014	1940.9825	1940.9818	0.33	0	69	1.3e-07	1	U	R.WYAQPSLHILMVQDPK.T + Oxidation (M)
426	898 – 905	480.2655	958.5164	958.5157	0.71	0	60	1.2e-06	1	U	H.ILMVQDPK.T + Oxidation (M)
991	908 – 917	395.8912	1184.6519	1184.6513	0.47	1	54	4.3e-06	1	U	R.RGIDEQVLQK.R
992	908 – 917	593.3335	1184.6524	1184.6513	0.90	1	57	1.9e-06	1	U	R.RGIDEQVLQK.R
637	909 – 917	515.2826	1028.5507	1028.5502	0.47	0	68	1.9e-07	1	U	R.GIDEQVLQK.R
638	909 – 917	515.2828	1028.5511	1028.5502	0.82	0	67	2e-07	1	U	R.GIDEQVLQK.R
639	909 – 917	515.2830	1028.5514	1028.5502	1.11	0	65	3.4e-07	1	U	R.GIDEQVLQK.R
993	909 – 918	395.8915	1184.6528	1184.6513	1.25	1	46	2.9e-05	1	U	R.GIDEQVLQKR.Y
83	922 – 927	391.2139	780.4132	780.4170	-4.85	0	46	4.1e-05	1	U	K.TWAYLK.R
281	928 – 934	445.7620	889.5095	889.5134	-4.31	1	37	0.00021	1	U	K.RFEAVLR.E
31	929 – 934	367.7123	733.4101	733.4123	-3.00	0	51	8.1e-06	1	U	R.FEAVLR.E
34	929 – 934	367.7137	733.4128	733.4123	0.76	0	48	1.8e-05	1	U	R.FEAVLR.E
1291	952 – 962	670.7809	1339.5472	1339.5465	0.56	0	33	0.0005	1	U	R.MVETGPFYSMF.N + 2 Oxidation (M)
1202	963 – 973	649.3137	1296.6129	1296.6139	-0.76	0	66	2.3e-07	1	U	F.NVGDYTFAPWK.V
1914	978 – 995	933.9737	1865.9329	1865.9411	-4.38	0	37	0.0002	1	U	R.YVASDFIVAVVGPASDEK.P
2196	978 – 1002	877.4620	2629.3642	2629.3639	0.084	0	125	3.5e-13	1	U	R.YVASDFIVAVVGPASDEKPVVPNEK.L
2198	978 – 1002	877.4642	2629.3709	2629.3639	2.64	0	36	0.00023	1	U	R.YVASDFIVAVVGPASDEKPVVPNEK.L
1210	1028 – 1038	651.3300	1300.6454	1300.6452	0.11	0	39	0.00013	1	U	R.FAVQSFFVQTQ.I
1816	1028 – 1042	860.4461	1718.8777	1718.8781	-0.22	0	97	2.9e-10	1	U	R.FAVQSFFVQTQIAPH.V
1817	1028 – 1042	573.9669	1718.8789	1718.8781	0.46	0	90	1.5e-09	1	U	R.FAVQSFFVQTQIAPH.V
2158	1028 – 1046	730.0689	2187.1849	2187.1841	0.37	0	81	8e-09	1	U	R.FAVQSFFVQTQIAPHVLQK.L
305	1039 – 1046	302.5241	904.5506	904.5494	1.28	0	31	0.00078	1	U	Q.IAPHVLQK.L
1154	1052 – 1061	425.1895	1272.5467	1272.5483	-1.26	0	54	3.6e-06	1	U	R.YEPNTDHQNR.I
1155	1052 – 1061	425.1903	1272.5490	1272.5483	0.53	0	36	0.00024	1	U	R.YEPNTDHQNR.I
1690	1068 – 1081	378.9442	1511.7477	1511.7480	-0.21	1	50	1.1e-05	1	U	R.RAHELAPAAYNGDK.A
1351	1069 – 1081	452.8895	1355.6468	1355.6469	-0.12	0	91	7.9e-10	1	U	R.AHELAPAAYNGDK.A
1352	1069 – 1081	678.8309	1355.6472	1355.6469	0.20	0	106	2.3e-11	1	U	R.AHELAPAAYNGDK.A
675	1089 – 1096	349.1807	1044.5203	1044.5200	0.32	1	34	0.00049	1	U	R.RVEEEIDR.A
676	1089 – 1096	349.1810	1044.5210	1044.5200	1.04	1	40	0.00011	1	U	R.RVEEEIDR.A
1941	1097 – 1113	950.4944	1898.9743	1898.9737	0.30	0	45	3.1e-05	1	U	R.AAAQLWGLTEEELAEIR.R
1942	1097 – 1113	634.0001	1898.9786	1898.9737	2.54	0	44	4.1e-05	1	U	R.AAAQLWGLTEEELAEIR.R
2076	1097 – 1114	686.0331	2055.0776	2055.0748	1.33	1	55	2.9e-06	1	U	R.AAAQLWGLTEEELAEIRR.S
113	1115 – 1121	402.2149	802.4153	802.4185	-4.00	1	49	3.5e-05	1	U	R.SLEELRG.-
115	1115 – 1121	402.2163	802.4180	802.4185	-0.61	1	45	7.4e-05	1	U	R.SLEELRG.-
117	1115 – 1121	402.2167	802.4188	802.4185	0.41	1	49	3.3e-05	1	U	R.SLEELRG.-

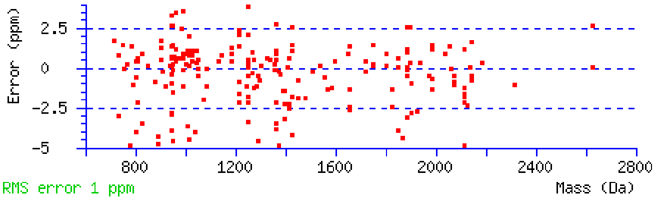

Mascot: http://www.matrixscience.com/
