# Supplementary material for: Thermostable proteins bioprocesses: The activity of restriction endonuclease-methyltransferase from Thermus thermophilus (RM.TthHB27I) cloned in Escherichia coli is critically affected by the codon composition of the synthetic gene
Source: PLoS One. 2017 Oct 17;12(10):e0186633. doi: 10.1371/journal.pone.0186633 (PMC5645126; doi:10.1371/journal.pone.0186633)
Supplement: S4 File — (PDF) [file pone.0186633.s004.pdf]

MASCOT Search Results

Protein View: 366

RM.TthHB27I [agafab 20160615]

**Database:**	small-www
**Score:**	9301
**Nominal mass (M_r):**	127974
**Calculated pI:**	6.77

Sequence similarity is available as an NCBI BLAST search of 366 against nr.

Search parameters

**MS data file:**	\\212.87.29.243\Dane\USERS\Agata\tmp\60614970kref_rec_TthHB27I.mgf
**Enzyme:**	semiTrypsin: cuts C-term side of KR unless next residue is P.<br>Cleavage is semi-specific. (Peptide can be non-specific at one terminus only.)
**Fixed modifications:**	Carbamidomethyl (C)
**Variable modifications:**	Oxidation (M)

Protein sequence coverage: 66%

Matched peptides shown in ***bold red***.

```
   1 MLSLLTGGVF RRVKLMNWID LYTHLKQEVP WFFNSVRLAA SQAHNEAEFE
  51 SRINNAIERL AQKLGVQLLF REQYTLATGR ADAVYNRLVI EYEPPGSLRP
 101 NLKHSHTQHA VRQVMNYIEE LSRAERHDRD RLLGVVFDGH YFIFVRYHEG
 151 HWIVEEPLEV NPASCERFLR SLFSLSSGRA LIPENLVEDF GSQNDLSRQA
 201 TRALYHALQG HTSDLTARLF VQWQIFFGET AGADAAGGEL KHKSELLAFA
 251 RGMGLRGSRI DMPRFLFALH TYFSFLVKNI ARLVLQAYAG GGLGTTPLTT
 301 IANLEGEALR RELQNLESGG LFRTLGLKNL LEGDFFAWYL DAWNPEVEEA
 351 LRQVLARLAE YNPATVQDDP HSARDLLKKL YHYLLPRDIR HDLGEFYTPD
 401 WLAERLLNQL GEPWFIMPPG NHPPRGLPDK RLLDPACGSG TFLVLAIRAL
 451 KVNCFLAGFS EADTLEVILN SVVGIDLNPL AVTAARVNYL LAIADLLPYR
 501 RREVEIPVYL ADSILTPARG EGLFAQNRRI LETAVGPLPV PEVINSRAKM
 551 ERLTDLLEEY VRGDFSTEAF LARAKKEIPD LADALHADEV LTELYERLRD
 601 LHRQGLDGIW ARVLKNAFMP LFLEPFDYVV GNPPWINWES LPQAYREQTA
 651 ELWTCYGLFV HSGMDTILGK GKKDASTLMT YAVADRFLKE GGKLGFLITQ
 701 SVWKTGAGQG FRRFRIGENG PHLRVLHVDD LSSLQVFEGA STRTSAFVLQ
 751 KGRPTRYPVP YTYWKKTTKG EGLDYDSTLG EVMEQTKRLR FHAVPVDPDD
 801 LTSPWLTARR RALYAVRKVL GTSEYRAYEG ANSGGANGIY WLEILAERPD
 851 GLVVVRNVTE GAKREVEGIT TELEPDLLYP LLRGRDVRRW YAQPSLHILM
 901 VQDPKTRRGI DEQVLQKRYP KTWAYLKRFE AVLRERSGFR RYFTRKDRNG
 951 RMVETGPFYS MFNVGDYTFA PWKVVWRYVA SDFIVAVVGP ASDEKPVVPN
1001 EKLMLVPVED DNEAFYLCGV LNSSPIRFAV QSFFVQTQIA PHVLQKLCIP
1051 RYEPNTDHQN RIAHLSRRAH ELAPAAYNGD KAARAELRRV EEEIDRAAAQ
1101 LWGLTEEELA EIRRSLEELR G
```

Unformatted sequence string: 1121 residues (for pasting into other applications).

Sort peptides by ◉ Residue Number ○ Increasing Mass ○ Decreasing Mass

Query	Start – End	Observed	Mr(expt)	Mr(calc)	ppm	M	Score	Expect	Rank	U	Peptide
1370	1 – 11	597.3395	1192.6644	1192.6638	0.48	0	98	1.6e-10	1	U	-.MLSLLTGGVFR.R
1409	1 – 11	605.3337	1208.6528	1208.6587	-4.94	0	64	5.5e-07	1	U	-.MLSLLTGGVFR.R + Oxidation (M)
1411	1 – 11	605.3355	1208.6564	1208.6587	-1.95	0	53	6.1e-06	1	U	-.MLSLLTGGVFR.R + Oxidation (M)
1412	1 – 11	605.3360	1208.6574	1208.6587	-1.10	0	60	1.2e-06	1	U	-.MLSLLTGGVFR.R + Oxidation (M)
983	2 – 11	531.8188	1061.6230	1061.6233	-0.32	0	85	2.9e-09	1	U	M.LSLLTGGVFR.R
390	4 – 11	431.7604	861.5062	861.5072	-1.23	0	70	9.2e-08	1	U	S.LLTGGVFR.R
74	5 – 11	375.2189	748.4232	748.4232	-0.0013	0	50	2.1e-05	1	U	L.LTGGVFR.R
2128	16 – 26	483.9112	1448.7116	1448.7122	-0.40	0	37	0.00044	1	U	L.MNWIDLYTHLK.Q + Oxidation (M)
992	27 – 34	533.7535	1065.4924	1065.4920	0.43	0	33	0.00051	1	U	K.QEVPWFFN.S
837	38 – 47	506.2472	1010.4799	1010.4781	1.82	0	68	1.5e-07	1	U	R.LAASQAHNEA.E
2503	38 – 52	553.9263	1658.7572	1658.7648	-4.62	0	91	8e-10	1	U	R.LAASQAHNEAEFESR.I
2505	38 – 52	830.3900	1658.7655	1658.7648	0.41	0	147	1.9e-15	1	U	R.LAASQAHNEAEFESR.I
2507	38 – 52	553.9298	1658.7677	1658.7648	1.71	0	51	8.6e-06	1	U	R.LAASQAHNEAEFESR.I
735	45 – 52	491.2179	980.4213	980.4199	1.45	0	56	2.7e-06	1	U	H.NEAEFESR.I
290	53 – 59	415.2283	828.4420	828.4453	-4.06	0	53	4.7e-06	1	U	R.INNAIER.L
291	53 – 59	415.2298	828.4450	828.4453	-0.41	0	44	3.8e-05	1	U	R.INNAIER.L
292	53 – 59	415.2301	828.4456	828.4453	0.34	0	54	4.1e-06	1	U	R.INNAIER.L
293	53 – 59	415.2303	828.4460	828.4453	0.80	0	39	0.00012	1	U	R.INNAIER.L

Query	Start - End	Observed	Mr(expt)	Mr(calc)	ppm	M	Score	Expect	Rank	U	Peptide
294	53 - 59	415.2305	828.4465	828.4453	1.45	0	41	7.2e-05	1	U	R.INNAIER.L
608	64 - 71	473.2971	944.5796	944.5807	-1.25	0	53	5e-06	1	U	K.LGVQLLFR.E
609	64 - 71	473.2971	944.5796	944.5807	-1.23	0	82	6.8e-09	1	U	K.LGVQLLFR.E
610	64 - 71	473.2979	944.5812	944.5807	0.51	0	82	6.9e-09	1	U	K.LGVQLLFR.E
916	72 - 80	519.7620	1037.5094	1037.5142	-4.60	0	49	1.3e-05	1	U	R.EQYTLATGR.A
1896	88 - 99	686.8771	1371.7397	1371.7398	-0.083	0	83	5.3e-09	1	U	R.LVIEYEPPGSLR.P
2404	88 - 101	792.4250	1582.8354	1582.8355	-0.068	0	77	2.1e-08	1	U	R.LVIEYEPPGSLRPN.L
2405	88 - 101	528.6192	1582.8357	1582.8355	0.16	0	54	4.2e-06	1	U	R.LVIEYEPPGSLRPN.L
2689	88 - 103	609.0116	1824.0129	1824.0145	-0.88	0	37	0.0002	1	U	R.LVIEYEPPGSLRPNLK.H
2690	88 - 103	913.0139	1824.0132	1824.0145	-0.68	0	86	2.6e-09	1	U	R.LVIEYEPPGSLRPNLK.H
2691	88 - 103	609.0118	1824.0135	1824.0145	-0.52	0	84	3.6e-09	1	U	R.LVIEYEPPGSLRPNLK.H
1923	113 - 123	691.3417	1380.6688	1380.6707	-1.38	0	87	2.6e-09	1	U	R.QVMNYIEELSR.A
1970	113 - 123	699.3386	1396.6626	1396.6656	-2.18	0	62	6.4e-07	1	U	R.QVMNYIEELSR.A + Oxidation (M)
1972	113 - 123	699.3392	1396.6639	1396.6656	-1.22	0	56	2.5e-06	1	U	R.QVMNYIEELSR.A + Oxidation (M)
1974	113 - 123	699.3401	1396.6656	1396.6656	-0.032	0	50	9.9e-06	1	U	R.QVMNYIEELSR.A + Oxidation (M)
882	116 - 123	512.2595	1022.5045	1022.5032	1.19	0	54	4.1e-06	1	U	M.NYIEELSR.A
510	117 - 123	455.2369	908.4592	908.4603	-1.27	0	55	3.5e-06	1	U	N.YIEELSR.A
3221	147 - 167	851.0636	2550.1689	2550.1597	3.60	0	30	0.00096	1	U	R.YHEGHWIVEEPLEVNPASCER.F
639	171 - 179	477.2546	952.4947	952.4978	-3.25	0	40	9.2e-05	1	U	R.SLFSLSSGR.A
640	171 - 179	477.2561	952.4977	952.4978	-0.075	0	53	5.4e-06	1	U	R.SLFSLSSGR.A
641	171 - 179	477.2564	952.4982	952.4978	0.41	0	52	6.1e-06	1	U	R.SLFSLSSGR.A
642	171 - 179	477.2564	952.4983	952.4978	0.58	0	48	1.7e-05	1	U	R.SLFSLSSGR.A
644	171 - 179	477.2573	952.5000	952.4978	2.28	0	48	1.8e-05	1	U	R.SLFSLSSGR.A
85	173 - 179	377.1978	752.3811	752.3817	-0.81	0	54	4.2e-06	1	U	L.FSLSSGR.A
3009	180 - 198	706.3523	2116.0351	2116.0436	-4.05	0	120	1.1e-12	1	U	R.ALIPENLVEDFGSQNDLSR.Q
3010	180 - 198	706.3527	2116.0362	2116.0436	-3.50	0	86	2.3e-09	1	U	R.ALIPENLVEDFGSQNDLSR.Q
3012	180 - 198	1059.0268	2116.0390	2116.0436	-2.18	0	141	8.1e-15	1	U	R.ALIPENLVEDFGSQNDLSR.Q
3014	180 - 198	1059.0294	2116.0442	2116.0436	0.28	0	145	3.1e-15	1	U	R.ALIPENLVEDFGSQNDLSR.Q
3015	180 - 198	706.3554	2116.0442	2116.0436	0.28	0	43	5.2e-05	1	U	R.ALIPENLVEDFGSQNDLSR.Q
3016	180 - 198	1059.0300	2116.0454	2116.0436	0.85	0	105	3.1e-11	1	U	R.ALIPENLVEDFGSQNDLSR.Q
2417	185 - 198	797.3802	1592.7459	1592.7431	1.80	0	46	2.7e-05	1	U	E.NLVEDFGSQNDLSR.Q
830	203 - 211	337.1786	1008.5139	1008.5141	-0.24	0	65	3.5e-07	1	U	R.ALYHALQGH.T
831	203 - 211	505.2653	1008.5161	1008.5141	2.01	0	52	5.8e-06	1	U	R.ALYHALQGH.T
2604	203 - 218	439.2302	1752.8918	1752.8907	0.62	0	61	7.5e-07	1	U	R.ALYHALQGHTSDLTAR.L
2606	203 - 218	585.3050	1752.8931	1752.8907	1.35	0	93	5.5e-10	1	U	R.ALYHALQGHTSDLTAR.L
1592	207 - 218	423.8895	1268.6467	1268.6473	-0.48	0	67	2.4e-07	1	U	H.ALQGHTSDLTAR.L
1593	207 - 218	635.3309	1268.6473	1268.6473	0.030	0	82	7.5e-09	1	U	H.ALQGHTSDLTAR.L
1044	209 - 218	362.5157	1084.5253	1084.5261	-0.76	0	55	3.5e-06	1	U	L.QGHTSDLTAR.L
114	212 - 218	382.2001	762.3856	762.3872	-2.11	0	51	8.6e-06	1	U	H.TSDLTAR.L
2494	225 - 241	827.4106	1652.8066	1652.8046	1.18	0	124	4.2e-13	1	U	Q.IFFGETAGADAAGGELK.H
1951	227 - 241	697.3344	1392.6543	1392.6521	1.60	0	115	3.5e-12	1	U	F.FGETAGADAAGGELK.H
505	244 - 251	453.7555	905.4964	905.4970	-0.68	0	71	7.7e-08	1	U	K.SELLAFAR.G
506	244 - 251	453.7561	905.4976	905.4970	0.65	0	61	7.1e-07	1	U	K.SELLAFAR.G
507	244 - 251	453.7563	905.4980	905.4970	1.11	0	46	2.4e-05	1	U	K.SELLAFAR.G
3002	289 - 310	1056.5684	2111.1223	2111.1222	0.017	0	133	4.9e-14	1	U	Y.AGGGLGTTPLTTIANLEGEALR.R
3003	289 - 310	704.7149	2111.1228	2111.1222	0.25	0	80	9.8e-09	1	U	Y.AGGGLGTTPLTTIANLEGEALR.R
2610	294 - 310	878.9774	1755.9403	1755.9367	2.05	0	75	3.1e-08	1	U	L.GTTPLTTIANLEGEALR.R
1635	299 - 310	644.3479	1286.6812	1286.6830	-1.37	0	89	1.2e-09	1	U	L.TTIANLEGEALR.R
1636	299 - 310	644.3501	1286.6856	1286.6830	2.02	0	44	4e-05	1	U	L.TTIANLEGEALR.R
2106	299 - 311	481.9355	1442.7846	1442.7841	0.37	1	37	0.00021	1	U	L.TTIANLEGEALRR.E
1358	300 - 310	593.8253	1185.6361	1185.6353	0.66	0	72	6.2e-08	1	U	T.TIANLEGEALR.R
1045	301 - 310	543.3016	1084.5886	1084.5876	0.88	0	81	7.5e-09	1	U	T.IANLEGEALR.R
158	304 - 310	394.2191	786.4236	786.4235	0.074	0	55	9.4e-06	1	U	N.LEGEALR.R
1862	312 - 323	681.8520	1361.6894	1361.6939	-3.33	0	48	2.2e-05	1	U	R.ELQNLESGGLFR.T
1863	312 - 323	681.8531	1361.6917	1361.6939	-1.60	0	40	0.00011	1	U	R.ELQNLESGGLFR.T
1864	312 - 323	681.8546	1361.6947	1361.6939	0.60	0	52	8.5e-06	1	U	R.ELQNLESGGLFR.T
1865	312 - 323	681.8553	1361.6960	1361.6939	1.50	0	64	5.9e-07	1	U	R.ELQNLESGGLFR.T
2557	339 - 352	852.9127	1703.8108	1703.8155	-2.74	0	61	7.6e-07	1	U	W.YLDAWNPEVEEALR.Q
1770	358 - 369	668.3073	1334.6001	1334.5990	0.78	0	49	1.2e-05	1	U	R.LAEYNPATVQDD.P
2389	358 - 371	785.3633	1568.7121	1568.7107	0.90	0	71	8.1e-08	1	U	R.LAEYNPATVQDDPH.S
2732	358 - 374	628.6315	1882.8726	1882.8809	-4.45	0	99	1.1e-10	1	U	R.LAEYNPATVQDDPHSAR.D
2733	358 - 374	628.6338	1882.8794	1882.8809	-0.80	0	52	5.9e-06	1	U	R.LAEYNPATVQDDPHSAR.D
2734	358 - 374	942.4490	1882.8834	1882.8809	1.30	0	107	2.1e-11	1	U	R.LAEYNPATVQDDPHSAR.D
2735	358 - 374	628.6353	1882.8839	1882.8809	1.59	0	93	5.5e-10	1	U	R.LAEYNPATVQDDPHSAR.D
1002	380 - 387	358.8746	1073.6019	1073.6022	-0.25	0	66	3.2e-07	1	U	K.LYHYLLPR.D
2708	391 - 405	924.9307	1847.8469	1847.8479	-0.51	0	60	1.1e-06	1	U	R.HDLGEFYTPDWLAER.L
2709	391 - 405	616.9570	1847.8492	1847.8479	0.74	0	75	3e-08	1	U	R.HDLGEFYTPDWLAER.L
3161	406 - 425	771.7375	2312.1908	2312.1888	0.87	0	72	6e-08	1	U	R.LLNQLGEPWFIMPPGNHPPR.G
3169	406 - 425	777.0690	2328.1851	2328.1837	0.60	0	98	1.7e-10	1	U	R.LLNQLGEPWFIMPPGNHPPR.G + Oxidation (M)
2668	432 - 448	901.9953	1801.9760	1801.9760	-0.037	0	151	7.7e-16	1	U	R.LLDPACGSGTFLVLAIR.A
2669	432 - 448	601.6664	1801.9773	1801.9760	0.69	0	122	5.8e-13	1	U	R.LLDPACGSGTFLVLAIR.A
1658	437 - 448	647.3527	1292.6909	1292.6911	-0.12	0	106	2.5e-11	1	U	A.CGSGTFLVLAIR.A
1222	438 - 448	567.3367	1132.6589	1132.6604	-1.39	0	34	0.00043	1	U	C.GSGTFLVLAIR.A
2421	471 - 486	798.4597	1594.9048	1594.9043	0.35	0	106	2.7e-11	1	U	N.SVVGIDLNPLAVTAAR.V
1556	475 - 486	627.3646	1252.7147	1252.7139	0.59	0	35	0.00035	1	U	G.IDLNPLAVTAAR.V
189	479 - 486	399.7454	797.4763	797.4759	0.46	0	43	5.2e-05	1	U	N.PLAVTAAR.V
1563	490 - 500	629.3795	1256.7444	1256.7492	-3.86	0	65	3.5e-07	1	U	Y.LLAIADLLPYR.R
1259	491 - 500	572.8412	1143.6679	1143.6652	2.40	0	58	1.7e-06	1	U	L.LAIADLLPYR.R

Query	Start - End	Observed	Mr(expt)	Mr(calc)	ppm	M	Score	Expect	Rank	U	Peptide
913	492 - 500	516.3000	1030.5854	1030.5811	4.13	0	58	1.6e-06	1	U	L.AIADLLPYR.R
665	493 - 500	480.7789	959.5432	959.5440	-0.84	0	57	2.6e-06	1	U	A.IADLLPYR.R
2738	503 - 519	943.5155	1885.0165	1885.0197	-1.68	0	107	2e-11	1	U	R.EVEIPVYLADSILTPAR.G
2739	503 - 519	629.3462	1885.0167	1885.0197	-1.55	0	117	1.8e-12	1	U	R.EVEIPVYLADSILTPAR.G
2740	503 - 519	943.5170	1885.0194	1885.0197	-0.11	0	109	1.4e-11	1	U	R.EVEIPVYLADSILTPAR.G
2742	503 - 519	629.3473	1885.0201	1885.0197	0.21	0	88	1.6e-09	1	U	R.EVEIPVYLADSILTPAR.G
94	513 - 519	379.2315	756.4485	756.4494	-1.14	0	46	3.4e-05	1	U	D.SILTPAR.G
770	520 - 528	496.2492	990.4838	990.4883	-4.57	0	60	1.3e-06	1	U	R.GEGLFAQNR.R
2685	529 - 545	909.0313	1816.0480	1816.0458	1.22	1	33	0.00047	1	U	R.RILETAVGPLPVPEVIN.S
2963	529 - 547	687.4001	2059.1785	2059.1790	-0.21	1	83	5.2e-09	1	U	R.RILETAVGPLPVPEVINSR.A
2513	530 - 545	830.9802	1659.9459	1659.9447	0.72	0	60	1e-06	1	U	R.ILETAVGPLPVPEVIN.S
2764	530 - 547	635.3659	1903.0757	1903.0778	-1.11	0	109	1.3e-11	1	U	R.ILETAVGPLPVPEVINSR.A
2765	530 - 547	952.5474	1903.0802	1903.0778	1.23	0	36	0.00026	1	U	R.ILETAVGPLPVPEVINSR.A
2122	534 - 547	724.4171	1446.8196	1446.8195	0.080	0	55	3e-06	1	U	T.AVGPLPVPEVINSR.A
1906	535 - 547	688.8956	1375.7767	1375.7823	-4.13	0	48	2.3e-05	1	U	A.VGPLPVPEVINSR.A
1520	553 - 562	625.8321	1249.6496	1249.6554	-4.64	0	60	1.1e-06	1	U	R.LTDLLEEYVR.G
1521	553 - 562	625.8330	1249.6514	1249.6554	-3.23	0	58	2.3e-06	1	U	R.LTDLLEEYVR.G
1522	553 - 562	625.8330	1249.6514	1249.6554	-3.23	0	65	3.8e-07	1	U	R.LTDLLEEYVR.G
1523	553 - 562	625.8342	1249.6538	1249.6554	-1.26	0	69	1.6e-07	1	U	R.LTDLLEEYVR.G
1524	553 - 562	625.8343	1249.6541	1249.6554	-1.04	0	70	1.4e-07	1	U	R.LTDLLEEYVR.G
1525	553 - 562	625.8345	1249.6544	1249.6554	-0.83	0	65	4.1e-07	1	U	R.LTDLLEEYVR.G
1526	553 - 562	625.8345	1249.6545	1249.6554	-0.75	0	55	4.4e-06	1	U	R.LTDLLEEYVR.G
1527	553 - 562	625.8346	1249.6547	1249.6554	-0.59	0	75	3.9e-08	1	U	R.LTDLLEEYVR.G
1528	553 - 562	625.8346	1249.6547	1249.6554	-0.56	0	65	4.1e-07	1	U	R.LTDLLEEYVR.G
1529	553 - 562	625.8349	1249.6553	1249.6554	-0.11	0	76	2.9e-08	1	U	R.LTDLLEEYVR.G
1530	553 - 562	625.8350	1249.6554	1249.6554	0.035	0	69	1.4e-07	1	U	R.LTDLLEEYVR.G
1531	553 - 562	625.8350	1249.6555	1249.6554	0.099	0	73	5.6e-08	1	U	R.LTDLLEEYVR.G
1532	553 - 562	625.8351	1249.6557	1249.6554	0.21	0	76	2.5e-08	1	U	R.LTDLLEEYVR.G
1533	553 - 562	625.8353	1249.6560	1249.6554	0.47	0	70	1.1e-07	1	U	R.LTDLLEEYVR.G
1534	553 - 562	625.8353	1249.6560	1249.6554	0.48	0	82	5.9e-09	1	U	R.LTDLLEEYVR.G
1535	553 - 562	625.8355	1249.6564	1249.6554	0.77	0	89	1.3e-09	1	U	R.LTDLLEEYVR.G
1536	553 - 562	625.8355	1249.6564	1249.6554	0.79	0	73	5.3e-08	1	U	R.LTDLLEEYVR.G
1537	553 - 562	625.8356	1249.6566	1249.6554	0.95	0	61	7.8e-07	1	U	R.LTDLLEEYVR.G
1538	553 - 562	625.8356	1249.6566	1249.6554	0.98	0	62	6e-07	1	U	R.LTDLLEEYVR.G
1539	553 - 562	625.8356	1249.6567	1249.6554	1.03	0	77	2e-08	1	U	R.LTDLLEEYVR.G
1540	553 - 562	625.8357	1249.6568	1249.6554	1.12	0	65	3e-07	1	U	R.LTDLLEEYVR.G
1541	553 - 562	625.8357	1249.6568	1249.6554	1.14	0	82	5.8e-09	1	U	R.LTDLLEEYVR.G
1542	553 - 562	625.8360	1249.6574	1249.6554	1.59	0	75	3.2e-08	1	U	R.LTDLLEEYVR.G
1543	553 - 562	625.8370	1249.6595	1249.6554	3.30	0	41	8.7e-05	1	U	R.LTDLLEEYVR.G
1544	553 - 562	625.8371	1249.6596	1249.6554	3.36	0	76	2.8e-08	1	U	R.LTDLLEEYVR.G
1545	553 - 562	625.8374	1249.6602	1249.6554	3.88	0	75	2.9e-08	1	U	R.LTDLLEEYVR.G
1423	563 - 573	607.2963	1212.5780	1212.5775	0.40	0	67	2.1e-07	1	U	R.GDFSTEAFLAR.A
1424	563 - 573	607.2965	1212.5784	1212.5775	0.75	0	88	1.7e-09	1	U	R.GDFSTEAFLAR.A
1425	563 - 573	607.2979	1212.5812	1212.5775	3.09	0	34	0.0004	1	U	R.GDFSTEAFLAR.A
1289	564 - 573	578.7850	1155.5554	1155.5560	-0.55	0	53	4.7e-06	1	U	G.DFSTEAFLAR.A
1620	577 - 588	640.3128	1278.6111	1278.6092	1.50	0	43	4.5e-05	1	U	K.EIPDLADALHAD.E
847	604 - 612	508.2673	1014.5201	1014.5247	-4.54	0	72	6.8e-08	1	U	R.QGLDGIWAR.V
848	604 - 612	508.2685	1014.5225	1014.5247	-2.18	0	74	4e-08	1	U	R.QGLDGIWAR.V
849	604 - 612	508.2693	1014.5241	1014.5247	-0.56	0	70	1.1e-07	1	U	R.QGLDGIWAR.V
850	604 - 612	508.2703	1014.5260	1014.5247	1.27	0	72	5.7e-08	1	U	R.QGLDGIWAR.V
2247	657 - 670	497.5948	1489.7625	1489.7599	1.70	0	55	6e-06	1	U	Y.GLFVHSGMDTILGK.G + Oxidation (M)
582	662 - 670	469.2362	936.4579	936.4586	-0.79	0	88	1.7e-09	1	U	H.SGMDTILGK.G + Oxidation (M)
2351	673 - 686	514.5924	1540.7554	1540.7555	-0.10	1	68	1.6e-07	1	U	K.KDASTLMTYAVADR.F
2027	674 - 686	707.3343	1412.6540	1412.6606	-4.66	0	102	6.3e-11	1	U	K.DASTLMTYAVADR.F
2029	674 - 686	471.8948	1412.6624	1412.6606	1.32	0	57	2.1e-06	1	U	K.DASTLMTYAVADR.F
2069	674 - 686	715.3325	1428.6505	1428.6555	-3.52	0	46	2.3e-05	1	U	K.DASTLMTYAVADR.F + Oxidation (M)
2070	674 - 686	715.3334	1428.6522	1428.6555	-2.27	0	96	2.4e-10	1	U	K.DASTLMTYAVADR.F + Oxidation (M)
2071	674 - 686	715.3367	1428.6589	1428.6555	2.39	0	53	5.8e-06	1	U	K.DASTLMTYAVADR.F + Oxidation (M)
1290	677 - 686	578.7874	1155.5603	1155.5594	0.77	0	66	2.7e-07	1	U	S.TLMTYAVADR.F + Oxidation (M)
1649	694 - 704	646.3717	1290.7289	1290.7336	-3.62	0	74	3.7e-08	1	U	K.LGFLITQSVWK.T
1650	694 - 704	646.3739	1290.7332	1290.7336	-0.33	0	40	9.4e-05	1	U	K.LGFLITQSVWK.T
1653	694 - 704	646.3746	1290.7347	1290.7336	0.88	0	33	0.00051	1	U	K.LGFLITQSVWK.T
1654	694 - 704	646.3748	1290.7350	1290.7336	1.11	0	53	5.6e-06	1	U	K.LGFLITQSVWK.T
714	697 - 704	487.7872	973.5598	973.5597	0.16	0	65	6.3e-07	1	U	F.LITQSVWK.T
171	705 - 712	397.2012	792.3878	792.3879	-0.13	0	37	0.00018	1	U	K.TGAGQGFR.R
172	705 - 712	397.2015	792.3885	792.3879	0.80	0	44	3.7e-05	1	U	K.TGAGQGFR.R
173	705 - 712	397.2016	792.3886	792.3879	0.90	0	35	0.00035	1	U	K.TGAGQGFR.R
174	705 - 712	397.2019	792.3893	792.3879	1.86	0	37	0.00018	1	U	K.TGAGQGFR.R
778	716 - 724	331.5124	991.5152	991.5199	-4.71	0	46	2.7e-05	1	U	R.IGENGPHLR.V
779	716 - 724	331.5127	991.5163	991.5199	-3.65	0	41	8.2e-05	1	U	R.IGENGPHLR.V
780	716 - 724	496.7661	991.5176	991.5199	-2.31	0	50	1e-05	1	U	R.IGENGPHLR.V
781	716 - 724	496.7668	991.5190	991.5199	-0.88	0	34	0.00036	1	U	R.IGENGPHLR.V
782	716 - 724	496.7668	991.5191	991.5199	-0.86	0	81	8.7e-09	1	U	R.IGENGPHLR.V
783	716 - 724	331.5140	991.5202	991.5199	0.28	0	41	7.8e-05	1	U	R.IGENGPHLR.V
442	717 - 724	440.2246	878.4347	878.4359	-1.33	0	48	2.1e-05	1	U	I.GENGPHLR.V
2683	725 - 741	908.4589	1814.9033	1814.9051	-0.98	0	33	0.0007	1	U	R.VLHVDDLSSLQVFEGAS.T
2971	725 - 743	691.6913	2072.0522	2072.0538	-0.81	0	88	1.6e-09	1	U	R.VLHVDDLSSLQVFEGASTR.T

Query	Start – End	Observed	Mr(expt)	Mr(calc)	ppm	M	Score	Expect	Rank	U	Peptide
2972	725 – 743	1037.0335	2072.0524	2072.0538	-0.70	0	123	5e-13	1	U	R.VLHVDDLSSLQVFEGASTR.T
2973	725 – 743	691.6915	2072.0526	2072.0538	-0.62	0	101	7.6e-11	1	U	R.VLHVDDLSSLQVFEGASTR.T
2974	725 – 743	691.6916	2072.0529	2072.0538	-0.44	0	53	4.6e-06	1	U	R.VLHVDDLSSLQVFEGASTR.T
2975	725 – 743	691.6918	2072.0536	2072.0538	-0.12	0	54	4.3e-06	1	U	R.VLHVDDLSSLQVFEGASTR.T
2976	725 – 743	691.6926	2072.0560	2072.0538	1.04	0	35	0.00032	1	U	R.VLHVDDLSSLQVFEGASTR.T
2580	728 – 743	862.4276	1722.8406	1722.8425	-1.09	0	82	7.2e-09	1	U	H.VDDLSSLQVFEGASTR.T
122	737 – 743	384.1874	766.3603	766.3610	-0.87	0	31	0.00072	1	U	V.FEGASTR.T
1433	757 – 765	608.8060	1215.5975	1215.5964	0.86	0	65	3.8e-07	1	U	R.YPVPYTYWK.K
3162	767 – 787	773.3621	2317.0644	2317.0631	0.56	1	91	1.2e-09	1	U	K.TTKGEGLDYDSTLGEVMEQTK.R + Oxidation (M)
2885	770 – 787	986.4457	1970.8768	1970.8779	-0.55	0	120	1.1e-12	1	U	K.GEGLDYDSTLGEVMEQTK.R
2886	770 – 787	986.4461	1970.8776	1970.8779	-0.13	0	68	1.5e-07	1	U	K.GEGLDYDSTLGEVMEQTK.R
2887	770 – 787	657.9671	1970.8795	1970.8779	0.83	0	102	5.8e-11	1	U	K.GEGLDYDSTLGEVMEQTK.R
2902	770 – 787	994.4423	1986.8700	1986.8728	-1.41	0	79	1.3e-08	1	U	K.GEGLDYDSTLGEVMEQTK.R + Oxidation (M)
2903	770 – 787	994.4431	1986.8717	1986.8728	-0.55	0	134	4.3e-14	1	U	K.GEGLDYDSTLGEVMEQTK.R + Oxidation (M)
2904	770 – 787	994.4434	1986.8723	1986.8728	-0.28	0	49	1.3e-05	1	U	K.GEGLDYDSTLGEVMEQTK.R + Oxidation (M)
2905	770 – 787	663.2990	1986.8752	1986.8728	1.19	0	82	6.7e-09	1	U	K.GEGLDYDSTLGEVMEQTK.R + Oxidation (M)
3030	770 – 788	709.9996	2126.9770	2126.9790	-0.96	1	108	1.6e-11	1	U	K.GEGLDYDSTLGEVMEQTKR.L
3060	770 – 788	715.3320	2142.9741	2142.9739	0.097	1	103	5.1e-11	1	U	K.GEGLDYDSTLGEVMEQTKR.L + Oxidation (M)
3063	770 – 788	1072.4983	2142.9820	2142.9739	3.77	1	81	7.6e-09	1	U	K.GEGLDYDSTLGEVMEQTKR.L + Oxidation (M)
3045	791 – 809	713.0281	2136.0623	2136.0640	-0.79	0	111	8.7e-12	1	U	R.FHAVPVDPDDLTSPWLTAR.R
3046	791 – 809	713.0290	2136.0651	2136.0640	0.52	0	43	7.5e-05	1	U	R.FHAVPVDPDDLTSPWLTAR.R
3047	791 – 809	1069.0404	2136.0663	2136.0640	1.05	0	34	0.00048	1	U	R.FHAVPVDPDDLTSPWLTAR.R
2714	793 – 809	926.9757	1851.9368	1851.9367	0.051	0	140	1.3e-14	1	U	H.AVPVDPDDLTSPWLTAR.R
1892	798 – 809	686.3485	1370.6825	1370.6830	-0.37	0	89	1.4e-09	1	U	D.PDDLTSPWLTAR.R
1306	800 – 809	580.3096	1158.6047	1158.6033	1.20	0	52	7e-06	1	U	D.DLTSPWLTAR.R
953	818 – 826	351.5296	1051.5669	1051.5662	0.69	1	39	0.00012	1	U	R.KVLGTSEYR.A
954	818 – 826	526.7916	1051.5687	1051.5662	2.36	1	80	1e-08	1	U	R.KVLGTSEYR.A
551	819 – 826	462.7407	923.4668	923.4712	-4.85	0	70	1.1e-07	1	U	K.VLGTSEYR.A
17	857 – 863	359.6904	717.3663	717.3657	0.78	0	41	0.00022	1	U	R.NVTEGAK.R
3108	865 – 883	1100.5896	2199.1647	2199.1674	-1.23	0	132	6.5e-14	1	U	R.EVEGITTELEPDLLYPLLR.G
3109	865 – 883	734.0629	2199.1669	2199.1674	-0.25	0	92	5.9e-10	1	U	R.EVEGITTELEPDLLYPLLR.G
3110	865 – 883	1100.5936	2199.1727	2199.1674	2.41	0	31	0.00081	1	U	R.EVEGITTELEPDLLYPLLR.G
816	890 – 897	501.2464	1000.4783	1000.4767	1.66	0	50	9.7e-06	1	U	R.WYAQPSLH.I
2809	890 – 905	642.6697	1924.9872	1924.9869	0.17	0	56	2.7e-06	1	U	R.WYAQPSLHILMVQDPK.T
2840	890 – 905	648.0016	1940.9829	1940.9818	0.55	0	69	1.2e-07	1	U	R.WYAQPSLHILMVQDPK.T + Oxidation (M)
659	898 – 905	480.2651	958.5157	958.5157	-0.030	0	67	2.4e-07	1	U	H.ILMVQDPK.T + Oxidation (M)
1355	908 – 917	395.8907	1184.6504	1184.6513	-0.79	1	54	4.7e-06	1	U	R.RGIDEQVLQK.R
1356	908 – 917	593.3338	1184.6531	1184.6513	1.47	1	59	1.8e-06	1	U	R.RGIDEQVLQK.R
895	909 – 917	515.2817	1028.5488	1028.5502	-1.35	0	65	3.3e-07	1	U	R.GIDEQVLQK.R
896	909 – 917	515.2829	1028.5512	1028.5502	0.93	0	71	8.4e-08	1	U	R.GIDEQVLQK.R
138	922 – 927	391.2147	780.4148	780.4170	-2.86	0	38	0.00031	1	U	K.TWAYLK.R
139	922 – 927	391.2158	780.4170	780.4170	-0.068	0	32	0.0011	1	U	K.TWAYLK.R
52	929 – 934	367.7118	733.4090	733.4123	-4.43	0	54	4e-06	1	U	R.FEAVLR.E
55	929 – 934	367.7132	733.4118	733.4123	-0.67	0	37	0.0002	1	U	R.FEAVLR.E
56	929 – 934	367.7132	733.4119	733.4123	-0.53	0	37	0.00022	1	U	R.FEAVLR.E
57	929 – 934	367.7137	733.4129	733.4123	0.91	0	30	0.001	1	U	R.FEAVLR.E
1776	952 – 962	670.7795	1339.5445	1339.5465	-1.47	0	37	0.0002	1	U	R.MVETGPFYSMF.N + 2 Oxidation (M)
1666	963 – 973	649.3149	1296.6152	1296.6139	1.03	0	64	4.2e-07	1	U	F.NVGDYTFAPWK.V
2723	978 – 995	622.9877	1865.9412	1865.9411	0.032	0	83	5.1e-09	1	U	R.YVASDFIVAVVGPASDEK.P
2724	978 – 995	933.9784	1865.9423	1865.9411	0.63	0	159	1.2e-16	1	U	R.YVASDFIVAVVGPASDEK.P
3183	978 – 1000	1187.1203	2372.2261	2372.2264	-0.12	0	49	1.3e-05	1	U	R.YVASDFIVAVVGPASDEKPVVPN.E
3184	978 – 1000	791.7495	2372.2268	2372.2264	0.15	0	89	1.3e-09	1	U	R.YVASDFIVAVVGPASDEKPVVPN.E
3225	978 – 1002	877.4582	2629.3528	2629.3639	-4.23	0	34	0.00039	1	U	R.YVASDFIVAVVGPASDEKPVVPNEK.L
3226	978 – 1002	877.4599	2629.3578	2629.3639	-2.32	0	79	1.2e-08	1	U	R.YVASDFIVAVVGPASDEKPVVPNEK.L
3227	978 – 1002	877.4613	2629.3620	2629.3639	-0.74	0	132	6.1e-14	1	U	R.YVASDFIVAVVGPASDEKPVVPNEK.L
3228	978 – 1002	658.3494	2629.3686	2629.3639	1.76	0	85	3.2e-09	1	U	R.YVASDFIVAVVGPASDEKPVVPNEK.L
3229	978 – 1002	877.4635	2629.3687	2629.3639	1.82	0	72	6.5e-08	1	U	R.YVASDFIVAVVGPASDEKPVVPNEK.L
3230	978 – 1002	877.4636	2629.3688	2629.3639	1.86	0	86	2.6e-09	1	U	R.YVASDFIVAVVGPASDEKPVVPNEK.L
140	996 – 1002	391.7239	781.4333	781.4334	-0.091	0	55	2.9e-06	1	U	K.PVVPNEK.L
1338	1028 – 1037	587.3016	1172.5887	1172.5866	1.76	0	35	0.00042	1	U	R.FAVQSFFVQT.Q
1676	1028 – 1038	651.3298	1300.6451	1300.6452	-0.13	0	44	3.8e-05	1	U	R.FAVQSFFVQTQ.I
2577	1028 – 1042	860.4453	1718.8761	1718.8781	-1.14	0	114	6e-12	1	U	R.FAVQSFFVQTQIAPH.V
2579	1028 – 1042	573.9668	1718.8785	1718.8781	0.27	0	103	6.9e-11	1	U	R.FAVQSFFVQTQIAPH.V
3100	1028 – 1046	730.0691	2187.1853	2187.1841	0.57	0	99	1.3e-10	1	U	R.FAVQSFFVQTQIAPHVLQK.L
2286	1034 – 1046	754.9336	1507.8527	1507.8511	1.07	0	69	1.2e-07	1	U	F.FVQTQIAPHVLQK.L
2287	1034 – 1046	503.6250	1507.8533	1507.8511	1.44	0	57	2e-06	1	U	F.FVQTQIAPHVLQK.L
1225	1037 – 1046	378.8928	1133.6565	1133.6557	0.71	0	41	7.2e-05	1	U	Q.TQIAPHVLQK.L
496	1039 – 1046	302.5238	904.5496	904.5494	0.21	0	43	5e-05	1	U	Q.IAPHVLQK.L
1156	1052 – 1060	559.2310	1116.4475	1116.4472	0.30	0	32	0.00068	1	U	R.YEPNTDHQN.R
1608	1052 – 1061	425.1898	1272.5474	1272.5483	-0.69	0	55	3.1e-06	1	U	R.YEPNTDHQNR.I
1609	1052 – 1061	637.2822	1272.5498	1272.5483	1.16	0	82	7.1e-09	1	U	R.YEPNTDHQNR.I
737	1054 – 1061	491.2286	980.4426	980.4424	0.20	0	55	5.2e-06	1	U	E.PNTDHQNR.I
1830	1069 – 1081	452.8893	1355.6462	1355.6469	-0.56	0	91	7.9e-10	1	U	R.AHELAPAAYNGDK.A

Query	Start - End	Observed	Mr(expt)	Mr(calc)	ppm	M	Score	Expect	Rank	U	Peptide
1831	1069 - 1081	678.8311	1355.6477	1355.6469	0.55	0	106	2.3e-11	1	U	R.AHELAPAAYNGDK.A
927	1089 - 1096	523.2670	1044.5195	1044.5200	-0.42	1	60	1.1e-06	1	U	R.RVEEEIDR.A
928	1089 - 1096	349.1807	1044.5202	1044.5200	0.23	1	47	2.5e-05	1	U	R.RVEEEIDR.A
461	1090 - 1096	445.2158	888.4171	888.4189	-2.01	0	55	3e-06	1	U	R.VEEEIDR.A
2755	1097 - 1113	950.4933	1898.9721	1898.9737	-0.88	0	105	3.1e-11	1	U	R.AAAQLWGLTEEELAEIR.R
2756	1097 - 1113	633.9984	1898.9732	1898.9737	-0.27	0	131	8.3e-14	1	U	R.AAAQLWGLTEEELAEIR.R
2962	1097 - 1114	686.0328	2055.0766	2055.0748	0.83	1	68	1.6e-07	1	U	R.AAAQLWGLTEEELAEIRR.S
1566	1103 - 1113	630.3270	1258.6394	1258.6405	-0.84	0	69	1.1e-07	1	U	W.GLTEEELAEIR.R
199	1115 - 1121	402.2158	802.4170	802.4185	-1.86	1	42	0.00015	1	U	R.SLEELRG.-
200	1115 - 1121	402.2160	802.4174	802.4185	-1.34	1	42	0.00016	1	U	R.SLEELRG.-

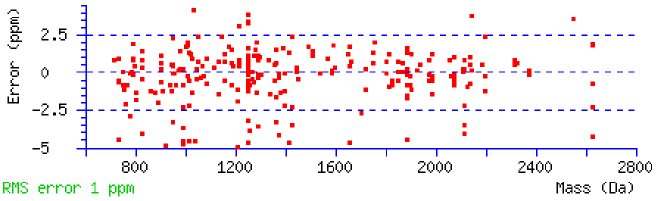

Mascot: http://www.matrixscience.com/
