# Supplementary material for: Thermostable proteins bioprocesses: The activity of restriction endonuclease-methyltransferase from Thermus thermophilus (RM.TthHB27I) cloned in Escherichia coli is critically affected by the codon composition of the synthetic gene
Source: PLoS One. 2017 Oct 17;12(10):e0186633. doi: 10.1371/journal.pone.0186633 (PMC5645126; doi:10.1371/journal.pone.0186633)
Supplement: S5 File — (PDF) [file pone.0186633.s005.pdf]

MASCOT Search Results

Protein View: 366

RM.TthHB27I [agafab 20160615]

**Database:**	small-www
**Score:**	16166
**Nominal mass (M_r):**	127974
**Calculated pI:**	6.77

Sequence similarity is available as an NCBI BLAST search of 366 against nr.

Search parameters

**MS data file:**	\\212.87.29.243\Dane\USERS\Agata\tmp\60614971kref_syn_TthHB27I.mgf
**Enzyme:**	semiTrypsin: cuts C-term side of KR unless next residue is P.<br>Cleavage is semi-specific. (Peptide can be non-specific at one terminus only.)
**Fixed modifications:**	Carbamidomethyl (C)
**Variable modifications:**	Oxidation (M)

Protein sequence coverage: 73%

Matched peptides shown in ***bold red***.

1	MLSLLTGGVF	RRVKLMNWID	LYTHLKQEVP	WFFNSVRLAA	SQAHNEAEFE
51	SRINNAIERL	AQKLGVQLLF	REQYTLATGR	ADAVYNRLVI	EYEPPGSLRP
101	NLKHSHTQHA	VRQVMNYIEE	LSRAERHDRD	RLLGVVFDGH	YFIFVRYHEG
151	HWIVEEPLEV	NPASCERFLR	SLFSLSSGRA	LIPENLVEDF	GSQNDLSRQA
201	TRALYHALQG	HTSDLTARLF	VQWQIFFGET	AGADAAGGEL	KHKSELLAFA
251	RGMGLRGSRI	DMPRFLFALH	TYFSFLVKNI	ARLVLQAYAG	GGLGTTPLTT
301	IANLEGEALR	RELQNLESGG	LFRTLGLKNL	LEGDFFAWYL	DAWNPEVEEA
351	LRQVLARLAE	YNPATVQDDP	HSARDLLKKL	YHYLLPRDIR	HDLGEFYTPD
401	WLAERLLNQL	GEPWFIMPPG	NHPPRGLPDK	RLLDPACGSG	TFLVLAIRAL
451	KVNCFLAGFS	EADTLEVILN	SVVGIDLNPL	AVTAARVNYL	LAIADLLPYR
501	RREVEIPVYL	ADSILTPARG	EGLFAQNRRI	LETAVGPLPV	PEVINSRAKM
551	ERLTDLLEEY	VRGDFSTEAF	LARAKKEIPD	LADALHADEV	LTELYERLRD
601	LHRQGLDGIW	ARVLKNAFMP	LFLEPFDYVV	GNPPWINWES	LPQAYREQTA
651	ELWTCYGLFV	HSGMDTILGK	GKKDASTLMT	YAVADRFLKE	GGKLGFLITQ
701	SVWKTGAGQG	FRRFRIGENG	PHLRVLHVDD	LSSLQVFEGA	STRTSAFVLQ
751	KGRPTRYPVP	YTYWKKTTKG	EGLDYDSTLG	EVMEQTKRLR	FHAVPVDPDD
801	LTSPWLTARR	RALYAVRKVL	GTSEYRAYEG	ANSGGANGIY	WLEILAERPD
851	GLVVVRNVTE	GAKREVEGIT	TELEPDLLYP	LLRGRDVRRW	YAQPSLHILM
901	VQDPKTRRGI	DEQVLQKRYP	KTWAYLKRFE	AVLRERSGFR	RYFTRKDRNG
951	RMVETGPFYS	MFNVGDYTFA	PWKVVWRYVA	SDFIVAVVGP	ASDEKPVVPN
1001	EKLMLVPVED	DNEAFYLCGV	LNSSPIRFAV	QSFFVQTQIA	PHVLQKLCIP
1051	RYEPNTDHQN	RIAHLSRRAH	ELAPAAYNGD	KAARAELRRV	EEEIDRAAAQ
1101	LWGLTEEELA	EIRRSLEELR	G		

Unformatted sequence string: **1121 residues** (for pasting into other applications).

Sort peptides by ◉ Residue Number ○ Increasing Mass ○ Decreasing Mass

Query	Start – End	Observed	Mr(expt)	Mr(calc)	ppm	M	Score	Expect	Rank	U	Peptide
1231	1 – 11	597.3391	1192.6637	1192.6638	-0.080	0	100	9.4e-11	1	U	-.MLSLLTGGVFR.R
1273	1 – 11	605.3354	1208.6562	1208.6587	-2.06	0	85	3.9e-09	1	U	-.MLSLLTGGVFR.R + Oxidation (M)
1274	1 – 11	605.3360	1208.6575	1208.6587	-1.00	0	60	1e-06	1	U	-.MLSLLTGGVFR.R + Oxidation (M)
1275	1 – 11	605.3363	1208.6581	1208.6587	-0.52	0	62	6.3e-07	1	U	-.MLSLLTGGVFR.R + Oxidation (M)
889	2 – 11	531.8189	1061.6233	1061.6233	-0.042	0	91	7.4e-10	1	U	M.LSLLTGGVFR.R
519	3 – 11	475.2766	948.5386	948.5393	-0.73	0	70	9e-08	1	U	L.SLLTGGVFR.R
63	5 – 11	375.2185	748.4225	748.4232	-0.95	0	63	9.5e-07	1	U	L.LTGGVFR.R
1665	15 – 24	661.3154	1320.6162	1320.6172	-0.81	0	68	1.6e-07	1	U	K.LMNWIDLYTH.L + Oxidation (M)
2345	15 – 26	516.2747	1545.8022	1545.8013	0.54	0	41	8.6e-05	1	U	K.LMNWIDLYTHLK.Q
2360	15 – 26	781.9038	1561.7930	1561.7963	-2.10	0	71	8.6e-08	1	U	K.LMNWIDLYTHLK.Q + Oxidation (M)
2361	15 – 26	521.6057	1561.7952	1561.7963	-0.66	0	70	1.2e-07	1	U	K.LMNWIDLYTHLK.Q + Oxidation (M)
902	27 – 34	533.7542	1065.4938	1065.4920	1.72	0	32	0.00087	1	U	K.QEVPWFFN.S
2011	27 – 37	704.8543	1407.6941	1407.6935	0.38	0	64	4.6e-07	1	U	K.QEVPWFFNSVR.L
701	38 – 47	506.2470	1010.4794	1010.4781	1.29	0	47	1.9e-05	1	U	R.LAASQAHNEA.E
2479	38 – 52	830.3886	1658.7627	1658.7648	-1.27	0	143	5e-15	1	U	R.LAASQAHNEAEFESR.I
2480	38 – 52	830.3887	1658.7629	1658.7648	-1.15	0	92	6.3e-10	1	U	R.LAASQAHNEAEFESR.I

Query	Start – End	Observed	Mr(expt)	Mr(calc)	ppm	M	Score	Expect	Rank	U	Peptide
2481	38 – 52	553.9288	1658.7646	1658.7648	-0.14	0	75	3.2e-08	1	U	R.LAASQAHNEAEFESR.I
2482	38 – 52	830.3896	1658.7646	1658.7648	-0.12	0	109	1.3e-11	1	U	R.LAASQAHNEAEFESR.I
2483	38 – 52	830.3898	1658.7651	1658.7648	0.16	0	83	4.7e-09	1	U	R.LAASQAHNEAEFESR.I
2484	38 – 52	553.9292	1658.7658	1658.7648	0.58	0	81	8.1e-09	1	U	R.LAASQAHNEAEFESR.I
2486	38 – 52	553.9297	1658.7672	1658.7648	1.47	0	74	3.7e-08	1	U	R.LAASQAHNEAEFESR.I
2487	38 – 52	553.9306	1658.7700	1658.7648	3.15	0	70	9.9e-08	1	U	R.LAASQAHNEAEFESR.I
1646	42 – 52	439.8654	1316.5743	1316.5745	-0.12	0	71	8.9e-08	1	U	S.QAHNEAEFESR.I
1224	43 – 52	595.2653	1188.5160	1188.5159	0.088	0	43	4.8e-05	1	U	Q.AHNEAEFESR.I
614	45 – 52	491.2166	980.4187	980.4199	-1.25	0	57	1.8e-06	1	U	H.NEAEFESR.I
215	53 – 59	415.2296	828.4447	828.4453	-0.77	0	49	1.2e-05	1	U	R.INNAIER.L
216	53 – 59	415.2298	828.4451	828.4453	-0.26	0	44	4.2e-05	1	U	R.INNAIER.L
217	53 – 59	415.2299	828.4453	828.4453	-0.065	0	36	0.00027	1	U	R.INNAIER.L
219	53 – 59	415.2301	828.4456	828.4453	0.30	0	46	2.7e-05	1	U	R.INNAIER.L
220	53 – 59	415.2302	828.4458	828.4453	0.59	0	41	8.4e-05	1	U	R.INNAIER.L
221	53 – 59	415.2302	828.4458	828.4453	0.59	0	39	0.00012	1	U	R.INNAIER.L
10	54 – 59	358.6871	715.3597	715.3613	-2.13	0	35	0.00036	1	U	I.NNAIER.L
498	64 – 71	473.2968	944.5791	944.5807	-1.72	0	70	1e-07	1	U	K.LGVQLLFR.E
499	64 – 71	473.2973	944.5801	944.5807	-0.70	0	51	7.3e-06	1	U	K.LGVQLLFR.E
500	64 – 71	473.2974	944.5802	944.5807	-0.53	0	57	1.9e-06	1	U	K.LGVQLLFR.E
501	64 – 71	473.2974	944.5803	944.5807	-0.43	0	76	2.6e-08	1	U	K.LGVQLLFR.E
502	64 – 71	473.2975	944.5804	944.5807	-0.36	0	76	2.7e-08	1	U	K.LGVQLLFR.E
503	64 – 71	473.2975	944.5804	944.5807	-0.32	0	82	6.4e-09	1	U	K.LGVQLLFR.E
504	64 – 71	473.2975	944.5804	944.5807	-0.32	0	82	6.6e-09	1	U	K.LGVQLLFR.E
505	64 – 71	473.2978	944.5811	944.5807	0.33	0	76	2.8e-08	1	U	K.LGVQLLFR.E
506	64 – 71	473.2978	944.5811	944.5807	0.38	0	82	6.3e-09	1	U	K.LGVQLLFR.E
507	64 – 71	473.2983	944.5821	944.5807	1.46	0	82	6.6e-09	1	U	K.LGVQLLFR.E
102	66 – 71	388.2459	774.4773	774.4752	2.75	0	42	5.7e-05	1	U	G.VQLLFR.E
169	81 – 87	404.7006	807.3866	807.3875	-1.08	0	40	9.9e-05	1	U	R.ADAVYNR.L
170	81 – 87	404.7015	807.3884	807.3875	1.17	0	38	0.00015	1	U	R.ADAVYNR.L
171	81 – 87	404.7019	807.3892	807.3875	2.06	0	36	0.00027	1	U	R.ADAVYNR.L
172	81 – 87	404.7024	807.3902	807.3875	3.30	0	36	0.00027	1	U	R.ADAVYNR.L
1872	88 – 99	686.8766	1371.7387	1371.7398	-0.81	0	83	4.9e-09	1	U	R.LVIEYEPPGSLR.P
2388	88 – 101	792.4239	1582.8332	1582.8355	-1.44	0	80	1.1e-08	1	U	R.LVIEYEPPGSLRPN.L
2389	88 – 101	528.6187	1582.8344	1582.8355	-0.70	0	70	1.1e-07	1	U	R.LVIEYEPPGSLRPN.L
2705	88 – 103	609.0120	1824.0141	1824.0145	-0.21	0	77	2.1e-08	1	U	R.LVIEYEPPGSLRPNLK.H
1902	113 – 123	691.3398	1380.6651	1380.6707	-4.05	0	87	2.8e-09	1	U	R.QVMNYIEELSR.A
1963	113 – 123	699.3369	1396.6593	1396.6656	-4.55	0	56	4.3e-06	1	U	R.QVMNYIEELSR.A + Oxidation (M)
1964	113 – 123	699.3375	1396.6604	1396.6656	-3.72	0	49	1.7e-05	1	U	R.QVMNYIEELSR.A + Oxidation (M)
1965	113 – 123	699.3384	1396.6623	1396.6656	-2.36	0	42	5.9e-05	1	U	R.QVMNYIEELSR.A + Oxidation (M)
1967	113 – 123	699.3387	1396.6629	1396.6656	-1.96	0	31	0.00088	1	U	R.QVMNYIEELSR.A + Oxidation (M)
1971	113 – 123	699.3408	1396.6670	1396.6656	0.99	0	46	2.6e-05	1	U	R.QVMNYIEELSR.A + Oxidation (M)
1972	113 – 123	699.3419	1396.6692	1396.6656	2.55	0	53	5.1e-06	1	U	R.QVMNYIEELSR.A + Oxidation (M)
1973	113 – 123	699.3422	1396.6698	1396.6656	3.01	0	68	1.6e-07	1	U	R.QVMNYIEELSR.A + Oxidation (M)
753	116 – 123	512.2592	1022.5038	1022.5032	0.52	0	54	4.2e-06	1	U	M.NYIEELSR.A
3368	147 – 167	638.5485	2550.1648	2550.1597	1.99	0	39	0.00012	1	U	R.YHEGHWIVEEPLEVNPASCER.F
2902	152 – 167	964.4643	1926.9141	1926.9145	-0.21	0	128	1.6e-13	1	U	H.WIVEEPLEVNPASCER.F
533	171 – 179	477.2561	952.4977	952.4978	-0.077	0	65	3.4e-07	1	U	R.SLFSLSSGR.A
534	171 – 179	477.2562	952.4978	952.4978	0.049	0	52	6.7e-06	1	U	R.SLFSLSSGR.A
535	171 – 179	477.2562	952.4979	952.4978	0.15	0	72	7.3e-08	1	U	R.SLFSLSSGR.A
536	171 – 179	477.2568	952.4991	952.4978	1.37	0	44	4.5e-05	1	U	R.SLFSLSSGR.A
537	171 – 179	477.2569	952.4993	952.4978	1.58	0	68	1.5e-07	1	U	R.SLFSLSSGR.A
538	171 – 179	477.2571	952.4997	952.4978	1.96	0	51	9.1e-06	1	U	R.SLFSLSSGR.A
539	171 – 179	477.2572	952.4999	952.4978	2.19	0	48	1.7e-05	1	U	R.SLFSLSSGR.A
3142	180 – 198	706.3526	2116.0360	2116.0436	-3.61	0	99	1.3e-10	1	U	R.ALIPENLVEDFGSQNDLSR.Q
3145	180 – 198	706.3536	2116.0390	2116.0436	-2.21	0	34	0.0004	1	U	R.ALIPENLVEDFGSQNDLSR.Q
3147	180 – 198	1059.0274	2116.0402	2116.0436	-1.61	0	148	1.6e-15	1	U	R.ALIPENLVEDFGSQNDLSR.Q
3148	180 – 198	1059.0293	2116.0440	2116.0436	0.19	0	133	4.8e-14	1	U	R.ALIPENLVEDFGSQNDLSR.Q
2402	185 – 198	797.3789	1592.7432	1592.7431	0.063	0	92	5.8e-10	1	U	E.NLVEDFGSQNDLSR.Q
2232	186 – 198	740.3568	1478.6991	1478.7001	-0.70	0	112	8.3e-12	1	U	N.LVEDFGSQNDLSR.Q
695	203 – 211	337.1785	1008.5137	1008.5141	-0.37	0	71	8.4e-08	1	U	R.ALYHALQGH.T
696	203 – 211	505.2653	1008.5161	1008.5141	1.94	0	74	3.6e-08	1	U	R.ALYHALQGH.T
2626	203 – 218	439.2286	1752.8852	1752.8907	-3.14	0	45	3.5e-05	1	U	R.ALYHALQGHTSDLTAR.L
2627	203 – 218	877.4508	1752.8870	1752.8907	-2.14	0	119	1.3e-12	1	U	R.ALYHALQGHTSDLTAR.L
2628	203 – 218	585.3030	1752.8871	1752.8907	-2.05	0	93	5.3e-10	1	U	R.ALYHALQGHTSDLTAR.L
2629	203 – 218	585.3033	1752.8880	1752.8907	-1.55	0	36	0.00028	1	U	R.ALYHALQGHTSDLTAR.L
2631	203 – 218	585.3044	1752.8914	1752.8907	0.38	0	33	0.00052	1	U	R.ALYHALQGHTSDLTAR.L
2001	206 – 218	469.5762	1405.7069	1405.7062	0.49	0	78	1.6e-08	1	U	Y.HALQGHTSDLTAR.L
956	209 – 218	362.5162	1084.5267	1084.5261	0.54	0	56	2.3e-06	1	U	L.QGHTSDLTAR.L
87	212 – 218	382.2008	762.3870	762.3872	-0.24	0	68	1.8e-07	1	U	H.TSDLTAR.L
2660	224 – 241	891.4378	1780.8611	1780.8632	-1.19	0	120	1.1e-12	1	U	W.QIFFGETAGADAAGGELK.H
2470	225 – 241	827.4095	1652.8045	1652.8046	-0.062	0	127	2.2e-13	1	U	Q.IFFGETAGADAAGGELK.H
2471	225 – 241	551.9427	1652.8063	1652.8046	1.04	0	59	1.5e-06	1	U	Q.IFFGETAGADAAGGELK.H
1938	227 – 241	697.3333	1392.6520	1392.6521	-0.088	0	102	6.4e-11	1	U	F.FGETAGADAAGGELK.H
1196	242 – 251	391.2243	1170.6511	1170.6509	0.18	1	52	5.8e-06	1	U	K.HKSELLAFAR.G
415	244 – 251	453.7562	905.4978	905.4970	0.79	0	45	3e-05	1	U	K.SELLAFAR.G

Query	Start - End	Observed	Mr(expt)	Mr(calc)	ppm	M	Score	Expect	Rank	U	Peptide
59	265 - 270	374.2129	746.4112	746.4115	-0.46	0	46	4.5e-05	1	U	R.FLFALH.T
2329	283 - 298	765.9294	1529.8442	1529.8454	-0.77	0	36	0.00026	1	U	R.LVLQAYAGGGLGTTPL.T
3130	289 - 310	1056.5660	2111.1175	2111.1222	-2.24	0	66	2.5e-07	1	U	Y.AGGGLGTTPLTTIANLEGEALR.R
3131	289 - 310	704.7151	2111.1235	2111.1222	0.60	0	95	3.4e-10	1	U	Y.AGGGLGTTPLTTIANLEGEALR.R
3132	289 - 310	1056.5695	2111.1245	2111.1222	1.07	0	154	4.2e-16	1	U	Y.AGGGLGTTPLTTIANLEGEALR.R
2633	294 - 310	878.9725	1755.9305	1755.9367	-3.50	0	92	5.9e-10	1	U	L.GTTPLTTIANLEGEALR.R
1555	299 - 310	644.3475	1286.6805	1286.6830	-1.93	0	99	1.3e-10	1	U	L.TTIANLEGEALR.R
2146	299 - 311	722.4000	1442.7854	1442.7841	0.88	1	31	0.00082	1	U	L.TTIANLEGEALRR.E
2147	299 - 311	481.9358	1442.7856	1442.7841	1.03	1	55	3.3e-06	1	U	L.TTIANLEGEALRR.E
595	302 - 310	486.7593	971.5041	971.5036	0.51	0	35	0.00035	1	U	I.ANLEGEALR.R
394	303 - 310	451.2404	900.4662	900.4665	-0.34	0	52	7.3e-06	1	U	A.NLEGEALR.R
114	304 - 310	394.2191	786.4236	786.4235	0.14	0	60	3e-06	1	U	N.LEGEALR.R
115	304 - 310	394.2194	786.4242	786.4235	0.78	0	33	0.0017	1	U	N.LEGEALR.R
116	304 - 310	394.2194	786.4242	786.4235	0.88	0	55	9.3e-06	1	U	N.LEGEALR.R
2312	311 - 323	759.9032	1517.7918	1517.7950	-2.11	1	66	3.3e-07	1	U	R.RELQNLESGGLFR.T
2313	311 - 323	506.9388	1517.7947	1517.7950	-0.20	1	75	4.7e-08	1	U	R.RELQNLESGGLFR.T
1821	312 - 323	681.8512	1361.6879	1361.6939	-4.42	0	43	7.4e-05	1	U	R.ELQNLESGGLFR.T
1822	312 - 323	681.8513	1361.6881	1361.6939	-4.23	0	34	0.0006	1	U	R.ELQNLESGGLFR.T
1823	312 - 323	681.8517	1361.6888	1361.6939	-3.73	0	53	7e-06	1	U	R.ELQNLESGGLFR.T
1824	312 - 323	681.8519	1361.6893	1361.6939	-3.40	0	75	4.6e-08	1	U	R.ELQNLESGGLFR.T
1826	312 - 323	681.8521	1361.6896	1361.6939	-3.16	0	31	0.00075	1	U	R.ELQNLESGGLFR.T
1827	312 - 323	681.8523	1361.6900	1361.6939	-2.85	0	51	8.8e-06	1	U	R.ELQNLESGGLFR.T
1829	312 - 323	681.8525	1361.6905	1361.6939	-2.47	0	35	0.00034	1	U	R.ELQNLESGGLFR.T
1832	312 - 323	681.8533	1361.6920	1361.6939	-1.38	0	35	0.00039	1	U	R.ELQNLESGGLFR.T
1833	312 - 323	681.8535	1361.6924	1361.6939	-1.12	0	58	1.8e-06	1	U	R.ELQNLESGGLFR.T
1834	312 - 323	681.8535	1361.6924	1361.6939	-1.11	0	33	0.00065	1	U	R.ELQNLESGGLFR.T
1835	312 - 323	681.8536	1361.6927	1361.6939	-0.88	0	35	0.00036	1	U	R.ELQNLESGGLFR.T
1836	312 - 323	681.8536	1361.6927	1361.6939	-0.86	0	48	1.8e-05	1	U	R.ELQNLESGGLFR.T
1837	312 - 323	681.8537	1361.6929	1361.6939	-0.75	0	66	2.4e-07	1	U	R.ELQNLESGGLFR.T
1839	312 - 323	681.8539	1361.6933	1361.6939	-0.46	0	31	0.00092	1	U	R.ELQNLESGGLFR.T
1841	312 - 323	681.8541	1361.6937	1361.6939	-0.18	0	37	0.0002	1	U	R.ELQNLESGGLFR.T
1842	312 - 323	681.8543	1361.6941	1361.6939	0.17	0	69	1.3e-07	1	U	R.ELQNLESGGLFR.T
1843	312 - 323	681.8546	1361.6947	1361.6939	0.60	0	74	5e-08	1	U	R.ELQNLESGGLFR.T
1844	312 - 323	681.8548	1361.6950	1361.6939	0.77	0	47	2.6e-05	1	U	R.ELQNLESGGLFR.T
1845	312 - 323	681.8549	1361.6953	1361.6939	1.00	0	46	3.1e-05	1	U	R.ELQNLESGGLFR.T
649	315 - 323	496.7618	991.5090	991.5087	0.37	0	69	1.2e-07	1	U	Q.NLESGGLFR.T
352	316 - 323	439.7400	877.4655	877.4658	-0.33	0	68	4.9e-07	1	U	N.LESGGLFR.T
2550	339 - 352	852.9130	1703.8114	1703.8155	-2.37	0	104	4.4e-11	1	U	W.YLDAWNPEVEEALR.Q
864	344 - 352	528.7687	1055.5228	1055.5247	-1.80	0	49	1.3e-05	1	U	W.NPEVEEALR.Q
1710	358 - 369	668.3065	1334.5984	1334.5990	-0.44	0	46	2.3e-05	1	U	R.LAEYNPATVQDD.P
2367	358 - 371	785.3616	1568.7086	1568.7107	-1.33	0	93	5.5e-10	1	U	R.LAEYNPATVQDDPH.S
2789	358 - 374	942.4452	1882.8758	1882.8809	-2.73	0	111	8.2e-12	1	U	R.LAEYNPATVQDDPHSAR.D
2791	358 - 374	471.7270	1882.8790	1882.8809	-1.04	0	32	0.00062	1	U	R.LAEYNPATVQDDPHSAR.D
2792	358 - 374	628.6340	1882.8803	1882.8809	-0.35	0	67	2e-07	1	U	R.LAEYNPATVQDDPHSAR.D
2793	358 - 374	628.6340	1882.8803	1882.8809	-0.33	0	58	1.7e-06	1	U	R.LAEYNPATVQDDPHSAR.D
2794	358 - 374	628.6347	1882.8824	1882.8809	0.77	0	106	2.5e-11	1	U	R.LAEYNPATVQDDPHSAR.D
2795	358 - 374	942.4496	1882.8847	1882.8809	2.01	0	37	0.0002	1	U	R.LAEYNPATVQDDPHSAR.D
2796	358 - 374	628.6364	1882.8872	1882.8809	3.35	0	41	8e-05	1	U	R.LAEYNPATVQDDPHSAR.D
1580	363 - 374	431.8778	1292.6116	1292.6109	0.56	0	64	4.9e-07	1	U	N.PATVQDDPHSAR.D
1079	365 - 374	375.8480	1124.5221	1124.5211	0.96	0	48	1.6e-05	1	U	A.TVQDDPHSAR.D
1080	365 - 374	563.2686	1124.5226	1124.5211	1.41	0	46	2.5e-05	1	U	A.TVQDDPHSAR.D
1258	379 - 387	401.5731	1201.6975	1201.6971	0.30	1	43	5.5e-05	1	U	K.KLYHYLLPR.D
923	380 - 387	358.8742	1073.6008	1073.6022	-1.26	0	34	0.00057	1	U	K.LYHYLLPR.D
924	380 - 387	358.8747	1073.6024	1073.6022	0.22	0	49	1.2e-05	1	U	K.LYHYLLPR.D
1229	391 - 400	597.2600	1192.5055	1192.5037	1.52	0	53	5.1e-06	1	U	R.HDLGEFYTPD.W
2732	391 - 405	616.9549	1847.8429	1847.8479	-2.71	0	84	3.9e-09	1	U	R.HDLGEFYTPDWLAER.L
2733	391 - 405	924.9296	1847.8446	1847.8479	-1.74	0	142	5.7e-15	1	U	R.HDLGEFYTPDWLAER.L
2734	391 - 405	616.9578	1847.8517	1847.8479	2.07	0	59	1.3e-06	1	U	R.HDLGEFYTPDWLAER.L
3329	406 - 425	777.0679	2328.1819	2328.1837	-0.79	0	65	3.2e-07	1	U	R.LLNQLGEPWFIMPPGNHPPR.G + Oxidation (M)
1532	415 - 425	426.8858	1277.6356	1277.6339	1.31	0	61	8.2e-07	1	U	W.FIMPPGNHPPR.G + Oxidation (M)
2681	432 - 448	901.9923	1801.9701	1801.9760	-3.31	0	94	3.8e-10	1	U	R.LLDPACGSGTFLVLAIR.A
2682	432 - 448	901.9941	1801.9736	1801.9760	-1.34	0	139	1.4e-14	1	U	R.LLDPACGSGTFLVLAIR.A
2683	432 - 448	601.6663	1801.9772	1801.9760	0.63	0	120	9.2e-13	1	U	R.LLDPACGSGTFLVLAIR.A
1581	437 - 448	647.3524	1292.6903	1292.6911	-0.61	0	114	4.1e-12	1	U	A.CGSGTFLVLAIR.A
1105	438 - 448	567.3372	1132.6599	1132.6604	-0.44	0	75	3e-08	1	U	C.GSGTFLVLAIR.A
635	440 - 448	495.3116	988.6086	988.6069	1.67	0	48	1.7e-05	1	U	S.GTFLVLAIR.A
2404	471 - 486	798.4573	1594.9001	1594.9043	-2.58	0	106	2.4e-11	1	U	N.SVVGIDLNPLAVTAAR.V
2406	471 - 486	532.6426	1594.9059	1594.9043	1.06	0	79	1.4e-08	1	U	N.SVVGIDLNPLAVTAAR.V
2286	472 - 486	754.9428	1507.8710	1507.8722	-0.84	0	53	4.6e-06	1	U	S.VVGIDLNPLAVTAAR.V
1450	475 - 486	627.3642	1252.7138	1252.7139	-0.13	0	83	5.4e-09	1	U	G.IDLNPLAVTAAR.V
762	477 - 486	513.3089	1024.6033	1024.6029	0.41	0	48	1.6e-05	1	U	D.LNPLAVTAAR.V
793	492 - 500	516.2980	1030.5814	1030.5811	0.26	0	64	4.6e-07	1	U	L.AIADLLPYR.R
557	493 - 500	480.7791	959.5437	959.5440	-0.27	0	63	5.8e-07	1	U	A.IADLLPYR.R
1611	502 - 512	652.3492	1302.6838	1302.6819	1.43	1	50	1.3e-05	1	U	R.REVEIPVYLAD.S
3049	502 - 519	681.3804	2041.1193	2041.1208	-0.72	1	115	3.5e-12	1	U	R.REVEIPVYLADSILTPAR.G
3050	502 - 519	681.3809	2041.1208	2041.1208	0.0029	1	38	0.00016	1	U	R.REVEIPVYLADSILTPAR.G
1134	503 - 512	574.2975	1146.5805	1146.5808	-0.32	0	54	4.4e-06	1	U	R.EVEIPVYLAD.S
2803	503 - 519	943.5142	1885.0139	1885.0197	-3.07	0	47	2.1e-05	1	U	R.EVEIPVYLADSILTPAR.G
2804	503 - 519	943.5152	1885.0158	1885.0197	-2.03	0	109	1.3e-11	1	U	R.EVEIPVYLADSILTPAR.G
2806	503 - 519	629.3463	1885.0170	1885.0197	-1.42	0	39	0.00014	1	U	R.EVEIPVYLADSILTPAR.G

Query	Start - End	Observed	Mr(expt)	Mr(calc)	ppm	M	Score	Expect	Rank	U	Peptide
2807	503 - 519	629.3463	1885.0170	1885.0197	-1.40	0	67	2.1e-07	1	U	R.EVEIPVYLADSILTPAR.G
2808	503 - 519	943.5158	1885.0171	1885.0197	-1.36	0	108	1.7e-11	1	U	R.EVEIPVYLADSILTPAR.G
2809	503 - 519	629.3471	1885.0195	1885.0197	-0.082	0	106	2.5e-11	1	U	R.EVEIPVYLADSILTPAR.G
2810	503 - 519	629.3473	1885.0202	1885.0197	0.28	0	102	7e-11	1	U	R.EVEIPVYLADSILTPAR.G
2811	503 - 519	943.5213	1885.0281	1885.0197	4.51	0	48	1.6e-05	1	U	R.EVEIPVYLADSILTPAR.G
75	513 - 519	379.2319	756.4493	756.4494	-0.038	0	44	5.4e-05	1	U	D.SILTPAR.G
641	520 - 528	496.2496	990.4847	990.4883	-3.64	0	34	0.0006	1	U	R.GEGLFAQNR.R
643	520 - 528	496.2513	990.4881	990.4883	-0.15	0	50	1.3e-05	1	U	R.GEGLFAQNR.R
644	520 - 528	496.2526	990.4907	990.4883	2.42	0	42	8e-05	1	U	R.GEGLFAQNR.R
466	521 - 528	467.7401	933.4657	933.4668	-1.23	0	39	0.00013	1	U	G.EGLFAQNR.R
2700	529 - 545	909.0303	1816.0461	1816.0458	0.13	1	108	1.5e-11	1	U	R.RILETAVGPLPVPEVIN.S
3070	529 - 547	687.4001	2059.1784	2059.1790	-0.27	1	74	3.6e-08	1	U	R.RILETAVGPLPVPEVINSR.A
2495	530 - 545	830.9791	1659.9437	1659.9447	-0.61	0	80	1e-08	1	U	R.ILETAVGPLPVPEVIN.S
2844	530 - 547	635.3646	1903.0721	1903.0778	-3.02	0	113	4.7e-12	1	U	R.ILETAVGPLPVPEVINSR.A
2845	530 - 547	952.5451	1903.0757	1903.0778	-1.11	0	94	4e-10	1	U	R.ILETAVGPLPVPEVINSR.A
2161	534 - 547	724.4157	1446.8168	1446.8195	-1.84	0	101	8.9e-11	1	U	T.AVGPLPVPEVINSR.A
1411	553 - 562	625.8323	1249.6500	1249.6554	-4.31	0	75	3.5e-08	1	U	R.LTDLLEEYVR.G
1412	553 - 562	625.8323	1249.6500	1249.6554	-4.31	0	48	1.7e-05	1	U	R.LTDLLEEYVR.G
1413	553 - 562	625.8324	1249.6502	1249.6554	-4.20	0	65	3.7e-07	1	U	R.LTDLLEEYVR.G
1414	553 - 562	625.8332	1249.6519	1249.6554	-2.82	0	70	1.3e-07	1	U	R.LTDLLEEYVR.G
1415	553 - 562	625.8332	1249.6519	1249.6554	-2.79	0	36	0.00029	1	U	R.LTDLLEEYVR.G
1416	553 - 562	625.8333	1249.6520	1249.6554	-2.74	0	70	1.3e-07	1	U	R.LTDLLEEYVR.G
1417	553 - 562	625.8333	1249.6520	1249.6554	-2.69	0	75	3.5e-08	1	U	R.LTDLLEEYVR.G
1418	553 - 562	625.8333	1249.6521	1249.6554	-2.66	0	60	1.3e-06	1	U	R.LTDLLEEYVR.G
1419	553 - 562	625.8334	1249.6523	1249.6554	-2.45	0	90	1.3e-09	1	U	R.LTDLLEEYVR.G
1420	553 - 562	625.8335	1249.6524	1249.6554	-2.44	0	69	1.4e-07	1	U	R.LTDLLEEYVR.G
1421	553 - 562	625.8335	1249.6525	1249.6554	-2.36	0	69	1.4e-07	1	U	R.LTDLLEEYVR.G
1422	553 - 562	625.8339	1249.6533	1249.6554	-1.65	0	64	4.9e-07	1	U	R.LTDLLEEYVR.G
1423	553 - 562	625.8340	1249.6535	1249.6554	-1.49	0	69	1.4e-07	1	U	R.LTDLLEEYVR.G
1424	553 - 562	625.8341	1249.6536	1249.6554	-1.48	0	62	7.1e-07	1	U	R.LTDLLEEYVR.G
1425	553 - 562	625.8342	1249.6538	1249.6554	-1.30	0	82	7.9e-09	1	U	R.LTDLLEEYVR.G
1426	553 - 562	625.8342	1249.6539	1249.6554	-1.20	0	70	1.4e-07	1	U	R.LTDLLEEYVR.G
1427	553 - 562	625.8343	1249.6540	1249.6554	-1.14	0	52	9e-06	1	U	R.LTDLLEEYVR.G
1428	553 - 562	625.8345	1249.6544	1249.6554	-0.82	0	76	3.5e-08	1	U	R.LTDLLEEYVR.G
1429	553 - 562	625.8347	1249.6548	1249.6554	-0.52	0	80	1.4e-08	1	U	R.LTDLLEEYVR.G
1430	553 - 562	625.8347	1249.6549	1249.6554	-0.40	0	70	1.5e-07	1	U	R.LTDLLEEYVR.G
1431	553 - 562	625.8348	1249.6550	1249.6554	-0.34	0	75	3.9e-08	1	U	R.LTDLLEEYVR.G
1432	553 - 562	625.8348	1249.6551	1249.6554	-0.24	0	85	4.8e-09	1	U	R.LTDLLEEYVR.G
1433	553 - 562	625.8352	1249.6558	1249.6554	0.35	0	75	2.9e-08	1	U	R.LTDLLEEYVR.G
1434	553 - 562	625.8352	1249.6559	1249.6554	0.36	0	36	0.00023	1	U	R.LTDLLEEYVR.G
1435	553 - 562	625.8353	1249.6561	1249.6554	0.59	0	69	1.3e-07	1	U	R.LTDLLEEYVR.G
1436	553 - 562	625.8357	1249.6569	1249.6554	1.18	0	73	5.2e-08	1	U	R.LTDLLEEYVR.G
1437	553 - 562	625.8358	1249.6570	1249.6554	1.29	0	37	0.00019	1	U	R.LTDLLEEYVR.G
1438	553 - 562	625.8362	1249.6578	1249.6554	1.95	0	69	1.2e-07	1	U	R.LTDLLEEYVR.G
1439	553 - 562	625.8379	1249.6613	1249.6554	4.73	0	75	4.2e-08	1	U	R.LTDLLEEYVR.G
1111	554 - 562	569.2943	1136.5740	1136.5713	2.37	0	62	6.7e-07	1	U	L.TDLLEEYVR.G
1289	563 - 573	607.2941	1212.5736	1212.5775	-3.25	0	35	0.00035	1	U	R.GDFSTEAFLAR.A
1291	563 - 573	607.2962	1212.5779	1212.5775	0.34	0	86	2.6e-09	1	U	R.GDFSTEAFLAR.A
1293	563 - 573	607.2965	1212.5784	1212.5775	0.75	0	91	8.4e-10	1	U	R.GDFSTEAFLAR.A
1294	563 - 573	607.2966	1212.5786	1212.5775	0.94	0	80	9.5e-09	1	U	R.GDFSTEAFLAR.A
1295	563 - 573	607.2966	1212.5787	1212.5775	0.99	0	92	5.8e-10	1	U	R.GDFSTEAFLAR.A
1296	563 - 573	607.2969	1212.5792	1212.5775	1.38	0	40	0.00011	1	U	R.GDFSTEAFLAR.A
1297	563 - 573	607.2972	1212.5799	1212.5775	1.96	0	71	7.7e-08	1	U	R.GDFSTEAFLAR.A
1298	563 - 573	607.2974	1212.5802	1212.5775	2.22	0	77	2e-08	1	U	R.GDFSTEAFLAR.A
1157	564 - 573	578.7866	1155.5587	1155.5560	2.33	0	54	4.2e-06	1	U	G.DFSTEAFLAR.A
1533	577 - 588	640.3125	1278.6104	1278.6092	0.92	0	45	3e-05	1	U	K.EIPDLADALHAD.E
2477	584 - 597	553.6184	1657.8334	1657.8311	1.35	0	46	3e-05	1	U	D.ALHADEVLTELYER.L
177	598 - 603	405.2408	808.4670	808.4668	0.35	1	36	0.00028	1	U	R.LRDLHR.Q
721	604 - 612	508.2681	1014.5216	1014.5247	-3.02	0	70	1e-07	1	U	R.QGLDGIWAR.V
722	604 - 612	508.2701	1014.5256	1014.5247	0.92	0	69	1.3e-07	1	U	R.QGLDGIWAR.V
723	604 - 612	508.2703	1014.5260	1014.5247	1.35	0	69	1.4e-07	1	U	R.QGLDGIWAR.V
724	604 - 612	508.2709	1014.5272	1014.5247	2.52	0	71	7.2e-08	1	U	R.QGLDGIWAR.V
1728	616 - 626	671.3308	1340.6471	1340.6475	-0.33	0	30	0.00092	1	U	K.NAFMPLFLEPF.D + Oxidation (M)
235	640 - 646	417.7278	833.4411	833.4395	1.83	0	38	0.0002	1	U	E.SLPQAYR.E
2253	657 - 670	497.5941	1489.7604	1489.7599	0.35	0	65	4.9e-07	1	U	Y.GLFVHSGMDTILGK.G + Oxidation (M)
1662	659 - 670	440.8923	1319.6550	1319.6544	0.49	0	72	5.8e-08	1	U	L.FVHSGMDTILGK.G + Oxidation (M)
475	662 - 670	469.2368	936.4591	936.4586	0.46	0	79	1.1e-08	1	U	H.SGMDTILGK.G + Oxidation (M)
2340	673 - 686	771.3841	1540.7537	1540.7555	-1.20	1	120	9.7e-13	1	U	K.KDASTLMTYAVADR.F
2341	673 - 686	514.5927	1540.7562	1540.7555	0.47	1	70	1e-07	1	U	K.KDASTLMTYAVADR.F
2356	673 - 686	519.9238	1556.7495	1556.7504	-0.62	1	69	1.1e-07	1	U	K.KDASTLMTYAVADR.F + Oxidation (M)
2357	673 - 686	779.3828	1556.7510	1556.7504	0.39	1	126	2.4e-13	1	U	K.KDASTLMTYAVADR.F + Oxidation (M)
2089	674 - 686	715.3319	1428.6493	1428.6555	-4.35	0	102	6.5e-11	1	U	K.DASTLMTYAVADR.F + Oxidation (M)
2092	674 - 686	715.3337	1428.6528	1428.6555	-1.91	0	31	0.00081	1	U	K.DASTLMTYAVADR.F + Oxidation (M)
2094	674 - 686	715.3341	1428.6537	1428.6555	-1.27	0	88	1.6e-09	1	U	K.DASTLMTYAVADR.F + Oxidation (M)

Query	Start – End	Observed	Mr(expt)	Mr(calc)	ppm	M	Score	Expect	Rank	U	Peptide
2095	674 – 686	715.3352	1428.6558	1428.6555	0.21	0	93	6.7e-10	1	U	K.DASTLMTYAVADR.F + Oxidation (M)
1389	676 – 686	622.3053	1242.5961	1242.5914	3.74	0	42	6.7e-05	1	U	A.STLMTYAVADR.F + Oxidation (M)
1158	677 – 686	578.7878	1155.5610	1155.5594	1.35	0	64	5.2e-07	1	U	S.TLMTYAVADR.F + Oxidation (M)
1573	694 – 704	646.3739	1290.7333	1290.7336	-0.21	0	56	2.4e-06	1	U	K.LGFLITQSVWK.T
1070	696 – 704	561.3240	1120.6335	1120.6281	4.81	0	43	5.3e-05	1	U	G.FLITQSVWK.T
129	705 – 712	397.1999	792.3852	792.3879	-3.36	0	71	7.5e-08	1	U	K.TGAGQGFR.R
130	705 – 712	397.2013	792.3881	792.3879	0.35	0	61	7.2e-07	1	U	K.TGAGQGFR.R
131	705 – 712	397.2015	792.3885	792.3879	0.83	0	41	8.8e-05	1	U	K.TGAGQGFR.R
132	705 – 712	397.2016	792.3886	792.3879	0.90	0	53	4.7e-06	1	U	K.TGAGQGFR.R
133	705 – 712	397.2022	792.3898	792.3879	2.49	0	64	3.7e-07	1	U	K.TGAGQGFR.R
25	716 – 722	362.1752	722.3358	722.3347	1.46	0	56	2.5e-06	1	U	R.IGENGPH.L
650	716 – 724	331.5124	991.5154	991.5199	-4.57	0	44	4.3e-05	1	U	R.IGENGPHLR.V
651	716 – 724	331.5128	991.5166	991.5199	-3.30	0	40	0.0001	1	U	R.IGENGPHLR.V
652	716 – 724	496.7663	991.5180	991.5199	-1.93	0	81	8.7e-09	1	U	R.IGENGPHLR.V
653	716 – 724	496.7671	991.5196	991.5199	-0.27	0	46	2.7e-05	1	U	R.IGENGPHLR.V
356	717 – 724	440.2251	878.4357	878.4359	-0.22	0	51	1.3e-05	1	U	I.GENGPHLR.V
2224	725 – 737	736.3927	1470.7709	1470.7719	-0.69	0	61	8.5e-07	1	U	R.VLHVDDLSSLQVF.E
2590	725 – 740	864.9437	1727.8729	1727.8730	-0.050	0	70	1.3e-07	1	U	R.VLHVDDLSSLQVFEGA.S
2697	725 – 741	908.4570	1814.8994	1814.9051	-3.09	0	76	3.4e-08	1	U	R.VLHVDDLSSLQVFEGAS.T
3084	725 – 743	1037.0321	2072.0496	2072.0538	-2.03	0	155	3.3e-16	1	U	R.VLHVDDLSSLQVFEGASTR.T
3085	725 – 743	691.6920	2072.0543	2072.0538	0.22	0	83	5.2e-09	1	U	R.VLHVDDLSSLQVFEGASTR.T
3086	725 – 743	691.6925	2072.0558	2072.0538	0.94	0	78	1.6e-08	1	U	R.VLHVDDLSSLQVFEGASTR.T
3088	725 – 743	691.6933	2072.0580	2072.0538	2.01	0	69	1.4e-07	1	U	R.VLHVDDLSSLQVFEGASTR.T
2585	728 – 743	862.4283	1722.8420	1722.8425	-0.29	0	93	5.7e-10	1	U	H.VDDLSSLQVFEGASTR.T
2287	730 – 743	755.3813	1508.7480	1508.7471	0.62	0	42	6.4e-05	1	U	D.DLSSLQVFEGASTR.T
1537	732 – 743	641.3256	1280.6366	1280.6361	0.38	0	60	9.8e-07	1	U	L.SSLQVFEGASTR.T
1234	733 – 743	597.8100	1193.6055	1193.6040	1.17	0	59	1.6e-06	1	U	S.SLQVFEGASTR.T
525	744 – 752	475.7683	949.5220	949.5233	-1.36	1	62	6e-07	1	U	R.TSAFVLQKG.R
1309	757 – 765	608.8035	1215.5924	1215.5964	-3.32	0	68	2e-07	1	U	R.YPVPYTYWK.K
3327	767 – 787	773.3609	2317.0610	2317.0631	-0.94	1	100	1.3e-10	1	U	K.TTKGEGLDYDSTLGEVMEQTK.R + Oxidation (M)
2982	770 – 787	994.4400	1986.8654	1986.8728	-3.75	0	127	1.8e-13	1	U	K.GEGLDYDSTLGEVMEQTK.R + Oxidation (M)
2983	770 – 787	994.4404	1986.8662	1986.8728	-3.33	0	36	0.00022	1	U	K.GEGLDYDSTLGEVMEQTK.R + Oxidation (M)
2985	770 – 787	994.4427	1986.8709	1986.8728	-0.97	0	101	7.2e-11	1	U	K.GEGLDYDSTLGEVMEQTK.R + Oxidation (M)
2986	770 – 787	994.4430	1986.8714	1986.8728	-0.73	0	109	1.3e-11	1	U	K.GEGLDYDSTLGEVMEQTK.R + Oxidation (M)
2987	770 – 787	663.2984	1986.8733	1986.8728	0.27	0	92	6.1e-10	1	U	K.GEGLDYDSTLGEVMEQTK.R + Oxidation (M)
3190	770 – 788	715.3296	2142.9669	2142.9739	-3.27	1	107	1.8e-11	1	U	K.GEGLDYDSTLGEVMEQTKR.L + Oxidation (M)
3191	770 – 788	715.3302	2142.9688	2142.9739	-2.37	1	51	8.5e-06	1	U	K.GEGLDYDSTLGEVMEQTKR.L + Oxidation (M)
3192	770 – 788	1072.4917	2142.9689	2142.9739	-2.36	1	107	1.9e-11	1	U	K.GEGLDYDSTLGEVMEQTKR.L + Oxidation (M)
3194	770 – 788	715.3318	2142.9737	2142.9739	-0.12	1	114	4.4e-12	1	U	K.GEGLDYDSTLGEVMEQTKR.L + Oxidation (M)
3196	770 – 788	715.3322	2142.9748	2142.9739	0.41	1	40	0.0001	1	U	K.GEGLDYDSTLGEVMEQTKR.L + Oxidation (M)
3197	770 – 788	715.3326	2142.9759	2142.9739	0.90	1	63	4.9e-07	1	U	K.GEGLDYDSTLGEVMEQTKR.L + Oxidation (M)
841	779 – 787	525.7620	1049.5095	1049.5063	3.01	0	42	0.00012	1	U	T.LGEVMEQTK.R + Oxidation (M)
1682	791 – 802	663.3232	1324.6319	1324.6300	1.46	0	49	1.4e-05	1	U	R.FHAVPVDPDDLT.S
2540	791 – 805	848.4024	1694.7903	1694.7941	-2.21	0	56	2.8e-06	1	U	R.FHAVPVDPDDLTSPW.L
2686	791 – 806	904.9445	1807.8744	1807.8781	-2.05	0	47	2.8e-05	1	U	R.FHAVPVDPDDLTSPWL.T
2862	791 – 807	955.4680	1908.9215	1908.9258	-2.26	0	30	0.00091	1	U	R.FHAVPVDPDDLTSPWLT.A
3174	791 – 809	713.0254	2136.0543	2136.0640	-4.54	0	116	2.2e-12	1	U	R.FHAVPVDPDDLTSPWLTAR.R
3175	791 – 809	1069.0359	2136.0572	2136.0640	-3.22	0	145	3.3e-15	1	U	R.FHAVPVDPDDLTSPWLTAR.R
3176	791 – 809	713.0277	2136.0614	2136.0640	-1.24	0	85	4.1e-09	1	U	R.FHAVPVDPDDLTSPWLTAR.R
3177	791 – 809	713.0288	2136.0646	2136.0640	0.29	0	92	8.3e-10	1	U	R.FHAVPVDPDDLTSPWLTAR.R
2738	793 – 809	926.9736	1851.9327	1851.9367	-2.14	0	147	2.9e-15	1	U	H.AVPVDPDDLTSPWLTAR.R
1870	798 – 809	686.3493	1370.6841	1370.6830	0.77	0	97	2.4e-10	1	U	D.PDDLTSPWLTAR.R
282	811 – 817	424.7578	847.5011	847.5028	-1.96	1	53	5.1e-06	1	U	R.RALYAVR.K
852	818 – 826	526.7899	1051.5653	1051.5662	-0.89	1	72	6.6e-08	1	U	R.KVLGTSEYR.A
853	818 – 826	526.7901	1051.5657	1051.5662	-0.43	1	41	7.6e-05	1	U	R.KVLGTSEYR.A
15	857 – 863	359.6903	717.3661	717.3657	0.60	0	44	9.8e-05	1	U	R.NVTEGAK.R
3266	865 – 883	1100.5860	2199.1575	2199.1674	-4.53	0	64	4.1e-07	1	U	R.EVEGITTELEPDLLYPLLR.G
3267	865 – 883	1100.5889	2199.1633	2199.1674	-1.89	0	138	1.8e-14	1	U	R.EVEGITTELEPDLLYPLLR.G
3268	865 – 883	734.0631	2199.1676	2199.1674	0.062	0	93	4.8e-10	1	U	R.EVEGITTELEPDLLYPLLR.G
374	877 – 883	444.2884	886.5623	886.5640	-1.91	0	54	3.9e-06	1	U	D.LLYPLLR.G
1161	889 – 897	386.5329	1156.5768	1156.5778	-0.79	1	34	0.00036	1	U	R.RWYAQPSLH.I
680	890 – 897	501.2453	1000.4761	1000.4767	-0.58	0	53	5.1e-06	1	U	R.WYAQPSLH.I
2922	890 – 905	648.0009	1940.9808	1940.9818	-0.54	0	68	1.6e-07	1	U	R.WYAQPSLHILMVQDPK.T + Oxidation (M)
2923	890 – 905	971.4977	1940.9809	1940.9818	-0.50	0	30	0.0009	1	U	R.WYAQPSLHILMVQDPK.T + Oxidation (M)

Query	Start - End	Observed	Mr(expt)	Mr(calc)	ppm	M	Score	Expect	Rank	U	Peptide
2401	892 - 905	531.6209	1591.8410	1591.8392	1.13	0	57	2e-06	1	U	Y.AQPSLHILMVQDPK.T + Oxidation (M)
1276	896 - 905	403.8938	1208.6596	1208.6587	0.70	0	40	0.00012	1	U	S.LHILMVQDPK.T + Oxidation (M)
489	898 - 905	472.2681	942.5217	942.5208	0.90	0	67	2.7e-07	1	U	H.ILMVQDPK.T
554	898 - 905	480.2642	958.5139	958.5157	-1.88	0	77	2.2e-08	1	U	H.ILMVQDPK.T + Oxidation (M)
1216	908 - 917	395.8902	1184.6488	1184.6513	-2.12	1	51	7.7e-06	1	U	R.RGIDEQVLQK.R
1217	908 - 917	593.3318	1184.6491	1184.6513	-1.84	1	58	1.5e-06	1	U	R.RGIDEQVLQK.R
773	909 - 917	515.2814	1028.5483	1028.5502	-1.87	0	38	0.00017	1	U	R.GIDEQVLQK.R
774	909 - 917	515.2826	1028.5506	1028.5502	0.41	0	54	4.5e-06	1	U	R.GIDEQVLQK.R
775	909 - 917	515.2829	1028.5513	1028.5502	1.03	0	42	7.3e-05	1	U	R.GIDEQVLQK.R
1219	909 - 918	593.3331	1184.6516	1184.6513	0.22	1	70	9.2e-08	1	U	R.GIDEQVLQKR.Y
1220	909 - 918	395.8911	1184.6516	1184.6513	0.23	1	46	2.7e-05	1	U	R.GIDEQVLQKR.Y
376	928 - 934	445.7625	889.5105	889.5134	-3.19	1	38	0.00015	1	U	K.RFEAVLR.E
45	929 - 934	367.7134	733.4122	733.4123	-0.095	0	36	0.00026	1	U	R.FEAVLR.E
46	929 - 934	367.7135	733.4125	733.4123	0.31	0	43	5.6e-05	1	U	R.FEAVLR.E
47	929 - 934	367.7139	733.4132	733.4123	1.30	0	46	2.4e-05	1	U	R.FEAVLR.E
1681	952 - 962	662.7825	1323.5504	1323.5516	-0.86	0	34	0.00038	1	U	R.MVETGPFYSMF.N + Oxidation (M)
1723	952 - 962	670.7792	1339.5439	1339.5465	-1.92	0	56	2.5e-06	1	U	R.MVETGPFYSMF.N + 2 Oxidation (M)
2174	952 - 963	727.8035	1453.5924	1453.5894	2.07	0	57	2.1e-06	1	U	R.MVETGPFYSMFN.V + 2 Oxidation (M)
2725	959 - 973	921.4131	1840.8116	1840.8131	-0.79	0	105	3.5e-11	1	U	F.YSMFNVGDYTFAPWK.V + Oxidation (M)
2148	962 - 973	722.8500	1443.6854	1443.6823	2.11	0	43	4.7e-05	1	U	M.FNVGDYTFAPWK.V
1589	963 - 973	649.3137	1296.6128	1296.6139	-0.81	0	66	2.7e-07	1	U	F.NVGDYTFAPWK.V
1214	964 - 973	592.2930	1182.5714	1182.5710	0.34	0	83	5.2e-09	1	U	N.VGDYTFAPWK.V
424	967 - 973	456.7342	911.4538	911.4541	-0.38	0	34	0.00036	1	U	D.YTFAPWK.V
2760	978 - 995	933.9780	1865.9414	1865.9411	0.17	0	120	9.3e-13	1	U	R.YVASDFIVAVVGPASDEK.P
3342	978 - 1000	1187.1173	2372.2200	2372.2264	-2.68	0	51	8.8e-06	1	U	R.YVASDFIVAVVGPASDEKPVVPN.E
3344	978 - 1000	791.7486	2372.2241	2372.2264	-0.99	0	86	2.3e-09	1	U	R.YVASDFIVAVVGPASDEKPVVPN.E
3376	978 - 1002	1315.6832	2629.3519	2629.3639	-4.56	0	69	1.3e-07	1	U	R.YVASDFIVAVVGPASDEKPVVPNEK.L
3377	978 - 1002	877.4581	2629.3525	2629.3639	-4.37	0	152	7e-16	1	U	R.YVASDFIVAVVGPASDEKPVVPNEK.L
3379	978 - 1002	877.4616	2629.3631	2629.3639	-0.32	0	113	5.2e-12	1	U	R.YVASDFIVAVVGPASDEKPVVPNEK.L
3380	978 - 1002	877.4617	2629.3632	2629.3639	-0.27	0	110	1e-11	1	U	R.YVASDFIVAVVGPASDEKPVVPNEK.L
3381	978 - 1002	658.3484	2629.3647	2629.3639	0.28	0	82	6.6e-09	1	U	R.YVASDFIVAVVGPASDEKPVVPNEK.L
108	996 - 1002	391.7244	781.4343	781.4334	1.14	0	57	2e-06	1	U	K.PVVPNEK.L
2279	1003 - 1015	754.3523	1506.6900	1506.6912	-0.81	0	32	0.001	1	U	K.LMLVPVEDDNEAF.Y + Oxidation (M)
1201	1028 - 1037	587.3009	1172.5873	1172.5866	0.57	0	38	0.00023	1	U	R.FAVQSFFVQT.Q
1603	1028 - 1038	651.3292	1300.6438	1300.6452	-1.06	0	43	5.1e-05	1	U	R.FAVQSFFVQTQ.I
2579	1028 - 1042	860.4431	1718.8716	1718.8781	-3.76	0	100	1.4e-10	1	U	R.FAVQSFFVQTQIAPH.V
2580	1028 - 1042	573.9658	1718.8755	1718.8781	-1.51	0	95	4e-10	1	U	R.FAVQSFFVQTQIAPH.V
3256	1028 - 1046	730.0671	2187.1795	2187.1841	-2.10	0	105	3e-11	1	U	R.FAVQSFFVQTQIAPHVLQK.L
2284	1034 - 1046	754.9331	1507.8517	1507.8511	0.41	0	85	3e-09	1	U	F.FVQTQIAPHVLQK.L
2285	1034 - 1046	503.6248	1507.8525	1507.8511	0.92	0	64	3.8e-07	1	U	F.FVQTQIAPHVLQK.L
408	1039 - 1046	302.5243	904.5509	904.5494	1.68	0	49	1.3e-05	1	U	Q.IAPHVLQK.L
1060	1052 - 1060	559.2315	1116.4484	1116.4472	1.11	0	51	8e-06	1	U	R.YEPNTDHQN.R
1510	1052 - 1061	637.2805	1272.5464	1272.5483	-1.51	0	89	1.3e-09	1	U	R.YEPNTDHQNR.I
1511	1052 - 1061	425.1905	1272.5496	1272.5483	0.99	0	56	2.6e-06	1	U	R.YEPNTDHQNR.I
1512	1052 - 1061	425.1906	1272.5500	1272.5483	1.30	0	46	2.3e-05	1	U	R.YEPNTDHQNR.I
615	1054 - 1061	491.2282	980.4418	980.4424	-0.65	0	59	1.8e-06	1	U	E.PNTDHQNR.I
2292	1068 - 1081	378.9440	1511.7469	1511.7480	-0.79	1	64	3.9e-07	1	U	R.RAHELAPAAYNGDK.A
2293	1068 - 1081	504.9231	1511.7475	1511.7480	-0.38	1	97	2.2e-10	1	U	R.RAHELAPAAYNGDK.A
2294	1068 - 1081	756.8816	1511.7487	1511.7480	0.41	1	69	1.4e-07	1	U	R.RAHELAPAAYNGDK.A
1781	1069 - 1081	452.8884	1355.6435	1355.6469	-2.56	0	91	7.9e-10	1	U	R.AHELAPAAYNGDK.A
1782	1069 - 1081	452.8886	1355.6439	1355.6469	-2.25	0	61	8.3e-07	1	U	R.AHELAPAAYNGDK.A
817	1089 - 1096	349.1796	1044.5171	1044.5200	-2.78	1	47	2e-05	1	U	R.RVEEEIDR.A
818	1089 - 1096	523.2679	1044.5212	1044.5200	1.17	1	60	1.2e-06	1	U	R.RVEEEIDR.A
821	1089 - 1096	349.1823	1044.5251	1044.5200	4.95	1	37	0.00025	1	U	R.RVEEEIDR.A
375	1090 - 1096	445.2152	888.4159	888.4189	-3.28	0	63	7.2e-07	1	U	R.VEEEIDR.A
2831	1097 - 1113	950.4915	1898.9685	1898.9737	-2.77	0	103	4.8e-11	1	U	R.AAAQLWGLTEEELAEIR.R
2832	1097 - 1113	633.9983	1898.9730	1898.9737	-0.41	0	132	6.3e-14	1	U	R.AAAQLWGLTEEELAEIR.R
3063	1097 - 1114	1028.5431	2055.0717	2055.0748	-1.54	1	44	4.4e-05	1	U	R.AAAQLWGLTEEELAEIRR.S
3064	1097 - 1114	686.0315	2055.0726	2055.0748	-1.09	1	82	6.3e-09	1	U	R.AAAQLWGLTEEELAEIRR.S
2048	1103 - 1114	472.5882	1414.7427	1414.7416	0.81	1	48	1.7e-05	1	U	W.GLTEEELAEIRR.S
57	1115 - 1120	373.7055	745.3964	745.3970	-0.83	0	53	1.3e-05	1	U	R.SLEELR.G
160	1115 - 1121	402.2160	802.4175	802.4185	-1.19	1	42	0.00014	1	U	R.SLEELRG.-
161	1115 - 1121	402.2171	802.4196	802.4185	1.43	1	46	6.3e-05	1	U	R.SLEELRG.-

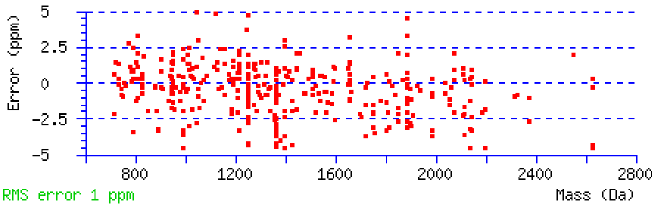

Mascot: http://www.matrixscience.com/
