# Supplementary figures and images for: Thermostable proteins bioprocesses: The activity of restriction endonuclease-methyltransferase from Thermus thermophilus (RM.TthHB27I) cloned in Escherichia coli is critically affected by the codon composition of the synthetic gene
Source: PLoS One. 2017 Oct 17;12(10):e0186633. doi: 10.1371/journal.pone.0186633 (PMC5645126; doi:10.1371/journal.pone.0186633)

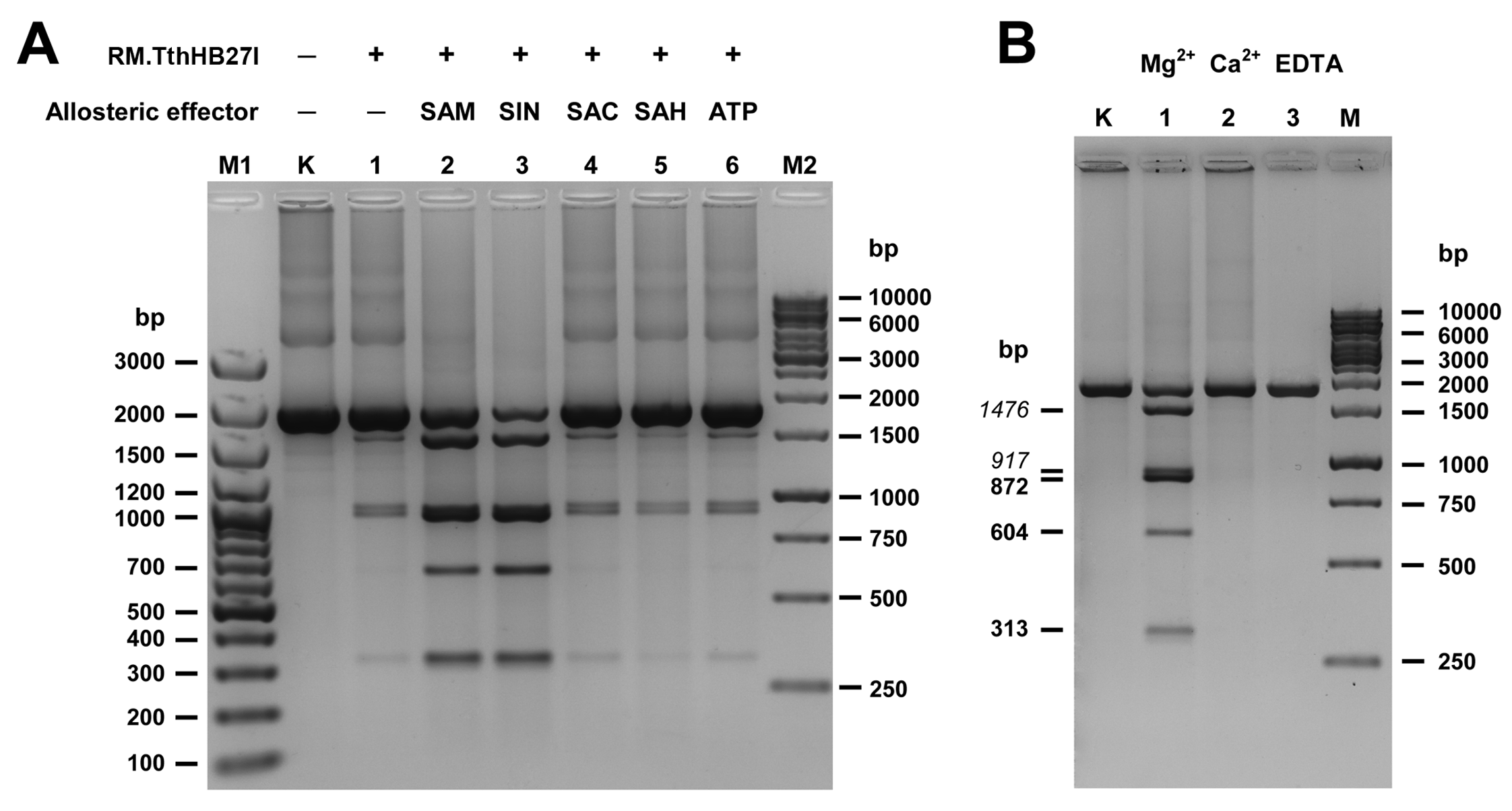

Supplement: S6 File — (A) Effect of cofactor or its analogues on synthetic RM.TthHB27I REase activity. 0.5 μg of 1789 bp PCR DNA substrate was digested with 2 units of synthetic RM.TthHB27I in REase buffer supplemented with 50 μM of the selected effector at 65°C. Lane M1, 100 bp Plus DNA Ladder; lane M2, GeneRuler 1 kb DNA Ladder, lane K, untreated PCR fragment; lane 1, PCR fragment digested with synthetic RM.TthHB27I, no allosteric effector; lane 2, as in lane 1, supplemented with SAM; lane 3, supplemented with SIN; lane 4, supplemented with SAC; lane 5, supplemented with SAH; lane 6, supplemented with ATP. (B) MTase activity assay. Lane M, GeneRuler 1 kb DNA Ladder (Thermo Fisher Scientific/Fermentas); lane K, untreated 1789 bp PCR DNA substrate; lane 1, incubation with synthetic RM.TthHB27I in the MTase base buffer supplemented with 100 μM SAM and 6 mM Mg2+; lane 2, as in lane 1, supplemented with 6 mM Ca2+ instead of Mg2+ ions; lane 3, supplemented with 3 mM EDTA instead of Mg2+ ions. The complete digestion pattern includes restriction fragments of 872, 602 and 311 bp (bold). Fragments indicated in italics (1476 and 915 bp) are a result of incomplete digestion. (TIF) [file pone.0186633.s006.tif]
