# Supplementary material for: Thermostable proteins bioprocesses: The activity of restriction endonuclease-methyltransferase from Thermus thermophilus (RM.TthHB27I) cloned in Escherichia coli is critically affected by the codon composition of the synthetic gene
Source: PLoS One. 2017 Oct 17;12(10):e0186633. doi: 10.1371/journal.pone.0186633 (PMC5645126; doi:10.1371/journal.pone.0186633)
Supplement: S1 Table — (DOCX) [file pone.0186633.s007.docx]

**S1 Table. Codon distribution within the wt *tthHB27IRM* and the synthetic *tthHB27IRM* sequences.**

| **aa** | **codon** | **Fraction in**  ***E. coli*^1^** | **wt *tthHB27IRM*** | **synthetic *tthHB27IRM*** | **Fraction in synthetic**  ***tthHB27IRM*** | **aa** | **codon** | **Fraction in**  ***E. coli*^1^** | **wt *tthHB27IRM*** | **synthetic  *tthHB27IRM*** | **Fraction in synthetic**  ***tthHB27IRM*** |
| --- | --- | --- | --- | --- | --- | --- | --- | --- | --- | --- | --- |
| **Ala (A)** | GCU  GCA GCG  GCC | 0.35  0.28  0.28  0.10 | 15  4  15  65 | 35  37  27  0 | 0.35  0.37  0.27  0.00 | **Leu (L)** | CUG  CUC  CUU  UUG  UUA  CUA | 0.83  0.07  0.04  0.03  0.02  0.00 | 74  35  14  13  3  5 | 129  15  0  0  0  0 | 0.90  0.10  0.00  0.00  0.00  0.00 |
| **Arg (R)** | CGU  CGC  CGA  AGG  AGA  CGG | 0.74  0.25  0.01  0.00  0.00  0.00 | 11  47  2  8  2  30 | 70  30  0  0  0  0 | 0.70  0.30  0.00  0.00  0.00  0.00 | **Lys (K)** | AAA  AAG | 0.74  0.26 | 15  24 | 28  11 | 0.72  0.28 |
| **Asn (N)** | AAC  AAU | 0.94  0.06 | 31  9 | 35  5 | 0.88  0.12 | **Met (M)** | AUG | 1 | 15 | 15 | 1.00 |
| **Asp (D)** | GAC  GAU | 0.67  0.33 | 44  12 | 36  20 | 0.64  0.36 | **Phe (F)** | UUC  UUU | 0.76  0.24 | 36  16 | 38  14 | 0.73  0.27 |
| **Cys (C)** | UGC  UGU | 0.51  0.49 | 4  2 | 3  3 | 0.50  0.50 | **Pro (P)** | CCG  CCA  CCU  CCC | 0.77  0.15  0.08  0.00 | 17  4  6  31 | 46  12  0  0 | 0.79  0.21  0.00  0.00 |
| **Gln (Q)** | CAG  CAA | 0.86  0.14 | 22  14 | 31  5 | 0.86  0.14 | **Ser (S)** | UCC  UCU  AGC  UCG  AGU  UCA | 0.37  0.34  0.20  0.04  0.03  0.02 | 12  7  18  4  6  1 | 21  21  6  0  0  0 | 0.44  0.44  0.12  0.00  0.00  0.00 |
| **Glu (E)** | GAA  GAG | 0.78  0.22 | 35  52 | 66  21 | 0.76  0.24 | **Thr (T)** | ACC  ACU  ACG  ACA | 0.55  0.35  0.07  0.04 | 34  6  10  4 | 33  21  0  0 | 0.61  0.39  0.00  0.00 |
| **Gly (G)** | GGU  GGC  GGG  GGA | 0.59  0.39  0.02  0.00 | 9  42  20  7 | 48  30  0  0 | 0.62  0.38  0.00  0.00 | **Trp (W)** | UGG | 1 | 21 | 21 | 1.00 |
| **His (H)** | CAC  CAU | 0.83  0.17 | 18  10 | 24  4 | 0.86  0.14 | **Tyr (Y)** | UAC  UAU | 0.75  0.25 | 38  5 | 33  10 | 0.77  0.23 |
| **Ile (I)** | AUC  AUU  AUA | 0.83  0.17  0.00 | 23  12  5 | 34  6  0 | 0.85  0.15  0.00 | **Val (V)** | GUU  GUA  GUG  GUC | 0.51  0.26  0.16  0.07 | 8  3  46  20 | 52  25  0  0 | 0.68  0.32  0.00  0.00 |

Codons biased toward low GC content are underlined.

^1^ Fraction of relative occurrences of the codon in its synonymous codon family [30].
