# Supplementary material for: Thermostable proteins bioprocesses: The activity of restriction endonuclease-methyltransferase from Thermus thermophilus (RM.TthHB27I) cloned in Escherichia coli is critically affected by the codon composition of the synthetic gene
Source: PLoS One. 2017 Oct 17;12(10):e0186633. doi: 10.1371/journal.pone.0186633 (PMC5645126; doi:10.1371/journal.pone.0186633)
Supplement: S2 Table — (DOCX) [file pone.0186633.s008.docx]

**S2 Table. Codon usage data from highly expressed subset of genes in *E. coli*.**

| **aa** | **codon** | **Sharp et al., 1986**  **[31]** | | **Sharp et al., 1988 [32]** | **The Wisconsin Package, GCG, 1992 [30]** | **Sharp et al.,**  **2010 [33]** |
| --- | --- | --- | --- | --- | --- | --- |
|  |  | **VH** | **H** | **H** | **VH** | **H** |
| **Ala (A)** | GCU  GCA  GCG  GCC | 0.47  0.27  0.20  0.06 | 0.23  0.23  0.37  0.17 | 0.35  0.28  0.28  0.10 | 0.51  0.27  0.18  0.04 | 0.46  0.27  0.19  0.08 |
| **Arg (R)** | CGU  CGC  CGA  AGG  AGA  CGG | 0.73  0.26  0.00  0.00  0.00  0.00 | 0.64  0.33  0.01  0.01  0.00  0.01 | 0.74  0.25  0.01  0.00  0.00  0.00 | 0.75  0.25  0.00  0.00  0.00  0.00 | 0.69  0.30  0.00  0.00  0.00  0.00 |
| **Asn (N)** | AAC  AAU | 0.95  0.05 | 0.82  0.18 | 0.94  0.06 | 0.99  0.01 | 0.88  0.12 |
| **Asp (D)** | GAC  GAU | 0.70  0.30 | 0.53  0.47 | 0.67  0.33 | 0.74  0.26 | 0.66  0.34 |
| **Cys (C)** | UGC  UGU | 0.66  0.34 | 0.62  0.38 | 0.51  0.49 | 0.70  0.30 | 0.62  0.38 |
| **Gln (Q)** | CAG  CAA | 0.89  0.11 | 0.82  0.18 | 0.86  0.14 | 0.94  0.06 | 0.82  0.18 |
| **Glu (E)** | GAA  GAG | 0.80  0.20 | 0.72  0.28 | 0.78  0.22 | 0.82  0.18 | 0.76  0.24 |
| **Gly (G)** | GGU  GGC  GGG  GGA | 0.57  0.41  0.01  0.01 | 0.56  0.38  0.05  0.02 | 0.59  0.39  0.02  0.00 | 0.57  0.42  0.01  0.00 | 0.63  0.35  0.01  0.00 |
| **His (H)** | CAC  CAU | 0.78  0.22 | 0.72  0.28 | 0.83  0.17 | 0.78  0.22 | 0.70  0.30 |
| **Ile (I)** | AUC  AUU  AUA | 0.84  0.16  0.00 | 0.68  0.32  0.00 | 0.83  0.17  0.00 | 0.84  0.16  0.00 | 0.76  0.24  0.00 |
| **Leu (L)** | CUG  CUC  CUU  UUG  UUA  CUA | 0.89  0.03  0.04  0.02  0.02  0.01 | 0.77  0.08  0.06  0.06  0.03  0.01 | 0.83  0.07  0.04  0.03  0.02  0.00 | 0.92  0.03  0.02  0.01  0.01  0.01 | 0.85  0.04  0.05  0.04  0.02  0.00 |
| **Lys (K)** | AAA  AAG | 0.80  0.20 | 0.72  0.28 | 0.74  0.26 | 0.82  0.18 | 0.72  0.28 |
| **Met (M)** | AUG | 1.00 | 1.00 | 1.00 | 1.00 | 1.00 |
| **Phe (F)** | UUC  UUU | 0.77  0.23 | 0.70  0.30 | 0.76  0.24 | 0.83  0.17 | 0.78  0.22 |
| **Pro (P)** | CCG  CCA  CCU  CCC | 0.82  0.11  0.06  0.01 | 0.74  0.12  0.12  0.02 | 0.77  0.15  0.08  0.00 | 0.85  0.11  0.04  0.00 | 0.71  0.13  0.15  0.01 |
| **Ser (S)** | UCC  UCU  AGC  UCG  AGU  UCA | 0.32  0.43  0.18  0.01  0.04  0.03 | 0.29  0.29  0.25  0.08  0.04  0.04 | 0.37  0.34  0.20  0.04  0.03  0.02 | 0.35  0.47  0.15  0.00  0.02  0.01 | 0.25  0.42  0.23  0.01  0.05  0.03 |
| **Thr (T)** | ACC  ACU  ACG  ACA | 0.47  0.45  0.04  0.04 | 0.59  0.24  0.13  0.03 | 0.55  0.35  0.07  0.04 | 0.48  0.47  0.03  0.02 | 0.44  0.47  0.04  0.05 |
| **Trp (W)** | UGG | 1.00 | 1.00 | 1.00 | 1.00 | 1.00 |
| **Tyr (Y)** | UAC  UAU | 0.80  0.20 | 0.66  0.34 | 0.75  0.25 | 0.81  0.19 | 0.76  0.24 |
| **Val (V)** | GUU  GUA  GUG  GUC | 0.56  0.28  0.12  0.04 | 0.38  0.22  0.27  0.13 | 0.51  0.26  0.16  0.07 | 0.60  0.28  0.10  0.02 | 0.52  0.28  0.12  0.08 |
| HV - very highly expressed genes  H - highly expressed genes | | | | | | |
